# Abietane-Type Diterpenoids from the Arils of *Torreya grandis*

**DOI:** 10.3390/molecules29091905

**Published:** 2024-04-23

**Authors:** Yuqi Gao, Jinghui Yang, Yue Zhang, Linlin Gao, Junmian Tian, Wenbo Han, Jinming Gao

**Affiliations:** 1 College of Food Science and Technology, Northwest University, Xi’an 710075, China; gaoyuqi1989@gmail.com; 2 Shaanxi Key Laboratory of Natural Products & Chemical Biology, College of Chemistry & Pharmacy, Northwest A&F University, Yangling 712100, China; 2020056692@nwafu.edu.cn (J.Y.); zhangyuey@nwafu.edu.cn (Y.Z.); linlingaoluck@163.com (L.G.); jinminggao@nwsuaf.edu.cn (J.G.)

**Keywords:** diterpenoids, structure elucidation, antibacterial, anti-neuroinflammatory, *Torreya grandis*

## Abstract

A chemical investigation of the arils of *Torreya grandis* led to the isolation of seven abietane-type diterpenoids (compounds **1**–**7**) including three previously undescribed compounds, one unreported natural product, and three known analogs. The structures of these compounds were determined by means of spectroscopy, single-crystal X-ray diffraction, and ECD spectra. An antibacterial activity assay showed that compounds **5** and **6** had significant inhibitory effects on methicillin-resistant *Staphylococcus aureus*, with MIC values of 100 μM. Moreover, compounds **1**, **3**, **4**, and **7** exhibited anti-neuroinflammatory activity in LPS-stimulated BV-2 microglia cells, with the IC_50_ values ranging from 38.4 to 67.9 μM.

## 1. Introduction

Taxaceae plants are the economically and medicinally important coniferous evergreen trees that comprise the well-known genera *Taxus*, *Pseudotaxus* and *Austrotaxus*, whereas the genera *Torreya* and *Amentotaxus* attributed to this family have been controversial for a long time due to their close resemblance to the family of Cephalotaxaceae [1]. Among them, *Torreya* is a primitive member of the gymnospermous yew family (Taxaceae), which consists of seven species and is distributed in the Northern Hemisphere, including North America (*T. taxifolia* and *californica*), Japan (*T. nucifera*), and China (*T. fargesii*, *T. grandis*, *T. jackii*, and *T. yunnanensis*) [2]. However, species of the genus *Torreya* are similar in morphology, and the relationship within this genus is still vague [3].

**Figure 1:**
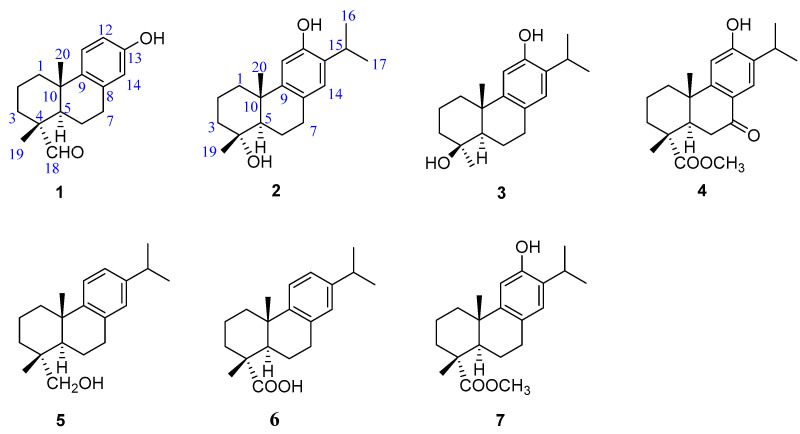
Chemical structures of compounds **1**−**7**.

*Torreya grandis* Fort. ex Lindl cv. Merrillii Hu (Taxaceae), a native Chinese species naturally distributed across subtropical areas in China, has high nutritional and medicinal value [4,5]. Its leaves and fruits are used in traditional Chinese medicine to cure cough, rheumatism, and malnutrition [4]. Recent chemical investigations of *T. grandis* have led to the discovery of structurally diverse phytochemicals, including diterpenoids [6,7], glycosides [5], flavonoids [8], phenols [9], and fatty acids [10], which exhibit various bioactivities such as antibacterial [6], antioxidant [8,9], and antinociceptive and anti-inflammatory effects [11]; attenuation of cognitive impairment [12]; and inhibitory effects against DGAT1/2 [5]. Nevertheless, chemical constituents of the arils of *T. grandis* have rarely been reported [13,14]. As part of our continuing search for novel and/or bioactive metabolites from Chinese medicinal plants [15,16,17], a phytochemical investigation of *T. grandis* arils led to the discovery of seven abietane-type diterpenoids (compounds **1**–**7**), of which compounds **1**–**3** were previously undescribed, compound **4** was an unreported natural product, and compounds **5**–**7** were known analogs (Figure 1). Herein, the isolation, structural analysis, and bioactivities of these products are reported. 

## 2. Results and Discussion

**Figure 2:**
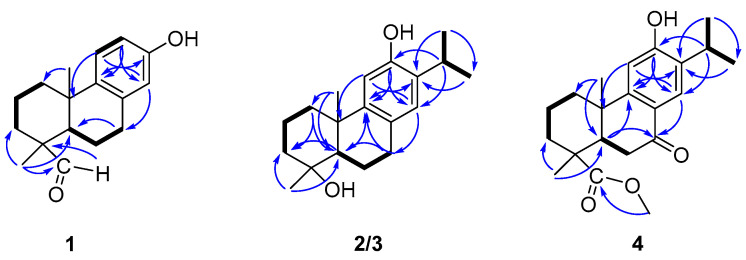
COSY and key HMBC correlations of compounds **1**–**4**.

**Figure 3:**
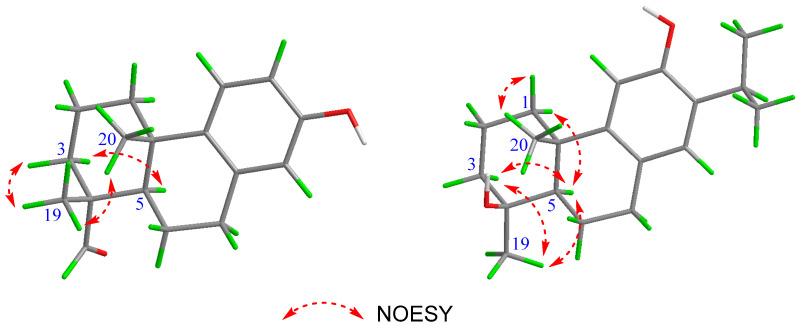
NOESY correlations of compounds **1** (**left**) and **3** (**right**).

**Figure 4:**
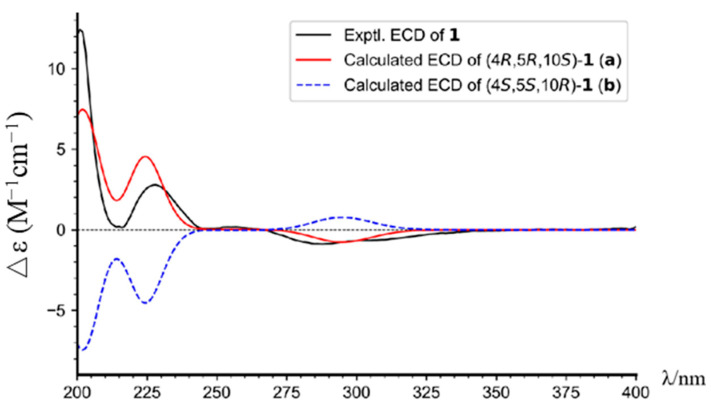
The experimental and calculated ECD spectra of compound **1**.

Torregrandin A (**1**) was obtained as a yellow oil, and its molecular formula was determined as C_17_H_22_O_2_ by a molecular negative-ion peak at *m*/*z* 257.1548 [M − H]^−^ (calcd. for C_17_H_21_O_2_, 257.1547) in its HR-ESI-MS spectrum, requiring 7 degrees of unsaturation. The ^1^H NMR data (Table 1) showed characteristic signals of a 1,2,4-trisubstituted benzene ring at *δ*_H_ 7.05 (d, *J* = 8.6 Hz, 1H), 6.56 (dd, *J* = 8.6, 3.0 Hz, 1H), and 6.43 (d, *J* = 3.0 Hz, 1H), which were supported by the chemical shifts at *δ*_C_ 153.4, 141.5, 136.5, 125.7, 115.2, and 113.3 in the ^13^C NMR spectrum (Table 1). Furthermore, an aldehyde group was indicated by the ^1^H NMR signals at *δ*_H_ 9.20 (s, 1H), corresponding to the carbon signal at *δ*_C_ 206.6 (Table 1). The subsequent interpretation of its 2D NMR spectra (HSQC, COSY, and HMBC) unequivocally underpinned that compound **1** was an abietane-type diterpenoid derivative. Comparing the ^1^H and ^13^C NMR data of compound **1** to that of the known 13-hydroxy-8,11,13-podocarpatrien-18-oic acid [18] revealed that the carboxylic acid group located at C-18 was substituted by the aldehyde group in compound **1**. This was confirmed by the HMBC correlation of H-18 with C-19 and of H_3_-19 with C-18 (Figure 2). Moreover, the HMBC correlations of H_3_-20 with C-1, C-5, and C-9; of H_3_-19 with C-5, C-4, and C-3; of H-14 with C-7, C-9, and C-12; of H-12 with C-9, C-13, and C-14; of H-11 with C-10, C-8, and C-13; and of H-7 with C-5, combined with the ^1^H−^1^H COSY correlations of H_2_-1/H_2_-2/H_2_-3, of H-5/H_2_-6/H_2_-7, and of H-11/H-12 unambiguously established the structure of compound **1** (Figure 2). The NOESY correlations of H-3α (*δ*_H_ 1.27) with H-5 (*δ*_H_ 1.79), of H-3β (*δ*_H_ 1.40) with H_3_-19 (*δ*_H_ 1.09), and of H_3_-19 (*δ*_H_ 1.09) with H_3_-20 (*δ*_H_ 1.13) established the relative configuration of compound **1**, as shown (Figure 3). The similar optical rotations between compound **1** ([α]^25^_D_ +52.0) and 18-oxoferruginol ([α]^25^_D_ +69.6) [19] revealed identical configurations of these two compounds. This finding was reinforced by comparing the experimental and calculated ECD spectra of compound **1** (Figure 4), underscoring its (4*R*,5*R*,10*S*)-configuration.

**Figure 5:**
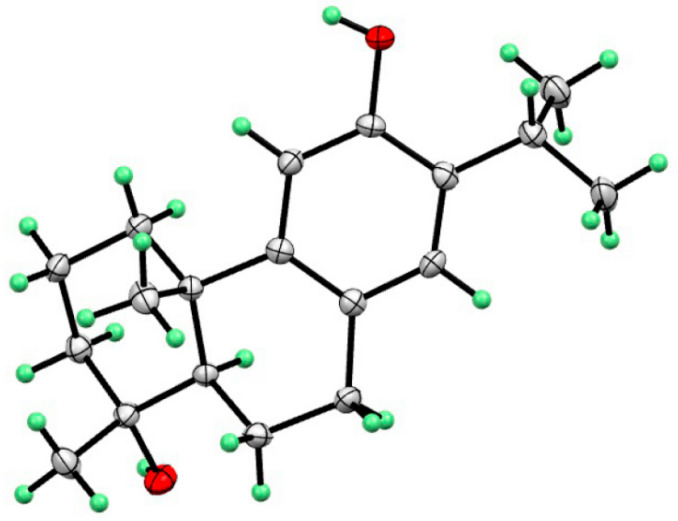
X-ray structure of compound **2**.

Compound **2** was shown to have a molecular formula of C_19_H_27_O_2_ based on its molecular negative-ion peak at *m*/*z* 287.2018 [M − H]^−^ (calcd. for C_19_H_27_O_2_, 287.2017) in its HR-ESI-MS spectrum. The ^1^H and ^13^C NMR data (Table 1) of compound **2** were very similar to those of torregrandol A [20]. Fortunately, compound **2** was crystallized from a methanol solution, which led to the confirmation of its structure and the assignment of its absolute configuration as 4*R*,5*R*,10*S*, based on its single-crystal X-ray diffraction analysis (Cu Kα) (Figure 5). 

**Figure 6:**
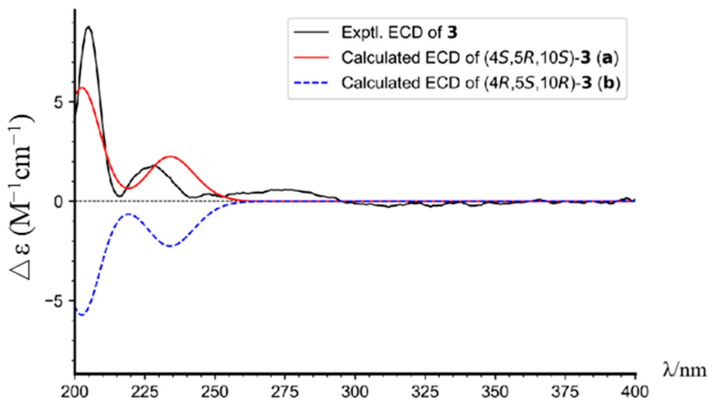
The experimental and calculated ECD spectra of compound **3**.

Torregrandin B (compound **3**) shared an identical molecular formula (C_19_H_27_O_2_) with compound **2**, according to its molecular negative-ion peak at *m*/*z* 287.2021 [M − H]^−^ (calcd. for C_19_H_27_O_2_, 287.2017) in its HR-ESI-MS spectrum. The ^1^H and ^13^C NMR data (Table 2) of this compound were very similar with those of compound **2** except for a downfield signal at *δ*_C_ 30.9, ascribable to the 4-CH_3_ in the ^13^C NMR spectrum. To the best of our knowledge, the carbon resonances of an α-oriented CH_3_ group at C-4 in abietane-type diterpenoids can range from 25 to 35 ppm, whereas in a β-oriented CH_3_ group, they can range from 15 to 25 ppm [6,21]. The downfield chemical shift of 4-CH_3_ (*δ*_C_ 30.9) in comparison with that of compound **2** (*δ*_C_ 22.9) revealed that compound **3** was an epimer of compound **2** at C-4. Next, the NOESY correlations of H-3α (*δ*_H_ 1.72) with H-5 (*δ*_H_ 1.42) and H_3_-19 (*δ*_H_ 1.25), of H-5 (*δ*_H_ 1.42) and H-1β (*δ*_H_ 2.21), and of H-1α (*δ*_H_ 1.42) with H_3_-20 (*δ*_H_ 1.30) established the relative configuration of compound **3**, as shown (Figure 3). The absolute configuration was determined by means of ECD calculation, which unambiguously pinpointed the (4*S*,5*R*,10*S*)-configuration of compound **3** (Figure 6).

**Figure 7:**
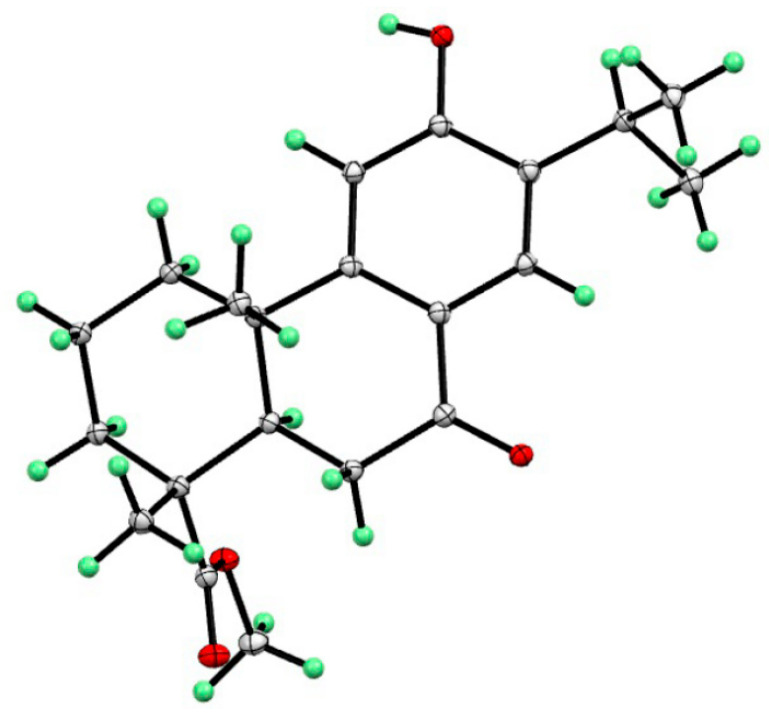
X-ray structure of compound **4**.

Compound **4**, namely methyl 12-hydroxy-7-oxodehydroabietate, was recently isolated from *Torreya grandis* [20], which was previously synthesized by Hamulić and co-workers [22]. However, its absolute configuration was not assigned. In the present work, the absolute configuration of compound **4** was firstly confirmed by the single-crystal X-ray diffraction analysis (Cu Kα) (Figure 7).

The remaining known diterpenoids were characterized as dehydroabietinol (**5**) [23], dehydroabietic acid (**6**) [24], and torreyagrandate (**7**) [25] by comparing the ^1^H and ^13^C NMR data as well as mass spectrometric spectra to the published data.

All isolated compounds were tested for their antibacterial activitity against *Escherichia coli*, methicillin-resistant *Staphylococcus aureus* (MRSA), *Pseudomonas aeruginosa*, and *Salmonella*. However, only compounds **5** and **6** had significant inhibitory effects on MRSA, with MIC values of 100 μM, which were comparable to those of the positive control, rifampicin (MIC = 0.625 μM). Other compounds displayed no obvious antibacterial activity at 100 μM. Moreover, the anti-neuroinflammatory activity in LPS-induced BV-2 cells was evaluated for these compounds. The results indicated that compounds **1**, **3, 4**, and **7** showed a weak inhibitory effect on NO production, with IC_50_ values of 49.4, 41.9, 38.4, and 52.6 μM (Table 3), respectively. Meanwhile, none of the remaining compounds exhibited an inhibitory effect on NO production at 100 μM. All the results are representative of three independent experiments. 

## 3. Materials and Methods

### 3.1. General

A Rudolph Autopol III instrument was used to measure the optical rotations. HR-ESI-MS spectra were tested using a TripleTOF 5600+ system (AB SCIEX, Framingham, MA, USA). Infrared (IR) spectra were acquired using a Bruker TENSOR 27 spectrometer (Bruker). Ultraviolet (UV) and electronic circular dichroism (ECD) spectra were recorded by a Chirascan spectrometer (Applied Photophysics, Ltd., Leatherhead, UK). The NMR data were acquired on a Bruker Avance-400 spectrometer (Beijing Oubeire Co., Ltd., Beijing, China). Column chromatography (CC) was executed using silica (200–300 mesh, Qingdao Marine Chemical Inc., Qingdao, China), C18 reversed-phase silica (ODS-AQ-HG GEL, AQG12S50, YMC, Co., Ltd., Kyoto, Japan), and Sephadex LH-20 gels (GE Healthcare, Inc., Uppsala, Sweden). An Agilent 1100 series system (Agilent Technologies, Inc., Agilent Technologies, Inc., CA, USA) with a Prazis absolute C18 column (5 µm, 10 mm × 250 mm) was used for the HPLC analysis and preparation. Fractions were monitored by TLC (Qingdao Marine Chemical, Ltd., Qingdao, China), with the spots visualized using the vanillin–sulfuric acid color method.

### 3.2. Plant Materials

The arils of *Torreya grandis* Fort. ex Lindl. cv. Merrillii. (Taxaceae) were collected in November of 2022 in Yuexi, Anhui Province, People’s Republic of China. The plant sample was identified by Prof. Zhen-Hai Wu at College of Life Sciences, Northwest A&F University. A voucher specimen has been deposited in our institute with the following accession number: TG-arils-2022-AH.

### 3.3. Extraction and Isolation

The air-dried powder of the arils of *Torreya grandis* (1.5 kg) was presoaked three times with 95% EtOH (3 × 2 L) at room temperature to produce an extract (338 g) which was first re-suspended in H_2_O and then partitioned with petroleum ether (PE), ethyl acetate (EtOAc), and *n*-butanol. The EtOAc extract (75 g) was eluted with PE-EtOAc (*v*/*v*, 50:1→1:1), which was then combined under the guidance of the thin-layer chromatography (TLC) analysis to obtain eight fractions (A–H). Fraction E (11.6 g) was subjected to a silica gel.

Gel CC with PE-EtOAc (*v*/*v*, 10:1→2:1) produced five fractions (E1–E5). Fraction E2 (1.2 g) was isolated on a Sephadex LH-20 column eluted with MeOH to yield five fractions (E2a–E2e). Fraction E2b (300 mg) was separated on a reverse-phase C18 silica gel CC column eluted with MeOH-H_2_O (20–100%, *v*/*v*) to yield three fractions (E2b1–E2b3). Fraction E2b2 (120 mg) was purified by semipreparative RP-HPLC eluted with CH_3_OH–H_2_O (60:40, *v*/*v*) to yield compounds **2** (7.8 mg, *t*_R_ 22.7 min) and **3** (5.4 mg, *t*_R_ 23.5 min). Fraction E3 (2.2 g) was separated on a silica gel CC column and eluted with PE-EtOAc (*v*/*v*, 10:1→2:1) to obtain five fractions (E3a–E3e). Fraction E3c (320 mg) was chromatographed over a Sephadex LH-20 column with MeOH to produce three fractions (E3c1–E3c3). After purification by semipreparative RP-HPLC with CH_3_OH–H_2_O (60:40, *v*/*v*), fraction E3c2 (90 mg) yielded compound **4** (4.8 mg, *t*_R_ 21.5 min). Fraction E4 (900 mg) was separated by a Sephadex LH-20 column with MeOH to yield three major fractions (E4a–E4c). Fraction E4b (85 mg) was purified by semipreparative RP-HPLC with CH_3_OH–H_2_O (62:38, *v*/*v*) to yield compound **7** (21 mg, *t*_R_ 28 min). Fraction F (2.5 g) was chromatographed over a silica gel CC column with PE-EtOAc (*v*/*v*, 10:1→1:1) to yield five fractions (F1–F5). Fraction F2 (220 mg) was subjected to a Sephadex LH-20 column with MeOH to yield three fractions (F2a–F2c). Fraction F2b (45 mg) yielded compound **5** (17 mg, *t*_R_ 25.7 min) after purification by semipreparative RP-HPLC with CH_3_OH–H_2_O (60:40, *v*/*v*). Fraction G (10.5 g) was subjected to a silica gel CC column with PE-EtOAc (*v*/*v*, 10:1→1:1) to yield five fractions (G1–G4). Fraction G2 (1.8 g) was subjected to a reverse-phase C18 silica gel CC column eluted with MeOH-H_2_O (20–90%, *v*/*v*) to obtain three fractions (G2a–G2c). After re-purification using a Sephadex LH-20 column, compound **1** (12 mg, *t*_R_ 19.5 min) was obtained from the fraction G2b (320 mg) through a semipreparative RP-HPLC column with CH_3_OH–H_2_O (65:35, *v*/*v*). Fraction G2c (500 mg) was purified by a Sephadex LH-20 column eluted with MeOH to generate three fractions (G2c1–G2c3). Then, fraction G2c2 (105 mg) was separated by a semipreparative RP-HPLC column with CH_3_OH–H_2_O (60:40, *v*/*v*) to yield compound **6** (30 mg, *t*_R_ 31.3 min). 

### 3.4. Structural Elucidation

Torregrandin A (compound **1**), yellow oil; [α]_D_^25^ +52 (c 0.10, MeOH); UV (MeOH) λ_max_ (log ε) 200 (1.95) nm; IR (KBr) ν_max_ 3427, 3172, 2928, 1717, 1615, 1498, 1399, 1112 cm^−1^; HR-ESI-MS (negative) *m*/*z* 257.1548 [M − H]^−^ (calcd. for C_17_H_21_O_2_, 257.1547); ^1^H and ^13^C NMR dataassigned and listed in Table 1.

Torregrandin B (compound **3**), yellow oil; [α]_D_^25^ +59 (c 0.10, MeOH); UV (MeOH) λ_max_ (log ε) 200 (1.73) nm; IR (KBr) ν_max_ 3604, 3304, 2934, 1714, 1507, 1423, 1368, 1236, 1017 cm^−1^; HR-ESI-MS (negative) *m*/*z* 287.2021 [M − H]^−^ (calcd. for C_19_H_27_O_2_, 287.2017); ^1^H and ^13^C NMR data assigned and listed in Table 2.

### 3.5. Crystal Data for Compounds ***2*** and ***4***

Compound **2**, C_19_H_28_O_2_, Mr = 288.41 block from MeOH, space group *C*_1_2_1_, a = 18.245(3) Å, b = 8.7702 (12) Å, c = 10.5065(15) Å, V = 1591.6 (4) Å^3^, Z = 4, μ = 0.374 mm^−1^, and F(000) = 632.0; T = 170.0; crystal dimensions: 0.08 × 0.06 × 0.04 mm^3^; R = 0.0393, wR = 0.0982, S = 1.090; Flack parameter = 0.17(12); crystallographic data for compound **2** has been deposited at the Cambridge Crystallographic Data Center with the accession number CCDC-2328804.

Compound **4**, C_21_H_28_O_4_, Mr = 344.43 block from MeOH, space group *P*2_1_2_1_2_1_, a = 11.0818(2) Å, b = 11.2194(2) Å, c = 14.5634(2) Å, V = 1810.68(5) Å^3^, Z = 4, μ = 0.690 mm^−1^ and F(000) = 744.0; T = 150.0; crystal dimensions: 0.2 × 0.15× 0.1 mm^3^; R = 0.0713, wR = 0.1545, S = 1.135; Flack parameter = −0.05(5); crystallographic data for compound **4** has been deposited at the Cambridge Crystallographic Data Center with the accession number CCDC-2328805.

### 3.6. ECD Calculation

The ECD calculation was performed according to our previously reported methods [26].

### 3.7. Antibacterial Assay

To determine the MICs, bacterial strains of *Escherichia coli*, methicillin-resistant *Staphylococcus aureus* (MRSA), *Pseudomonas aeruginosa*, and *Salmonella* were cultured in 2 mL of Mueller–Hinton broth (MHB) for 3−5 h at 37 °C, followed by a dilution of the mixture to a concentration of 1 × 10^5^ CFU/mL. Next, 100 μL of each dilution and 100 μL of bacterial suspension were successively added to 96-well plates. Subsequently, the tested compounds were added to give the final concentrations of 200, 100, 50, 25, and 12.5 μM, respectively. The positive groups were rifampicin (10, 5, 2.5, 1.25, 0.625 μM), and the negative and blank groups were 200 μL of media and bacterial solution (1 × 10^5^ CFU/mL). After incubation at 37 °C for 16–18 h, the antibacterial effects were observed by the naked eye. The lowest concentration of the tested compounds which completely inhibited the growth of bacteria was defined as the MIC value. The experiment was repeated at least three times.

### 3.8. Cytotoxicity and Anti-Inflammatory Assays

The bioassays for NO production and cell viability were conducted according to our previously reported methods [27,28]

## 4. Conclusions

In summary, seven abietane-type diterpenoids (compounds **1**–**7**), including three previously undescribed ones and one unreported natural product as well as three known analogs, were isolated and characterized from the arils of *T. grandis*. Moreover, compounds **5** and **6** showed mild inhibitory effects against MRSA, and compounds **1**, **3**, **4**, and **7** exhibited weak anti-neuroinflammatory activity in LPS-induced BV-2 microglia cells. These findings not only enrich the molecular diversity of abietane-type diterpenoids but also offer evidence for antibacterial and anti-neuroinflammatory agents that could be used against human pathogenic bacteria and neuroinflammation-related diseases.

## Figures and Tables

**Table 1:** ^1^H NMR (400 MHz) and ^13^C NMR (100 MHz) spectroscopic data for compounds **1** and **2**.

No.	1 ^a^		2 ^b^	
	*δ*_C_ Type	*δ*_H_ (*J* in Hz)	*δ*_C_ Type	*δ*_H_ (*J* in Hz)
1	38.1	2.24 (m, 1H); 1.77 (m, 1H)	39.5	2.19 (m, 1H); 1.36 (m, 1H)
2	17.8	1.71 (m, 2H)	21.5	1.70 (m, 1H); 1.66 (m, 1H)
3	32.2	1.40 (m, 1H); 1.27 (m, 1H)	43.4	1.80 (m, 1H); 1.43 (m, 1H)
4	49.9		73.2	
5	43.0	1.79 (m, 1H)	53.4	1.55–1.58 (m, 1H)
6	21.4	1.75 (m, 1H); 1.26(m, 1H)	19.3	2.09 (m, 1H); 1.58–1.67 (m, 1H)
7	29.8	2.69 (m, 2H)	30.8	2.78 (m, 2H)
8	136.5		126.8	
9	141.5		148.3	
10	36.2		39.3	
11	125.7	7.05 (d, 8.6, 1H)	111.8	6.63 (s, 1H)
12	113.3	6.56 (dd, 8.6, 3.0, 1H)	153.4	
13	153.4		133.5	
14	115.2	6.43 (d, 3.0, 1H)	127.4	6.75 (s, 1H)
15			27.7	3.17 (m, 1H)
16			23.2	1.16 (d, 7.0, 3H)
17			23.2	1.17 (d, 7.0, 3H)
18	206.6	9.20 (s, 1H)		
19	14.1	1.09 (s, 3H)	22.9	1.19 (s, 3H)
20	25.4	1.13 (s, 3H)	24.9	1.13 (s, 3H)
18-OMe				

^a^ Recorded in CDCl_3_. ^b^ Recorded in CD_3_OD. Chemical shifts (*δ*) are expressed in ppm, and *J* values are expressed in Hz.

**Table 2:** ^1^H NMR (400 MHz) and ^13^C NMR (100 MHz) spectroscopic data for compounds **3** and **4**.

No.	3 ^a^		4 ^b^	
	*δ*_C_ Type	*δ*_H_ (*J* in Hz)	*δ*_C_ Type	*δ*_H_ (*J* in Hz)
1	38.4	2.21 (dt, 13.0, 4.0, 1H); 1.42 (m, 1H)	36.7	2.17 (dd, 12.9, 3.5, 1H); 1.60–1.72 (m, 1H)
2	18.6	1.62 (m, 2H)	17.7	1.60–1.72 (m, 1H); 1.48 (td, 12.9, 3.8, 1H)
3	40.9	1.72 (m, 1H); 1.42 (m, 1H)	36.1	1.76 (t, 12.9, 1H); 1.60–1.72 (m, 1H)
4	72.5		46.2	
5	48.8	1.42 (m, 1H)	44.0	2.45 (dd, 14.1, 3.3, 1H)
6	18.2	2.00 (m, 1H); 1.83 (m, 1H)	37.2	2.68 (dd, 17.6, 14.1, 1H); 1.98 (dd, 17.6, 3.3, 1H)
7	28.8	2.86 (m, 2H)	195.6	
8	127.1		122.3	
9	147.9	2.3 (m, 1H)	155.3	
10	37.3		37.0	
11	110.9	6.64 (s, 1H)	109.3	6.79 (s, 1H)
12	150.9		160.5	
13	131.7		132.9	
14	126.8	6.85 (s, 1H)	125.1	7.64 (s, 1H)
15	27.0	3.12 (m, 1H)	26.1	3.13 (m, 1H)
16	22.7	1.23 (d, 7.0, 3H)	22.2	1.13 (d, 6.4, 3H)
17	22.9	1.24 (d, 7.0, 3H)	22.4	1.14 (d, 6.4, 3H)
18	30.9	1.25 (s, 3H)	177.3	
19			16.2	1.25 (s, 3H)
20	24.5	1.30 (s, 3H)	23.2	1.18 (s, 3H)
18-OMe			52.3	3.59 (s, 3H)

^a^ Recorded in CDCl_3_. ^b^ Recorded in DMSO-*d*_6_. Chemical shifts (*δ*) are expressed in ppm, and *J* values are expressed in Hz.

**Table 3:** Inhibitory effects of compounds **1**, **3**, **4**, and **7** on NO production induced by LPS in BV-2 microglial cells.

Compound	IC_50_ (μM) ^a^	Cell Viability (%) ^b^
**1**	49.4 ± 0.4	81.6 ± 3.5
**3**	41.9 ± 1.5	85.4 ± 1.3
**4**	38.4 ± 0.6	87.1 ± 2.3
**7**	52.6 ± 2.3	96.1 ± 1.0
quercetin ^c^	10.8 ± 1.6	99.7 ± 1.8

^a^ Results are presented as mean ± SD from three independent experiments. ^b^ Cell viability (%) of BV-2 microglial cells at 100 μM expressed as the mean ± SD from three independent experiments. ^c^ Positive control.

## Data Availability

Supporting Information data include HR-ESI-MS, UV, CD, and 1D and 2D NMR spectra.

## Supplementary Materials

The following supporting information can be downloaded at: https://www.mdpi.com/article/10.3390/molecules29091905/s1, Figures S1–S7: The 1D and 2D NMR spectra of compound **1** in CDCl_3_, Figure S8: The HR-ESI-MS spectrum of compound **1**, Figures S9 and S10: The IR and UV spectra of compound **1**, Figures S11–S16: The 1D and 2D NMR spectra of compound **2** in CD_3_OD, Figure S17: The HR-ESI-MS spectrum of compound **2**, Figures S18 and S19: The IR and UV spectra of compound **2**, Figures S20–S26: The 1D and 2D NMR spectra of compound **3** in CDCl_3_, Figure S27: The HR-ESI-MS spectrum of compound **3**, Figures S28 and S29: The IR and UV spectra of compound **3**, Figures S30–S32: The 1D NMR spectra of compound **4** in DMSO-*d*_6_, Figure S33: The HR-ESI-MS spectrum of compound **4**, Figures S34 and S35: The IR and UV spectra of compound **4**, Figures S36 and S37: The 1D NMR spectra of compound **5** in CDCl_3_, Figures S38 and S39: The 1D NMR spectra of compound **6** in CDCl_3_, Figures S40 and S41: The 1D NMR spectra of compound **7** in CDCl_3_. Table S1: Conformational analysis of the B3LYP/6-31G(d) optimized conformers of **1** in the gas phase, Tables S2 and S3: Atomic coordinates (Å) of 2 conformers of **1** obtained at the B3LYP/6-31G(d) level of theory in the gas phase, Tables S4 and S5: Key transitions, oscillator strengths, and rotatory strengths in the ECD spectra of 2 conformers of **1** at the CAM-B3LYP/6-311G(d) level of theory in MeOH with IEFPCM solvent model, Table S6: Conformational analysis of the B3LYP/6-31G(d) optimized conformers of **3** in the gas phase, Tables S7–S13: Atomic coordinates (Å) of 7 conformers of **3** obtained at the B3LYP/6-31G(d) level of theory in the gas phase, Tables S14–S20: Key transitions, oscillator strengths, and rotatory strengths in the ECD spectra of 7 conformers of **3** at the CAM-B3LYP/6-311G(d) level of theory in MeOH with IEFPCM solvent model.

## References

[1] Majeed A., Singh A., Choudhary S., Bhardwaj P. (2019). RNAseq-based phylogenetic reconstruction of Taxaceae and Cephalotaxaceae. Cladistics.

[2] Zhou W., Harris A.J., Xiang Q.Y. (2022). Phylogenomics and biogeography of *Torreya* (Taxaceae)—Integrating data from three organelle genomes, morphology, and fossils and a practical method for reducing missing data from RAD-seq. J. Syst. Evol..

[3] Miao Z.P., Niu X.N., Wang R.B., Huang L., Ma B.B., Li J.H., Hong X. (2022). Study of the genus *Torreya* (Taxaceae) based on chloroplast genomes. Front. Biosci..

[4] Shi L.K., Mao J.H., Zheng L., Zhao C.W., Jin Q.Z., Wang X.G. (2018). Chemical characterization and free radical scavenging capacity of oils obtained from *Torreya grandis* Fort. ex. Lindl. and *Torreya grandis* Fort. var. Merrillii: A comparative study using chemometrics. Ind. Crops Prod..

[5] Song L., Meng X., Song H., Gao L., Gao Y., Chen W., Huan W., Suo J., Yu W., Wang X.H. (2023). Bioactive ellagitannins and phenylpropanoid glycosides from the seed of *Torreya grandis*. Phytochem. Lett..

[6] Cui J.J., Li W.J., Wang C.L., Huang Y.Q., Lin W., Zhou B., Yue J.M. (2022). Antimicrobial abietane-type diterpenoids from *Torreya grandis*. Phytochemistry.

[7] Beatrice G., Francesco G., Virginia L., Domenico M., Raffaele R., Claudio V., He G.F., Ma Z.W., Yin W.F. (1999). Grandione, a new heptacyclic dimeric diterpene from *Torreya grandis* Fort. Tetrahedron.

[8] Saeed M.K., Khan M.N., Ahmad I., Hussain N., Ali S., Deng Y., Dai R. (2013). Isolation, identification and antioxidant potential of major flavonoids from ethyl acetate fraction of *Torreya grandis*. Asian J. Chem..

[9] Shi H., Wang H., Wang M., Li X. (2009). Antioxidant activity and chemical composition of *Torreya grandis* cv. *Merrillii* seed. Nat. Prod. Commun..

[10] He Z., Zhu H., Li W., Zeng M., Wu S., Chen S., Qin F., Chen J. (2016). Chemical components of cold pressed kernel oils from different *Torreya grandis* cultivars. Food Chem..

[11] Saeed M.K., Deng Y., Dai R., Li W., Yu Y., Iqbal Z. (2010). Appraisal of antinociceptive and anti-inflammatory potential of extract and fractions from the leaves of *Torreya grandis* Fort Ex. Lindl. J. Ethnopharmacol..

[12] Ma J., Yuan T., Gao Y., Zeng X., Liu Z., Gao J. (2023). *Torreya grandis* oil attenuates cognitive impairment in scopolamine-induced mice. Food Funct..

[13] Yu Y.J., Ni S., Wu F., Sang W.G. (2016). Chemical composition and antioxidant activity of essential oil from *Torreya grandis* cv. *merrillii* Arils. J. Essent. Oil Bear. Plants.

[14] Zhou D.Z., Yi Y.H., Mao S.L., Lu T.S., Tang H.F., Zou Z.R., Zhang S.Y. (2004). The lignins from *Torreya grandis* cv. *Merrilli*. Acta Pharm. Sin..

[15] Tang J.J., Huang L.F., Deng J.L., Wang Y.M., Guo C., Peng X.N., Liu Z., Gao J.M. (2022). Cognitive enhancement and neuroprotective effects of OABL, a sesquiterpene lactone in 5xFAD Alzheimer’s disease mice model. Redox Biol..

[16] Xie J.Y., Wang Z.X., Liu W.Y., Liu H.W., Li D., Sang Y.F., Yang Z., Gao J.M., Yan X.T. (2023). Hyperelatolides A–D, antineuroinflammatory constituents with unusual carbon skeletons from *Hypericum elatoides*. J. Nat. Prod..

[17] Xie J.Y., Li P., Yan X.T., Gao J.M. (2024). Discovery from *Hypericum elatoides* and synthesis of hyperelanitriles as α-aminopropionitrile-containing polycyclic polyprenylated acylphloroglucinols. Commun. Chem..

[18] Cheung H.T., Miyase T., Lenguyen M.P., Smal M.A. (1993). Further acidic constituents and neutral components of *Pinus massoniana* Resin. Tetrahedron.

[19] Harrison L.J., Asakawa Y. (1987). 18-Oxoferruginol from the leaf of *Torreya nucifera*. Phytochemistry.

[20] Yang Z., Luo W., Yang Z., Zhang M., Dong M., Guo D., Gu J., Sun C., Xiao S. (2024). Diterpenoids from *Torreya grandis* and their cytotoxic activities. Phytochemistry.

[21] Yang X.W., Li S.M., Feng L., Shen Y.H., Tian J.M., Liu X.H., Zeng H.W., Zhang C., Zhang W.D. (2008). Abiesanordines A–N: Fourteen new norditerpenes from *Abies georgei*. Tetrahedron.

[22] Hamulić D., Stadler M., Hering S., Padrón J.M., Bassett R., Rivas F., Loza-Mejía M.A., Dea-Ayuela M.A., González-Cardenete M.A. (2019). Synthesis and biological studies of (+)-liquiditerpenoic acid A (abietopinoic acid) and representative analogues: SAR studies. J. Nat. Prod..

[23] Fraga B.M., Hernández M.G., Artega J.M., Suárez S. (2003). The microbiological transformation of the diterpenes dehydroabietanol and teideadiol by *Mucor plumbeus*. Phytochemistry.

[24] van Beek T.A., Claassen F.W., Dorado J., Godejohann M., Sierra-Alvarez R., Wijnberg J.B. (2007). Fungal biotransformation products of dehydroabietic acid. J. Nat. Prod..

[25] He G., Ma Z., Yin W. (1985). A new diterpenoid component torreyagrandate from leaves of *Torreya grandis* Fort. endemic in China. Acta Bot. Sin..

[26] Zhai L.L., Jiang T.T., Zhang R., Li J.N., Zhai Y.J., Zhang Q., Li D., Han W.B. (2023). Ergostane-type sterols and sesquiterpenes with anti-neuroinflammatory activity from a *Nigrograna* species associated with *Clematis shensiensis*. Phytochemistry.

[27] Tang D., Liu L.L., He Q.R., Yan W., Li D., Gao J.M. (2018). Ansamycins with antiproliferative and antineuroinflammatory activity from moss-soil-derived *Streptomyces cacaoi* subsp. *asoensis* H2S5. J. Nat. Prod..

[28] Han W.B., Wang G.Y., Tang J.J., Wang W.J., Liu H., Gil R.R., Navarro-Vázquez A., Lei X., Gao J.M. (2020). Herpotrichones A and B, two Intermolecular [4 + 2] adducts with anti-neuroinflammatory activity from a *Herpotrichia* Species. Org. Lett..

