# Supplementary material for: Abietane-Type Diterpenoids from the Arils of Torreya grandis
Source: Molecules. 2024 Apr 23;29(9):1905. doi: 10.3390/molecules29091905 (PMC11085307; doi:10.3390/molecules29091905)
Supplement: Supplementary file 1 [file molecules-29-01905-s001.zip › molecules-2957107-supplementary.pdf]

Abietane-type diterpenoids from the arils of *Torreya grandis*

Yuqi Gao [1], Jinghui Yang [2], Yue Zhang [2], Linlin Gao [2], Junmian Tian [2,*],

Wenbo Han [2,*] and Jinming Gao [2]

[1]College of Food Science and Technology, Northwest University, Xi'an 710075, China; gaoyuqi1989@gmail.com

[2]Shaanxi Key Laboratory of Natural Products & Chemical Biology, College of Chemistry & Pharmacy, Northwest A&F University, Yangling 712100, China; 2020056692@nwafu.edu.cn (J.Y.); zhangyuey@nwafu.edu.cn (Y.Z.); linlingaoluck@163.com (L.G.); jinminggao@nwsuaf.edu.cn (J.G.)

* Correspondence: tianjunmian@nwsuaf.edu.cn (J.T.); wbhan@nwafu.edu.cn (W.H.)

Table of contents

Figure S1. The ^{1}H NMR spectrum of 1 in $CDCl_3$ (400 MHz).......5

Figure S2. The ^{13}C NMR spectrum of 1 in $CDCl_3$ (100 MHz).......5

Figure S3. The DEPT135 spectrum of 1 in $CDCl_3$ (100 MHz).......6

Figure S4. The ^{1}H-^{1}H COSY spectrum of 1 in $CDCl_3$.......6

Figure S5. The HSQC spectrum of 1 in $CDCl_3$.......7

Figure S6. The HMBC spectrum of 1 in $CDCl_3$.......7

Figure S7. The NOESY spectrum of 1 in $CDCl_3$. 8

Figure S8. The HRESIMS spectrum of 1.......8

Figure S9. The IR spectrum of 1.......9

Figure S10. The UV spectrum of 1. 9

Figure S11. The ^{1}H NMR spectrum of 2 in CD_3OD (400 MHz).......10

Figure S12. The ^{13}C NMR spectrum of 2 in CD_3OD (100 MHz).......10

Figure S13. The DEPT135 spectrum of 2 in CD_3OD (100 MHz).......11

Figure S14. The ^{1}H-^{1}H COSY spectrum of 2 in CD_3OD.......11

Figure S15. The HSQC spectrum of 2 in CD3OD.......11

Figure S16. The HMBC spectrum of 2 in CD_3OD. 12

Figure S17. The HRESIMS spectrum of 2.......13

Figure S18. The IR spectrum of 2.......13

Figure S19. The UV spectrum of 2. 14

Figure S20. The ^{1}H NMR spectrum of 3 in $CDCl_3$ (400 MHz).......14

Figure S21. The ^{13}C NMR spectrum of 3 in $CDCl_3$ (100 MHz).......15

Figure S22. The DEPT135 spectrum of 3 in $CDCl_3$ (100 MHz).......11

Figure S23. The ^{1}H-^{1}H COSY spectrum of 3 in $CDCl_3$.......16

Figure S24. The HSQC spectrum of 3 in $CDCl_3$.......16

Figure S25. The HMBC spectrum of 3 in $CDCl_3$.......17

Figure S26. The NOESY spectrum of 3 in $CDCl_3$. 17

Figure S27. The HRESIMS spectrum of 3.......18

Figure S28. The IR spectrum of 3.......18

Figure S29. The UV spectrum of 3. 19

Figure S30. The ^{1}H NMR spectrum of 4 in DMSO-d_6 (400 MHz).......19

Figure S31. The ^{13}C NMR spectrum of 4 in DMSO-d_6 (100 MHz).......20

Figure S32. The DEPT135 spectrum of 4 in DMSO-d_6 (100 MHz). 20

Figure S33. The HRESIMS spectrum of 4.......21

Figure S34. The IR spectrum of 4.......21

Figure S35. The UV spectrum of 4. 22

Figure S36. The ^{1}H NMR spectrum of 5 in $CDCl_3$ (400MHz).......22

Figure S37. The ^{13}C NMR spectrum of 5 in $CDCl_3$ (100MHz).......23

Figure S38. The ^{1}H NMR spectrum of 6 in $CDCl_3$ (400MHz).......23

Figure S39. The ^{13}C NMR spectrum of 6 in $CDCl_3$ (100MHz).......24

Figure S40. The ^{1}H NMR spectrum of 7 in $CDCl_3$ (400MHz).. 24
Figure S41. The ^{13}C NMR spectrum of 7 in $CDCl_3$ (100MHz).. 25
Table S1. Conformational analysis of the B3LYP/6-31G(d) optimized conformers of 1 in the gas phase (T=298.15 K).. 25
Table S2. Atomic coordinates (Å) of 1-1 obtained at the B3LYP/6-31G(d) level of theory in the gas phase.. 25
Table S3. Atomic coordinates (Å) of 1-2 obtained at the B3LYP/6-31G(d) level of theory in the gas phase.. 26
Table S4. Key transitions, oscillator strengths, and rotatory strengths in the ECD spectrum of conformer 1-1 at the CAM-B3LYP/6-311G(d) level of theory in MeOH with IEFPCM solvent model... 27
Table S5. Key transitions, oscillator strengths, and rotatory strengths in the ECD spectrum of conformer 1-2 at the CAM-B3LYP/6-311G(d) level of theory in MeOH with IEFPCM solvent model... 29
Table S6. Conformational analysis of the B3LYP/6-31G(d) optimized conformers of 3 in the gas phase (T=298.15 K).. 31
Table S7. Atomic coordinates (Å) of 3-1 obtained at the B3LYP/6-31G(d) level of theory in the gas phase.. 32
Table S8. Atomic coordinates (Å) of 3-2 obtained at the B3LYP/6-31G(d) level of theory in the gas phase.. 32
Table S9. Atomic coordinates (Å) of 3-3 obtained at the B3LYP/6-31G(d) level of theory in the gas phase.. 33
Table S10. Atomic coordinates (Å) of 3-4 obtained at the B3LYP/6-31G(d) level of theory in the gas phase.. 34
Table S11. Atomic coordinates (Å) of 3-5 obtained at the B3LYP/6-31G(d) level of theory in the gas phase.. 35
Table S12. Atomic coordinates (Å) of 3-6 obtained at the B3LYP/6-31G(d) level of theory in the gas phase.. 35
Table S13. Atomic coordinates (Å) of 3-7 obtained at the B3LYP/6-31G(d) level of theory in the gas phase.. 36
Table S14. Key transitions, oscillator strengths, and rotatory strengths in the ECD spectrum of conformer 3-1 at the CAM-B3LYP/6-311G(d) level of theory in MeOH with IEFPCM solvent model... 37
Table S15. Key transitions, oscillator strengths, and rotatory strengths in the ECD spectrum of conformer 3-2 at the CAM-B3LYP/6-311G(d) level of theory in MeOH with IEFPCM solvent model... 40
Table S16. Key transitions, oscillator strengths, and rotatory strengths in the ECD spectrum of conformer 3-3 at the CAM-B3LYP/6-311G(d) level of theory in MeOH with IEFPCM solvent model... 42

Table S17. Key transitions, oscillator strengths, and rotatory strengths in the ECD spectrum of conformer 3-4 at the CAM-B3LYP/6-311G(d) level of theory in MeOH with IEFPCM solvent model. **45**
Table S18. Key transitions, oscillator strengths, and rotatory strengths in the ECD spectrum of conformer 3-5 at the CAM-B3LYP/6-311G(d) level of theory in MeOH with IEFPCM solvent model. **47**
Table S19. Key transitions, oscillator strengths, and rotatory strengths in the ECD spectrum of conformer 3-6 at the CAM-B3LYP/6-311G(d) level of theory in MeOH with IEFPCM solvent model. **50**
Table S20. Key transitions, oscillator strengths, and rotatory strengths in the ECD spectrum of conformer 3-7 at the CAM-B3LYP/6-311G(d) level of theory in MeOH with IEFPCM solvent model. **53**

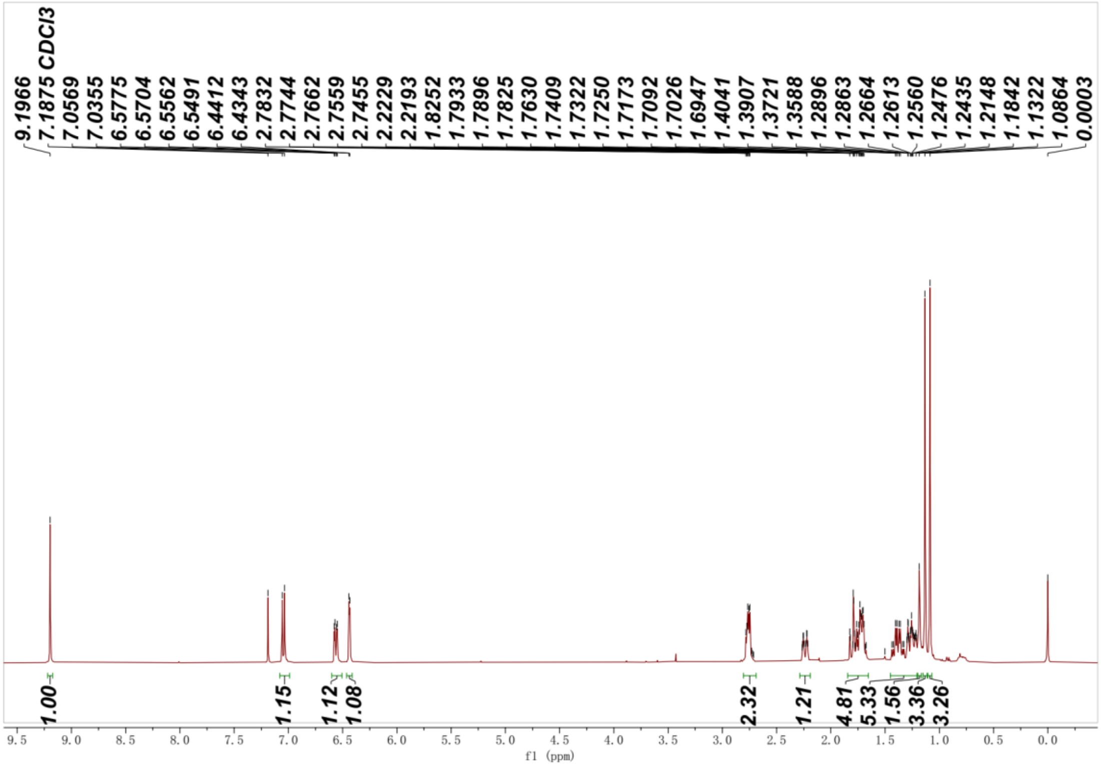

Figure S1. The ^{1}H NMR spectrum of **1** in $CDCl_3$ (400 MHz).

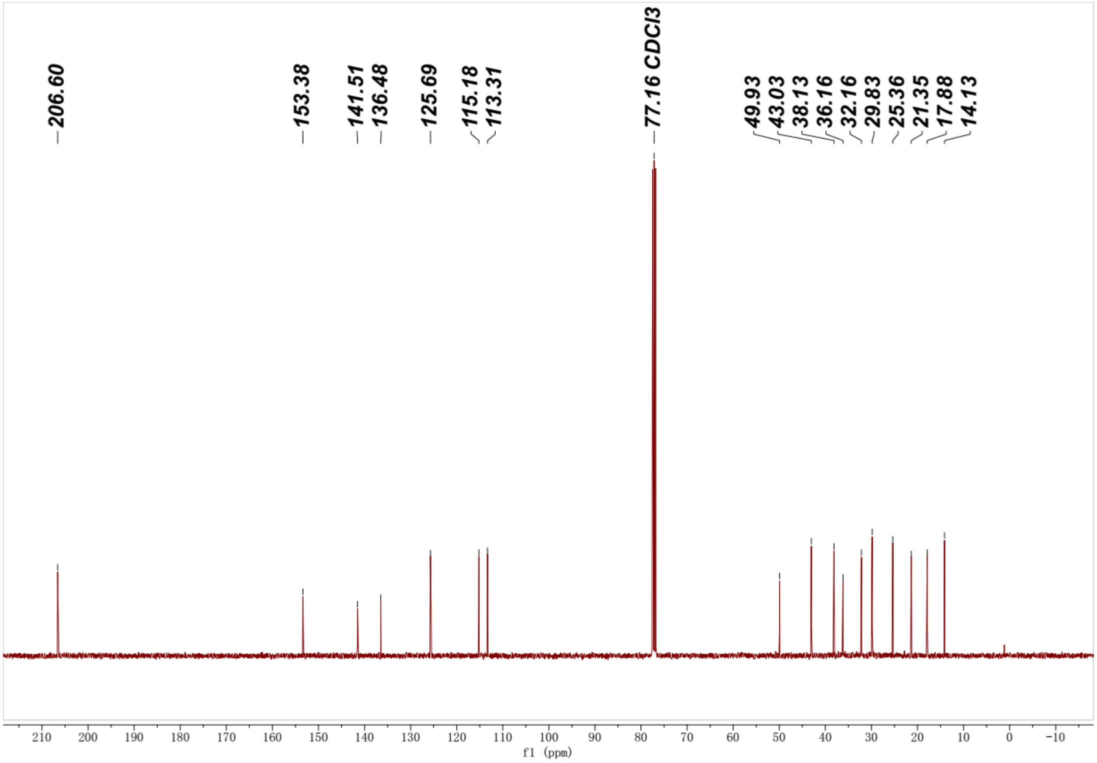

Figure S2. The ^{13}C NMR spectrum of **1** in $CDCl_3$ (100 MHz).

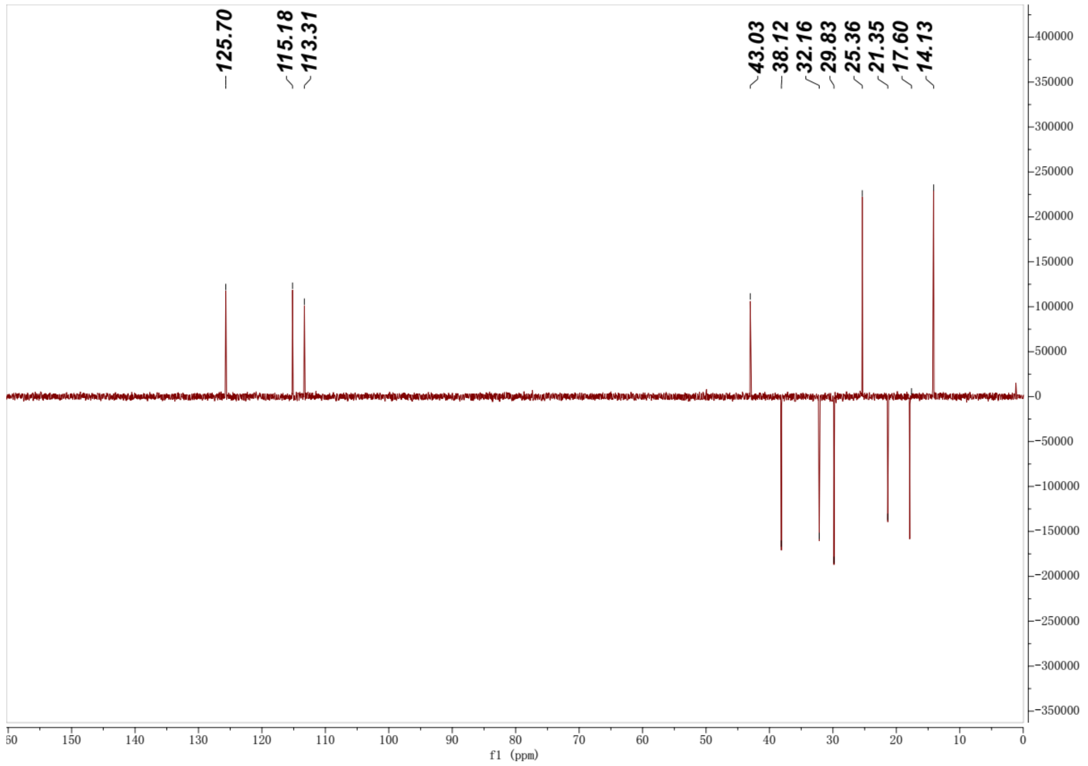

Figure S3. The DEPT135 spectrum of **1** in $CDCl_3$ (100 MHz).

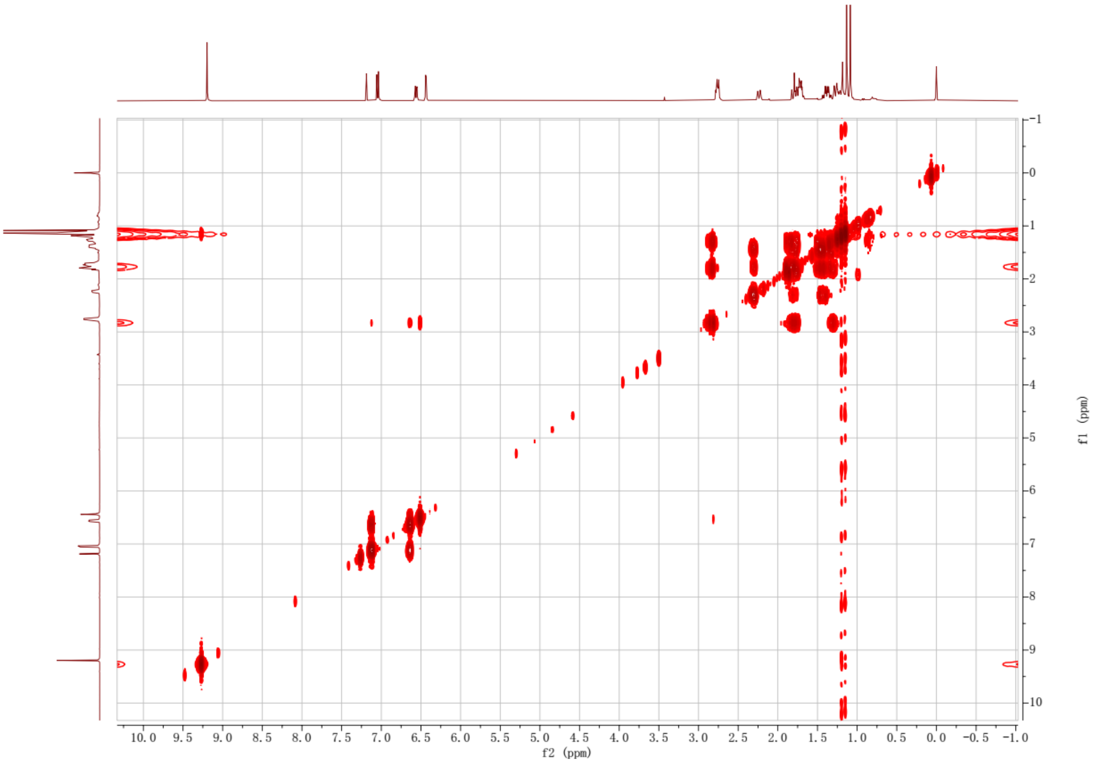

Figure S4. The ^{1}H-^{1}H COSY spectrum of **1** in $CDCl_3$.

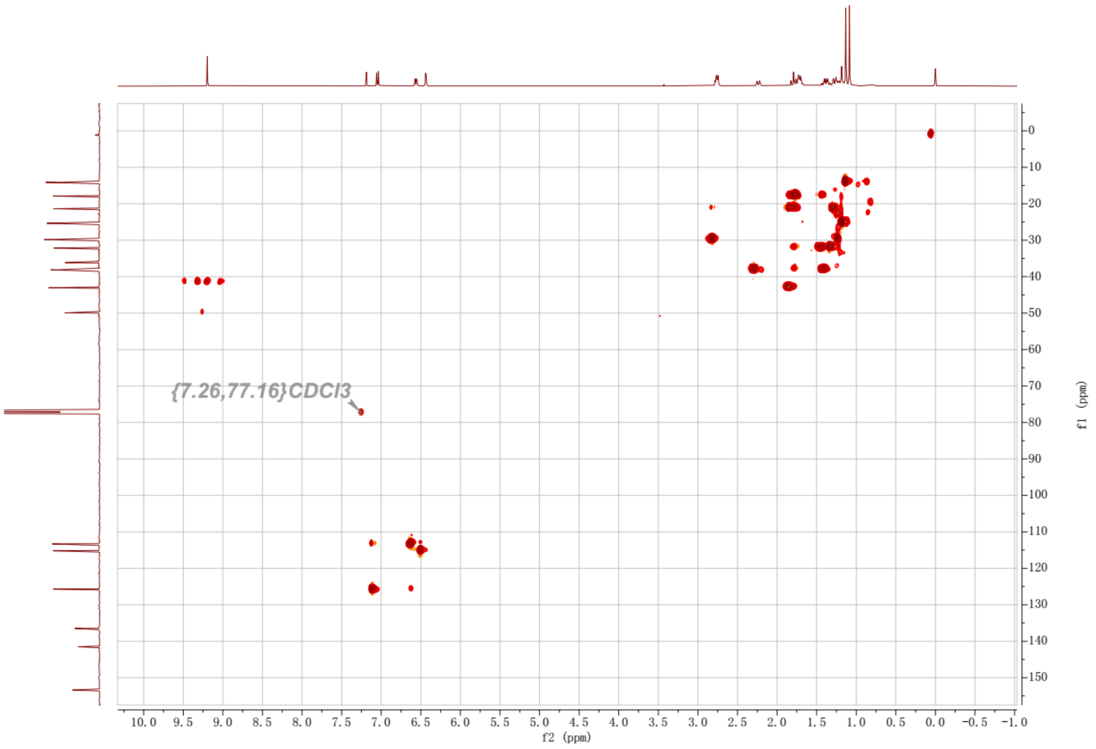

Figure S5. The HSQC spectrum of **1** in $CDCl_3$.

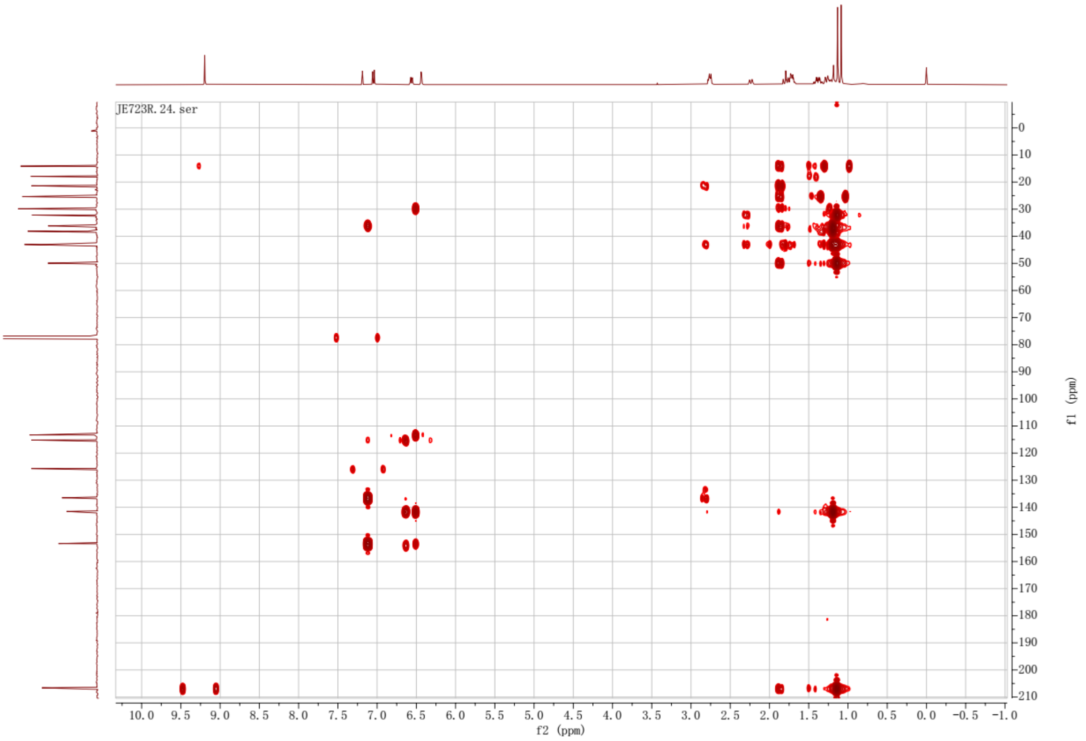

Figure S6. The HMBC spectrum of **1** in $CDCl_3$.

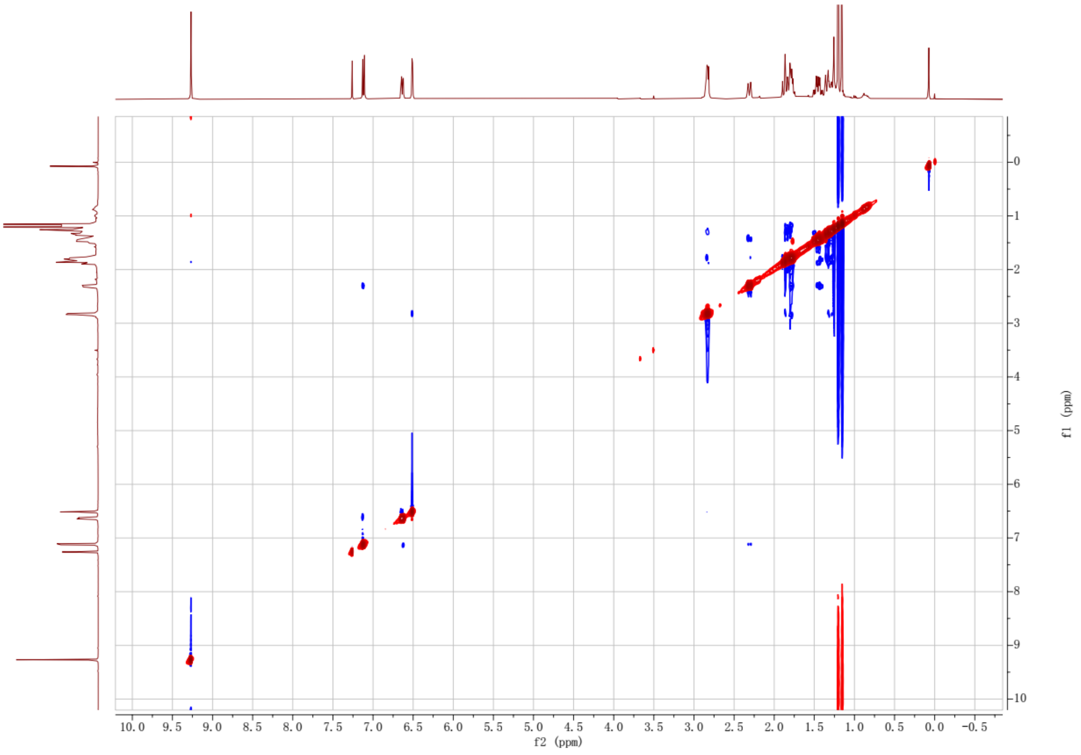

Figure S7. The NOESY spectrum of **1** in $CDCl_3$.

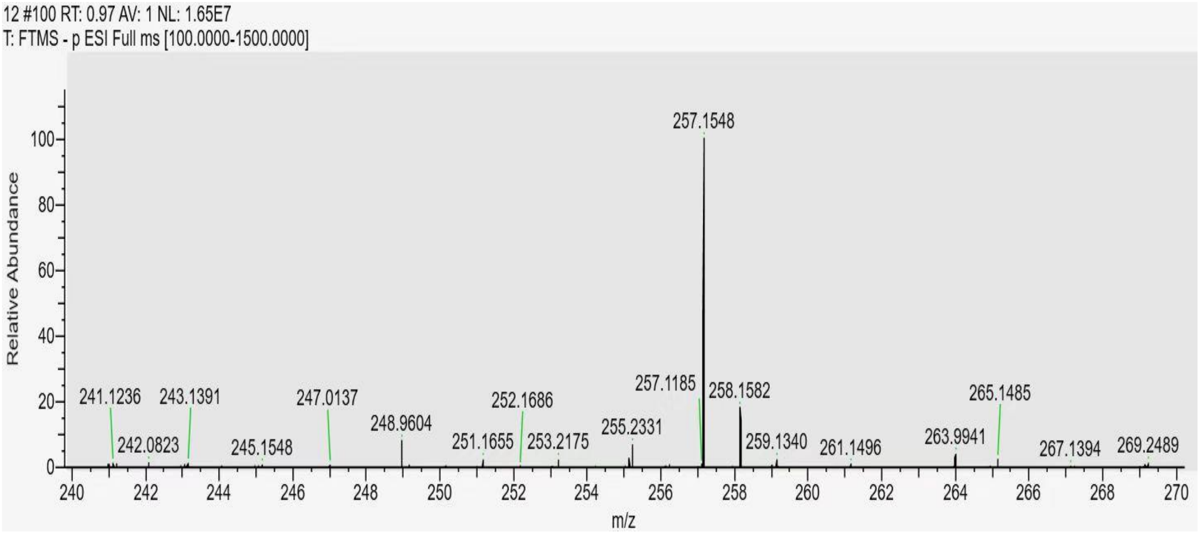

Figure S8. The HRESIMS spectrum of **1**.

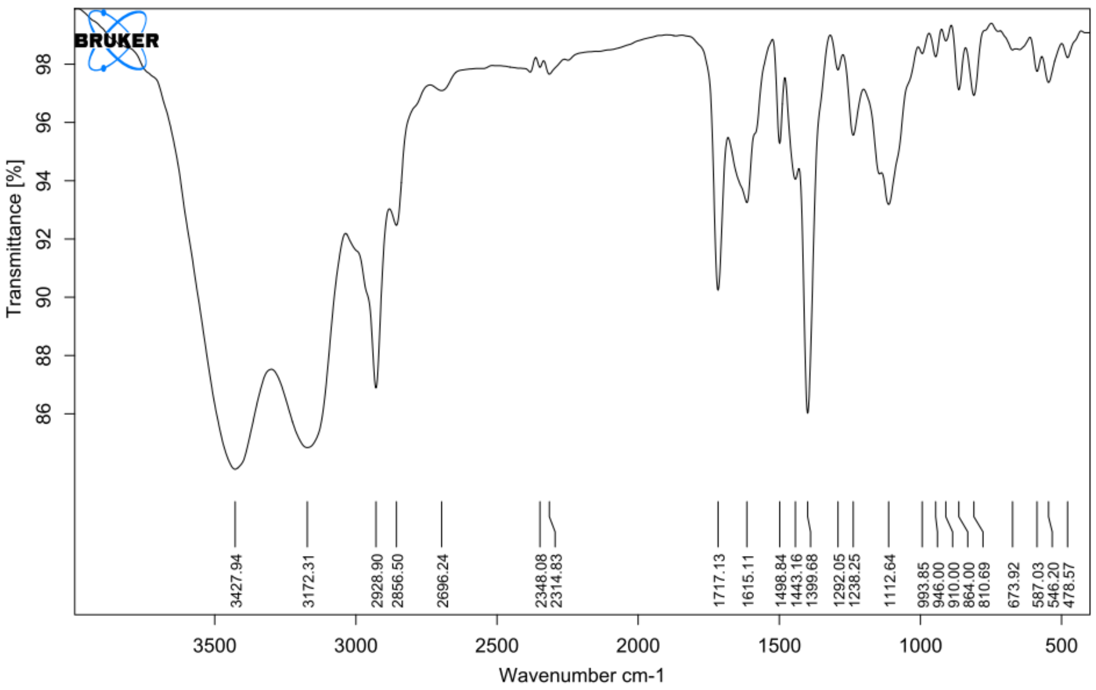

Figure S9. The IR spectrum of **1**.

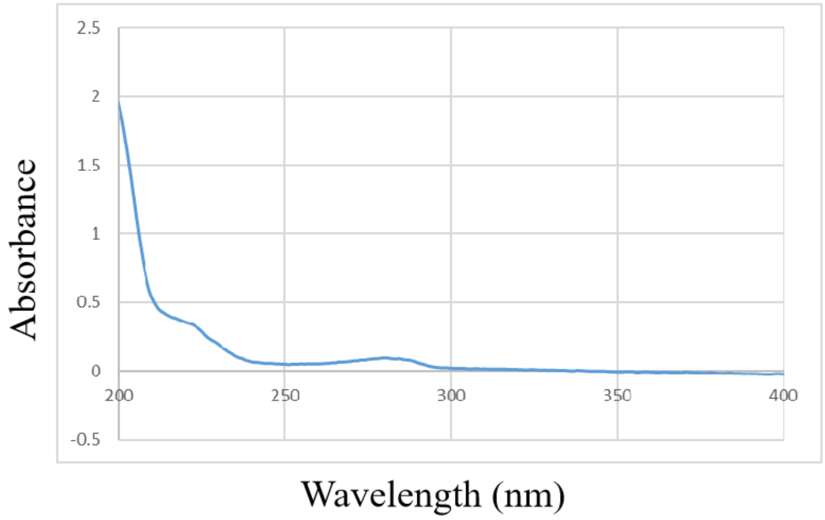

Figure S10. The UV spectrum of **1**.

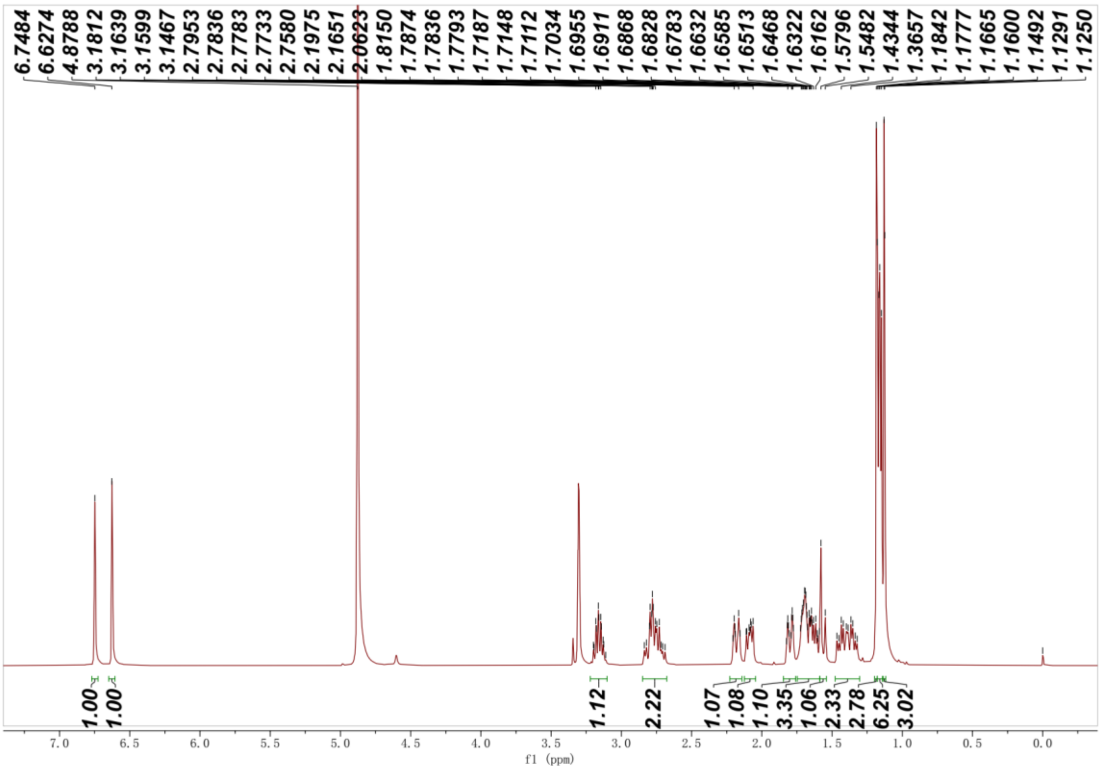

Figure S11. The ^{1}H NMR spectrum of **2** in CD_3OD (400 MHz).

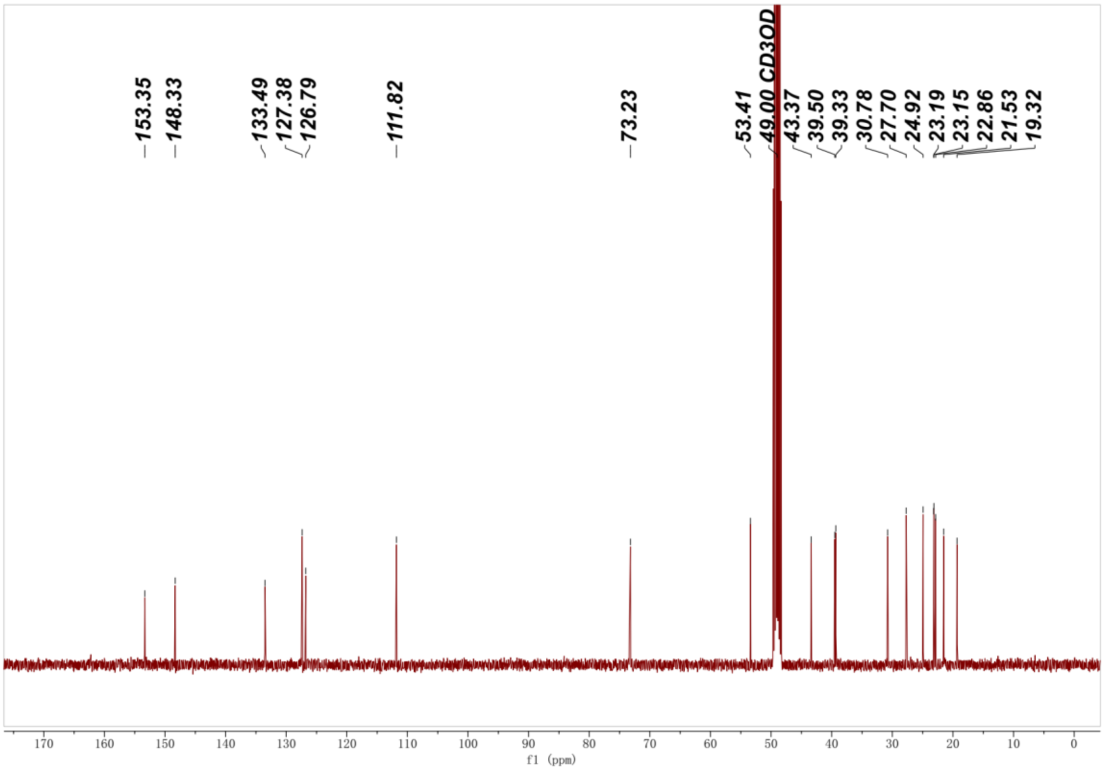

Figure S12. The ^{13}C NMR spectrum of **2** in CD_3OD (100 MHz).

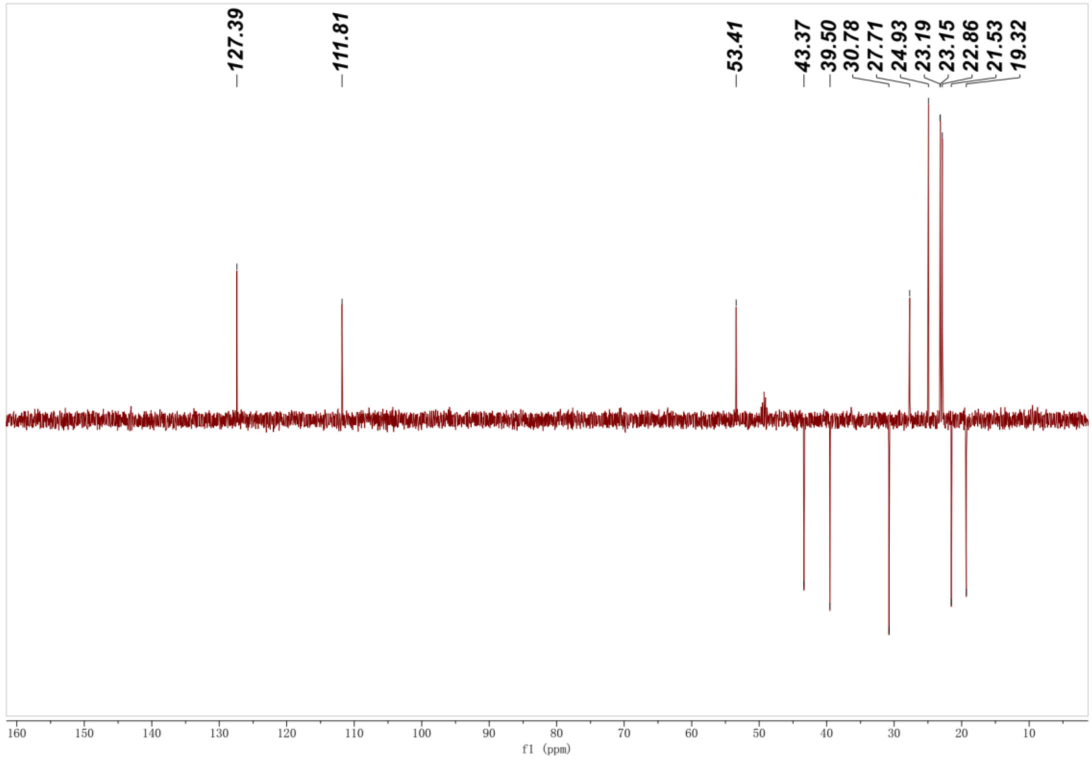

Figure S13. The DEPT135 spectrum of **2** in CD_3OD (100 MHz).

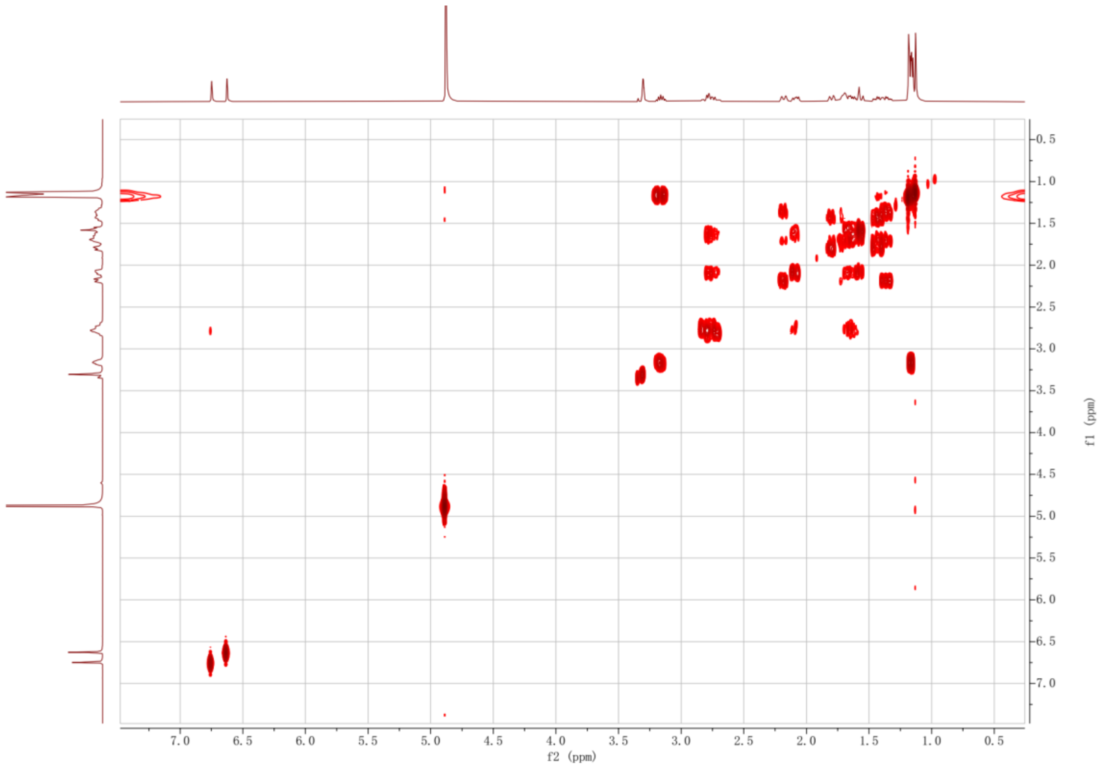

Figure S14. The ^1H-^1H COSY spectrum of **2** in CD_3OD.

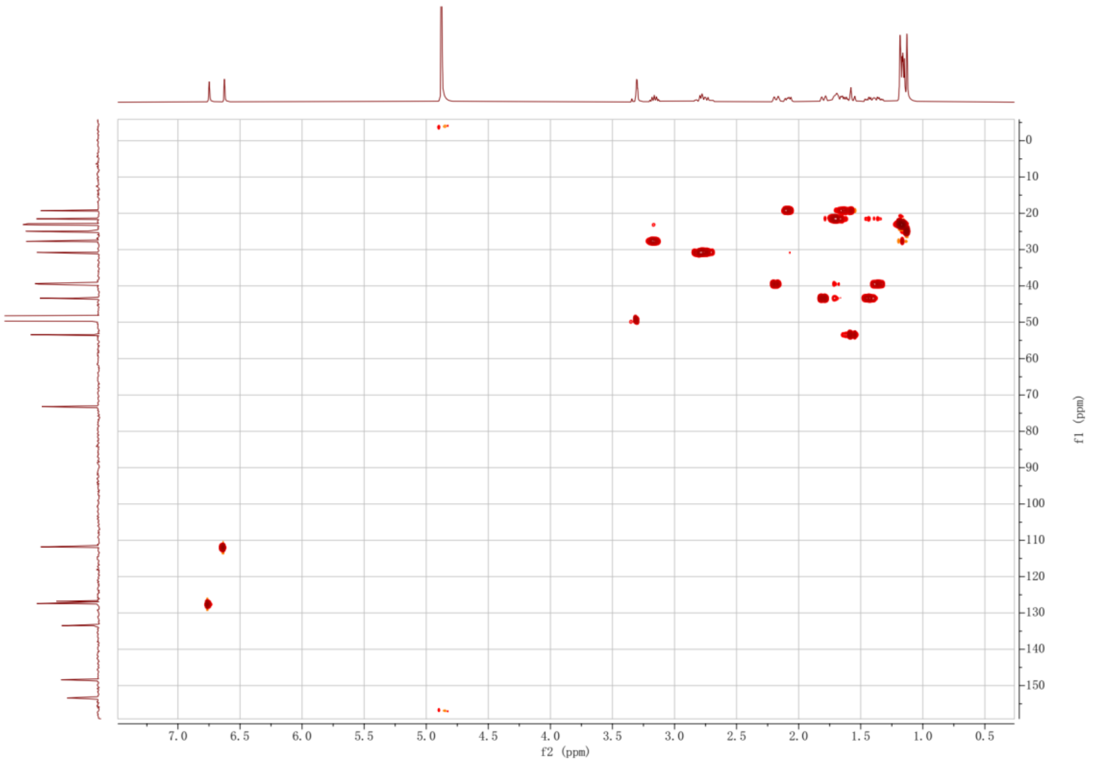

Figure S15. The HSQC spectrum of 2 in CD_3OD.

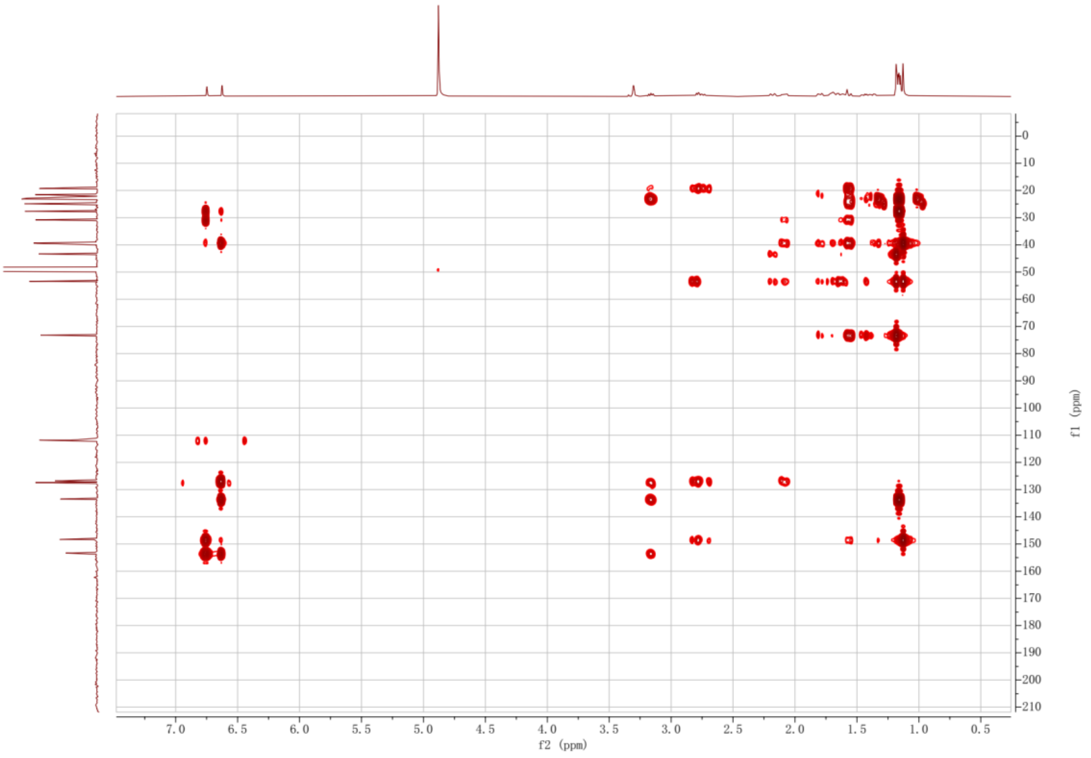

Figure S16. The HMBC spectrum of **2** in CD_3OD.

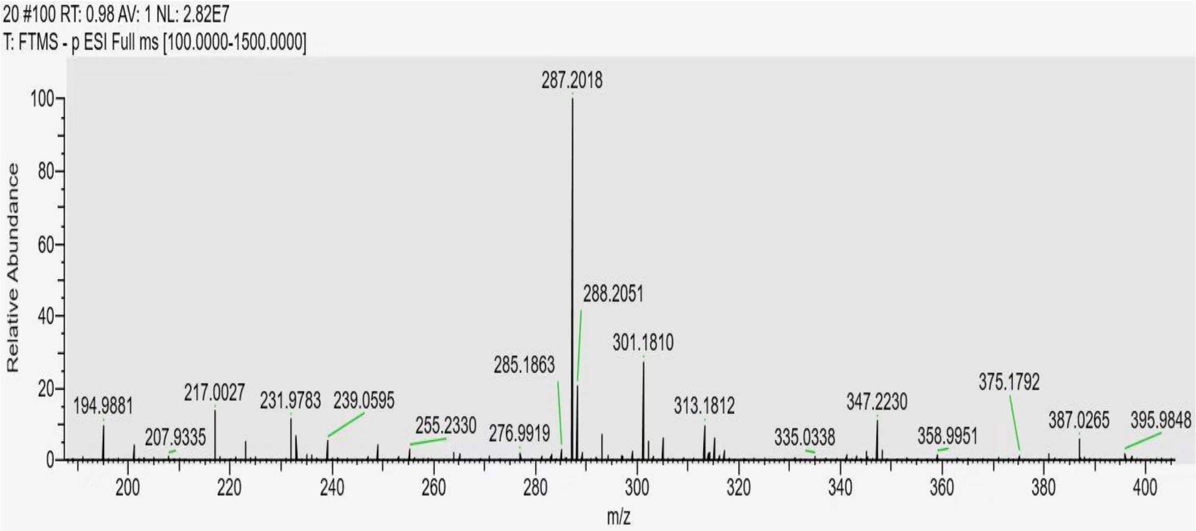

Figure S17. The HRESIMS spectrum of **2**.

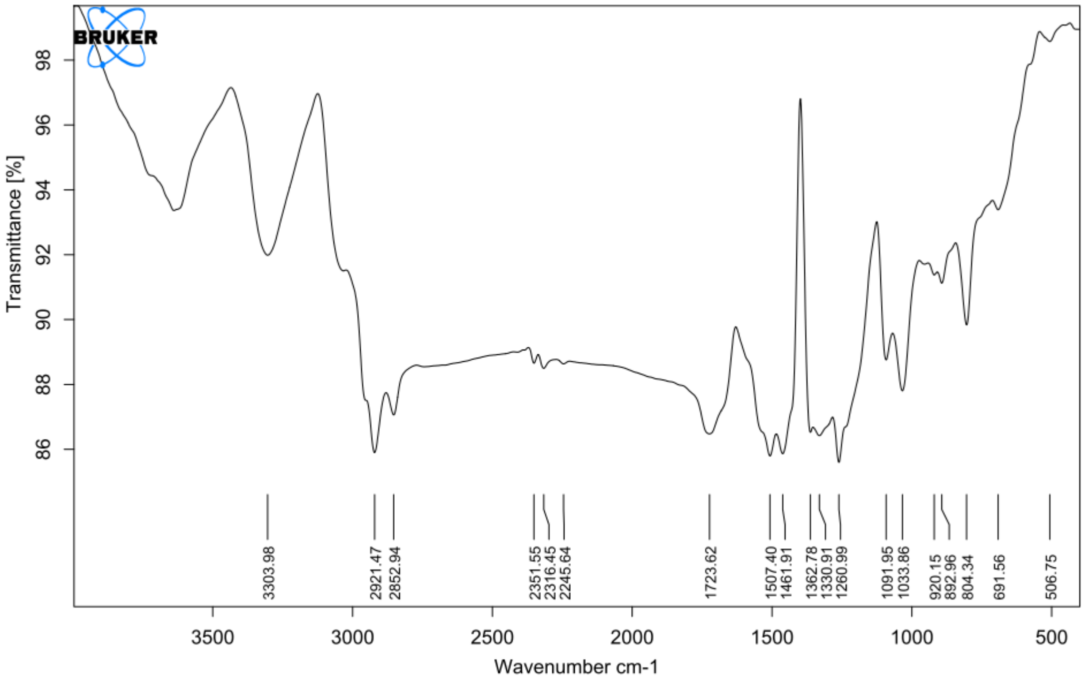

Figure S18. The IR spectrum of **2**.

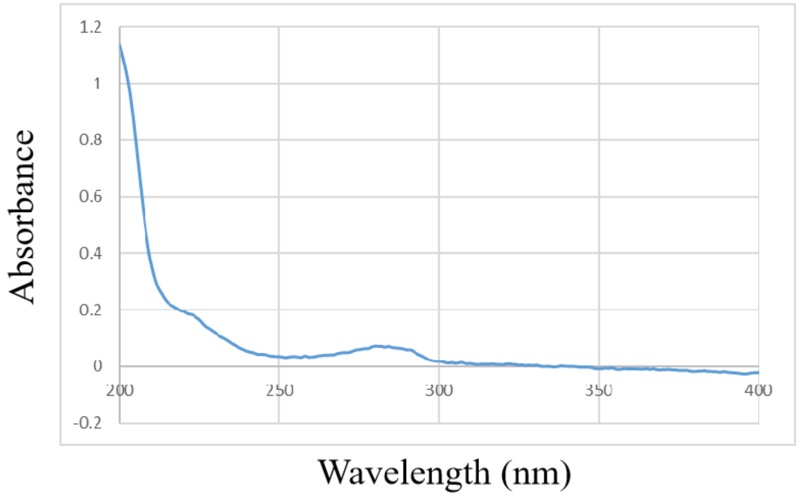

Figure S19. The UV spectrum of **2**.

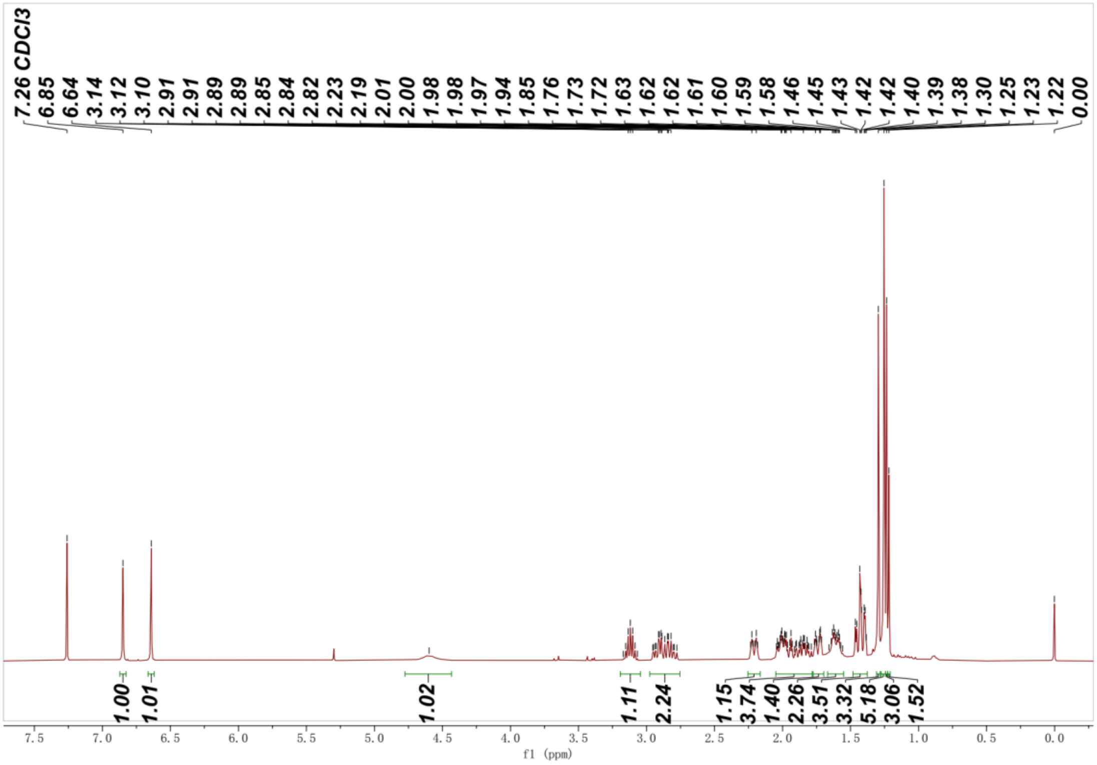

Figure S20. The ^{1}H NMR spectrum of **3** in $CDCl_3$ (400 MHz).

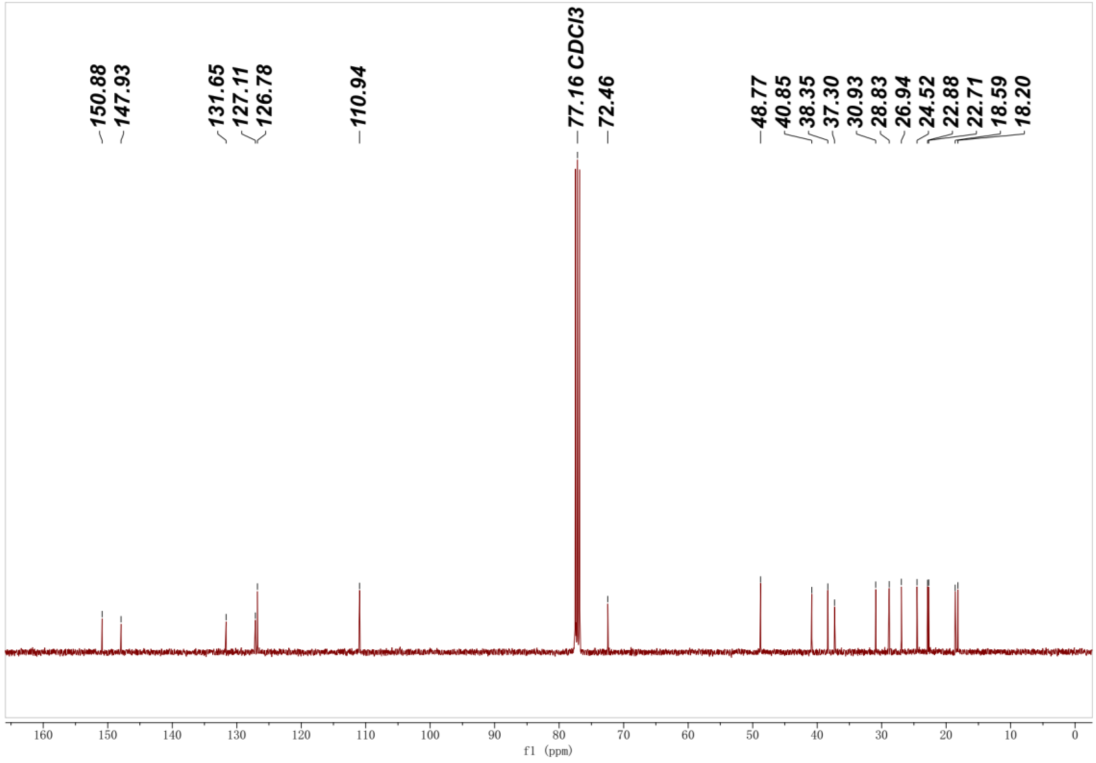

Figure S21. The ^{13}C NMR spectrum of **3** in $CDCl_3$ (100 MHz).

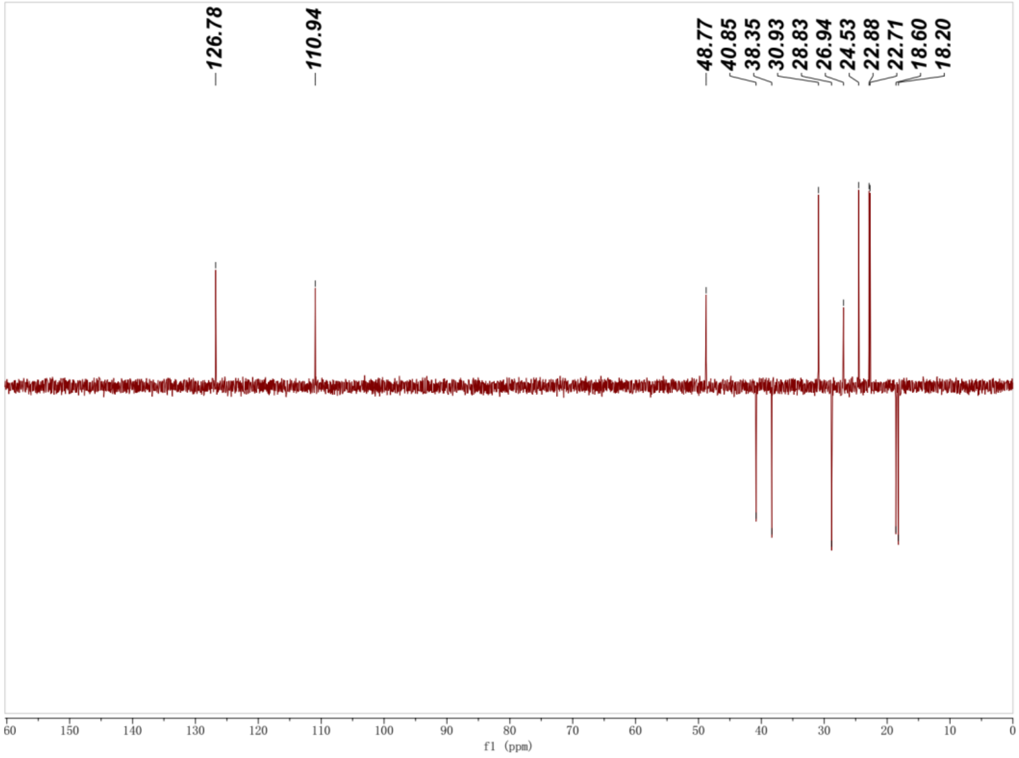

Figure S22. The DEPT135 spectrum of **3** in $CDCl_3$ (100 MHz).

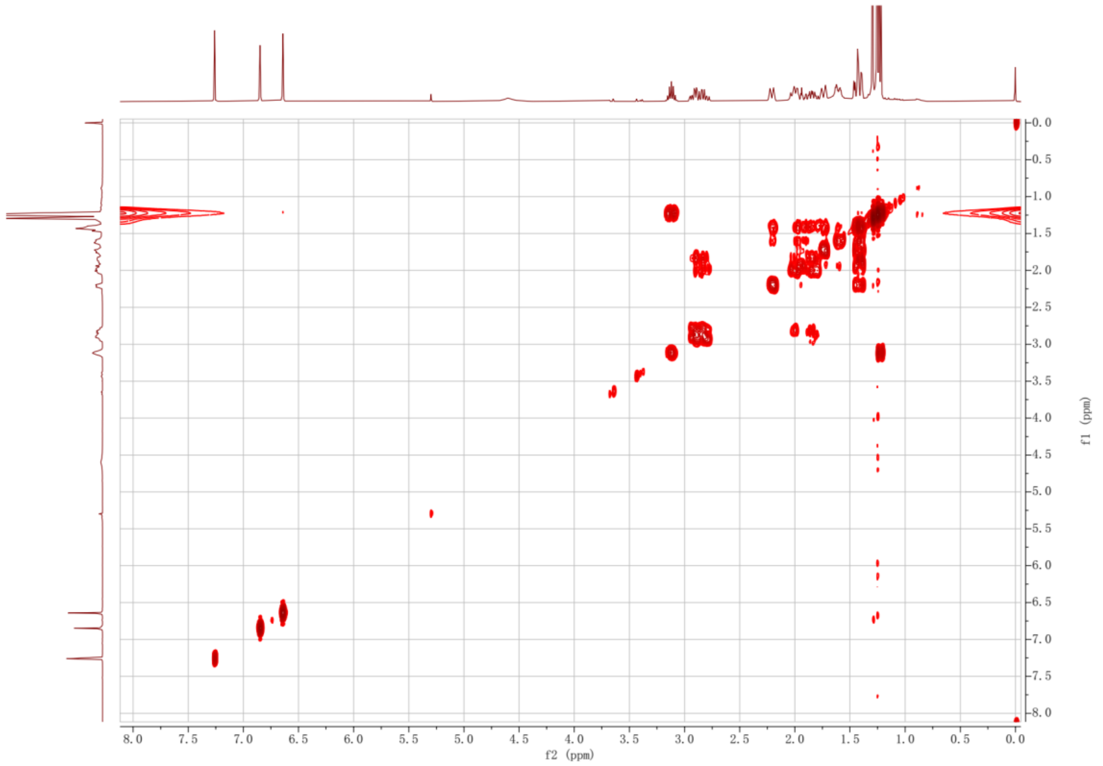

Figure S23. The ^1H-^1H COSY spectrum of **3** in $CDCl_3$.

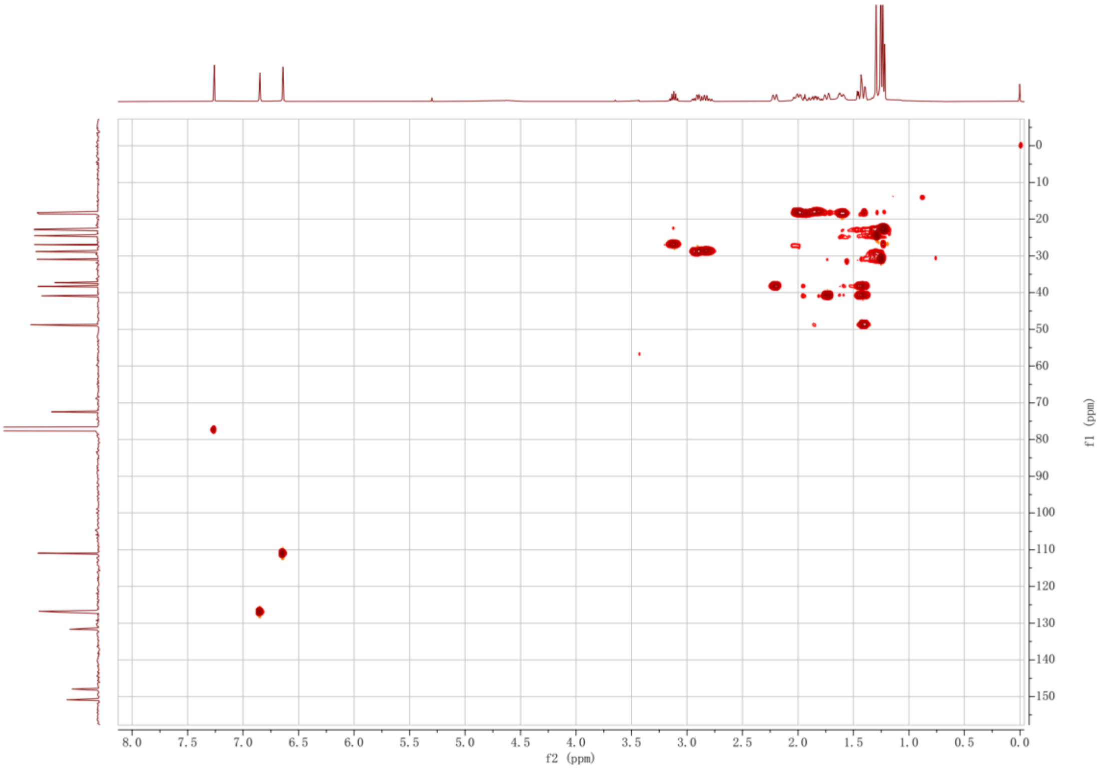

Figure S24. The HSQC spectrum of **3** in $CDCl_3$.

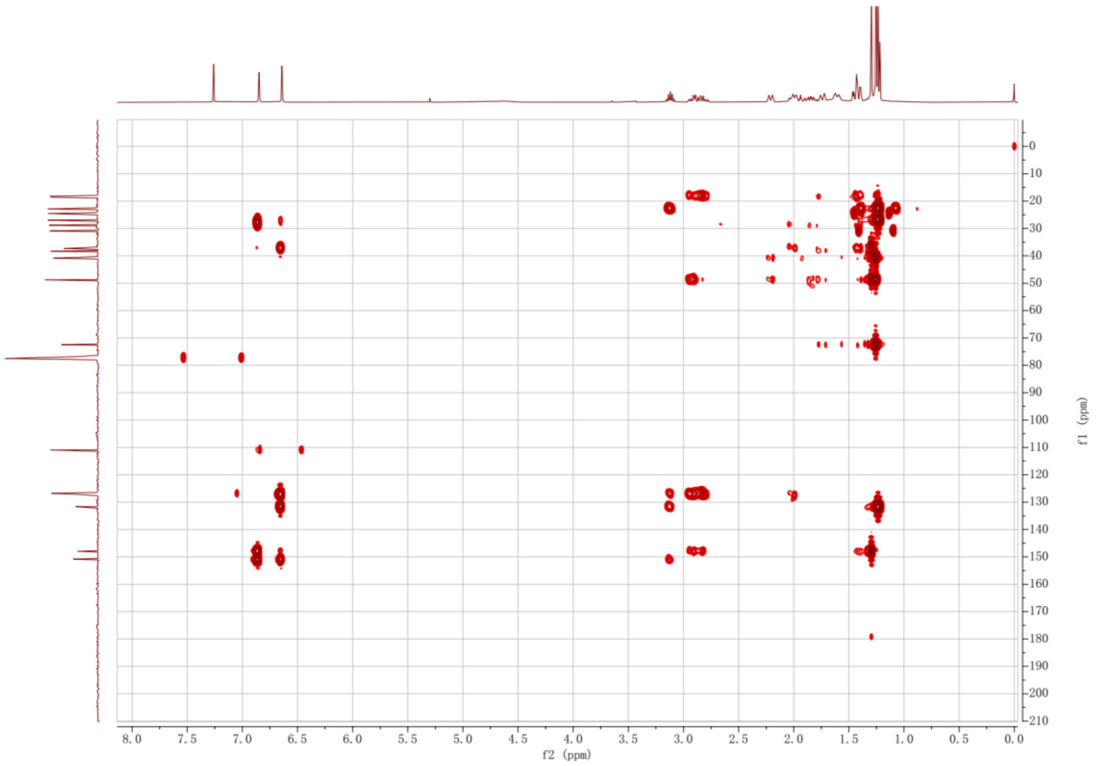

Figure S25. The HMBC spectrum of **3** in $CDCl_3$.

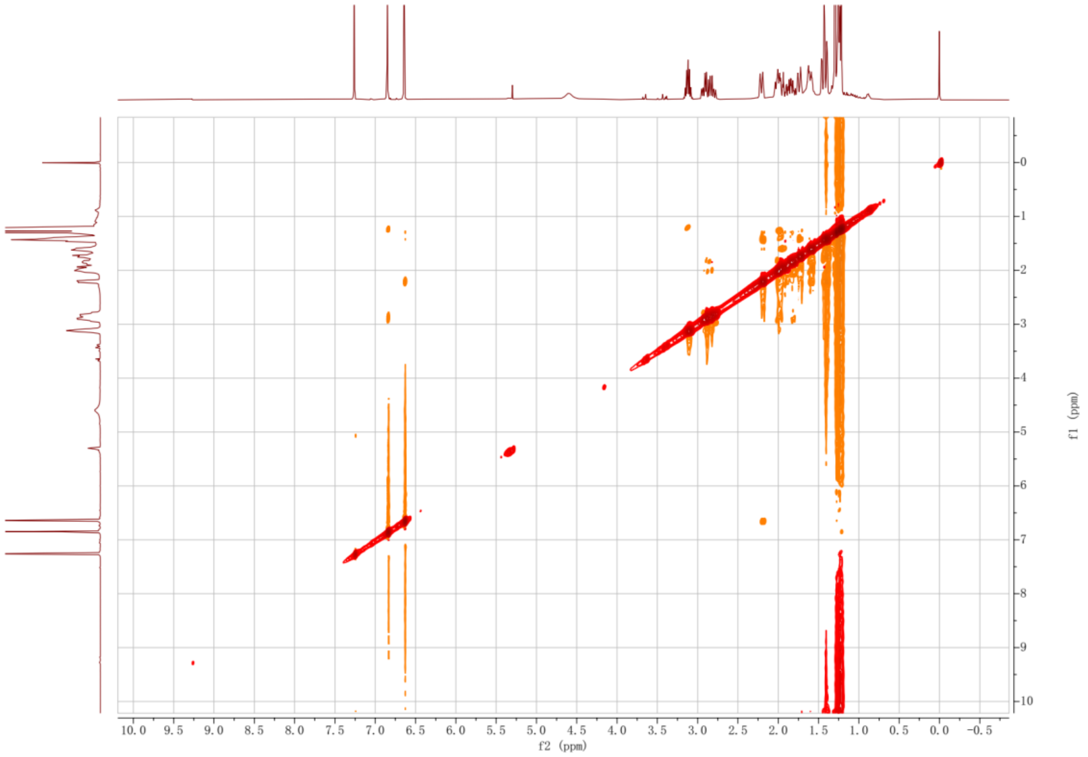

Figure S26. The NOESY spectrum of **3** in $CDCl_3$.

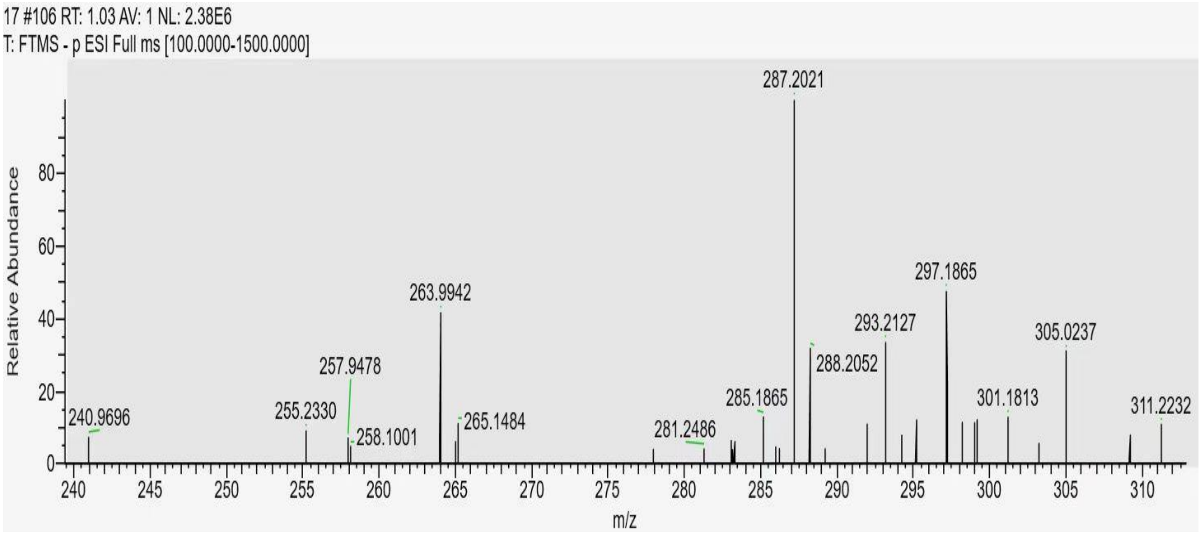

Figure S27. The HRESIMS spectrum of **3**.

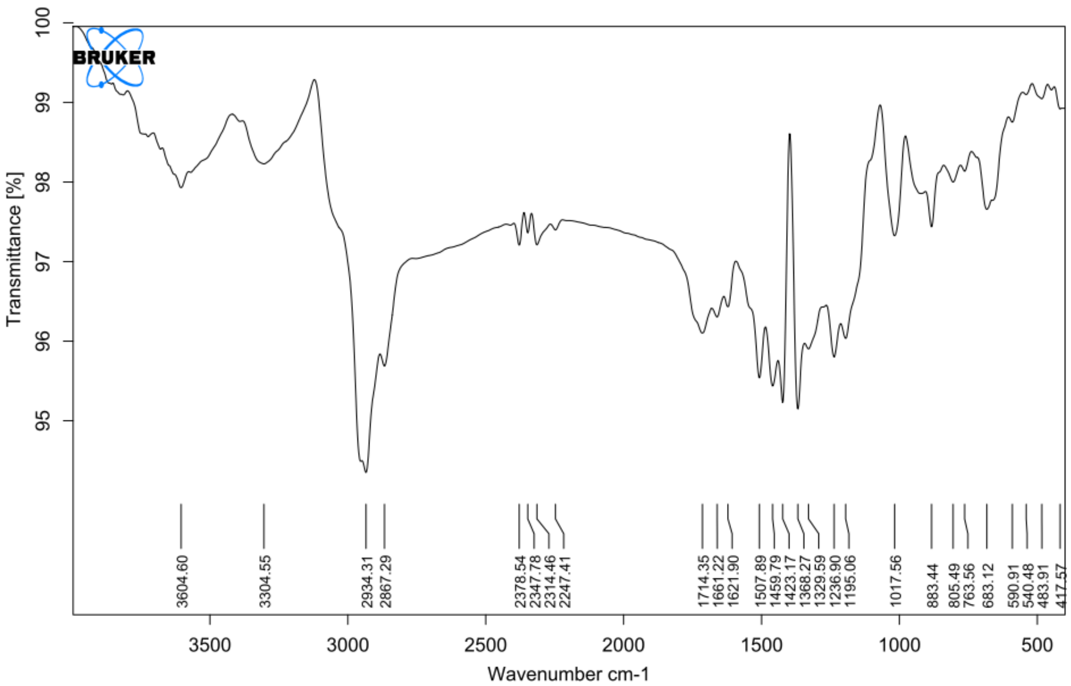

Figure S28. The IR spectrum of **3**.

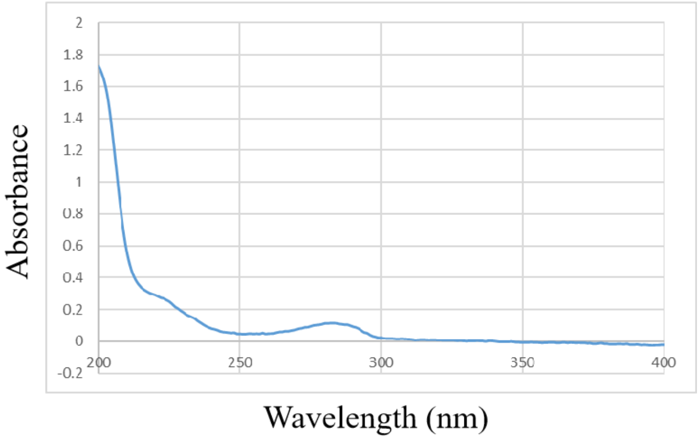

Figure S29. The UV spectrum of **3**.

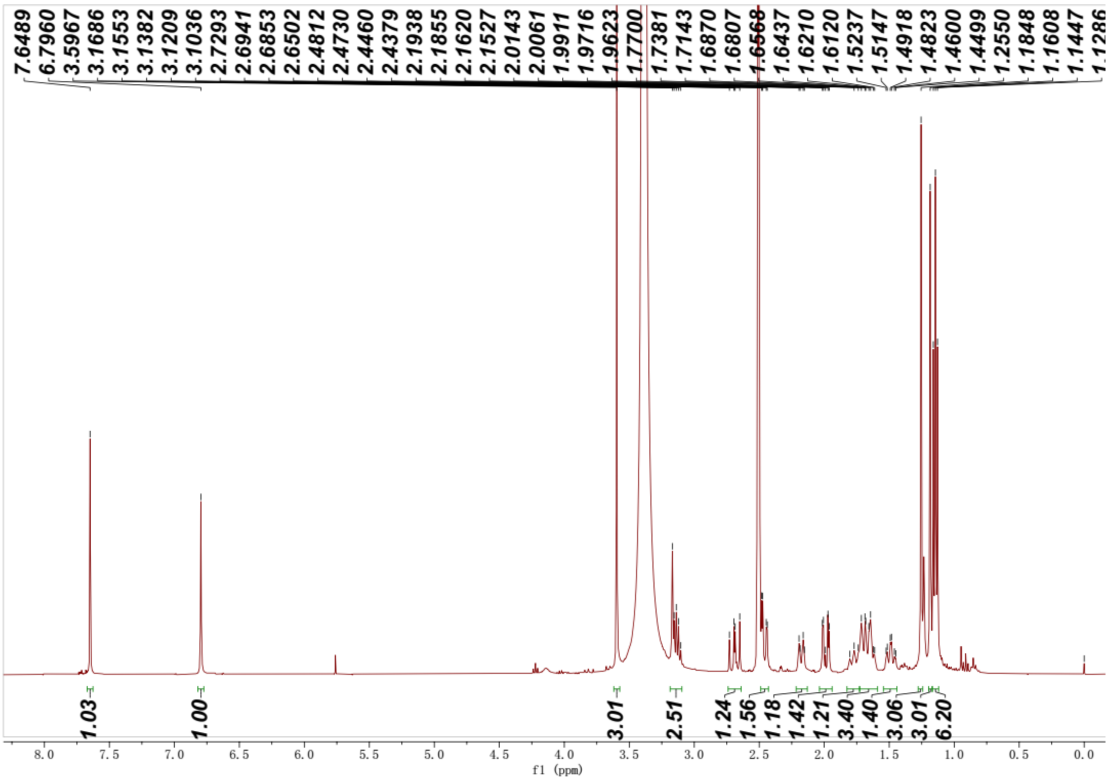

Figure S30. The ^{1}H NMR spectrum of **4** in DMSO-d_6 (400 MHz).

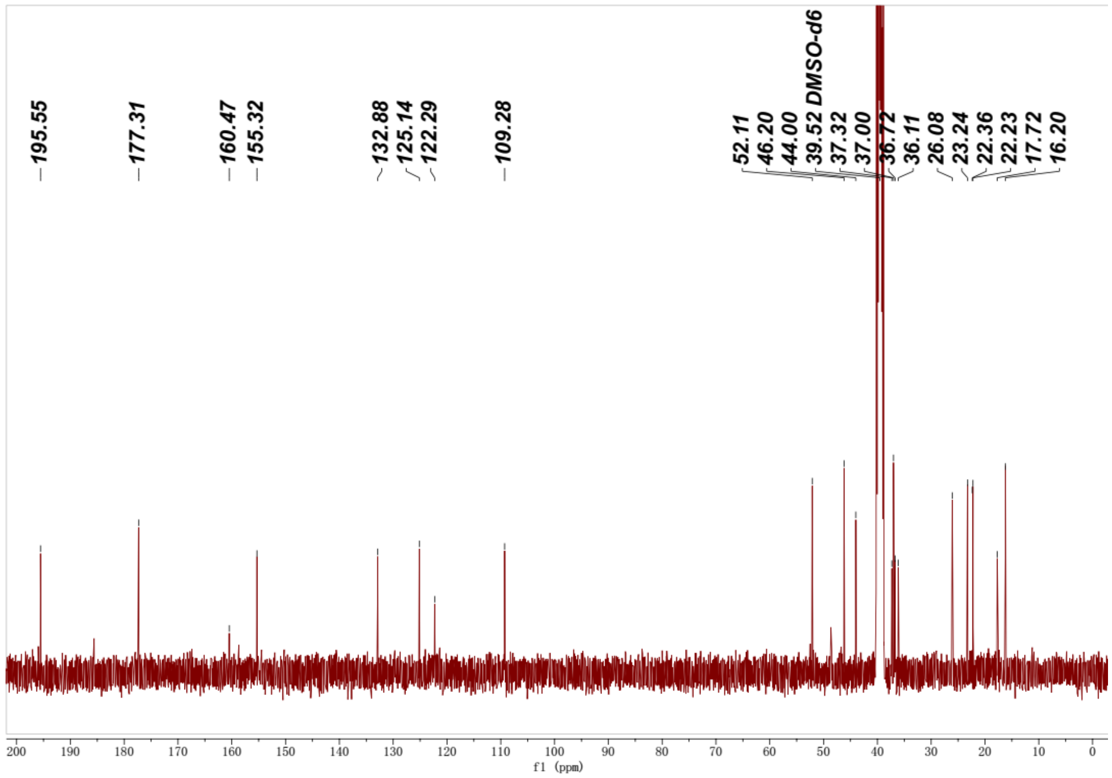

Figure S31. The ^{13}C NMR spectrum of **4** in DMSO-d_6 (100 MHz).

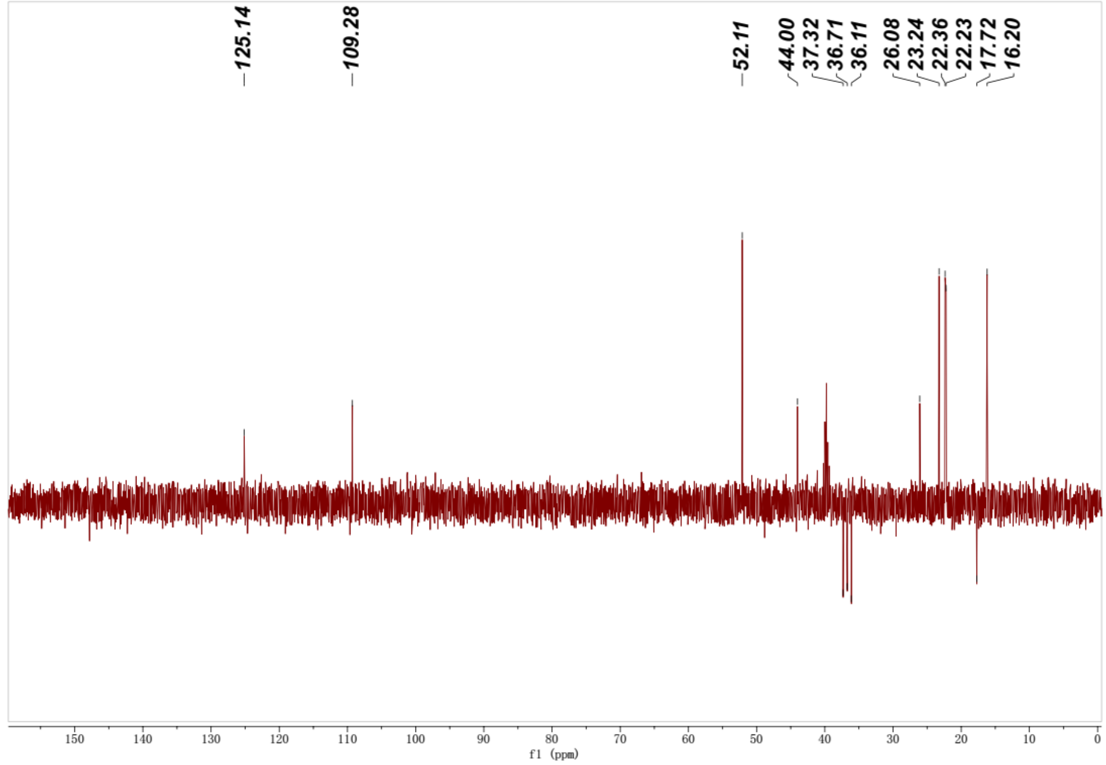

Figure S32. The DEPT135 spectrum of **4** in DMSO-d_6 (100 MHz).

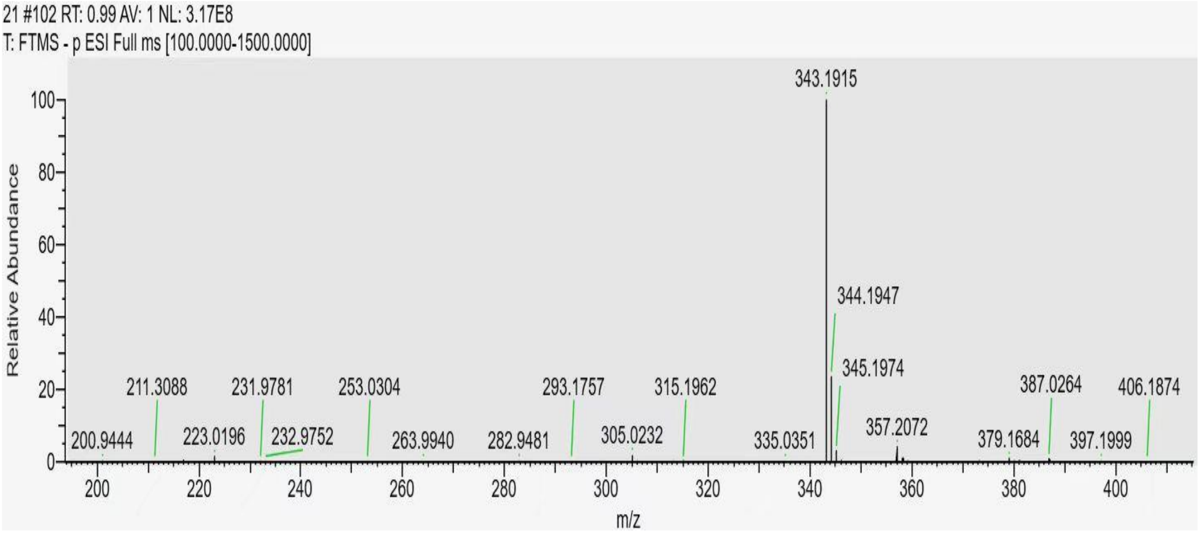

Figure S33. The HRESIMS spectrum of **4**.

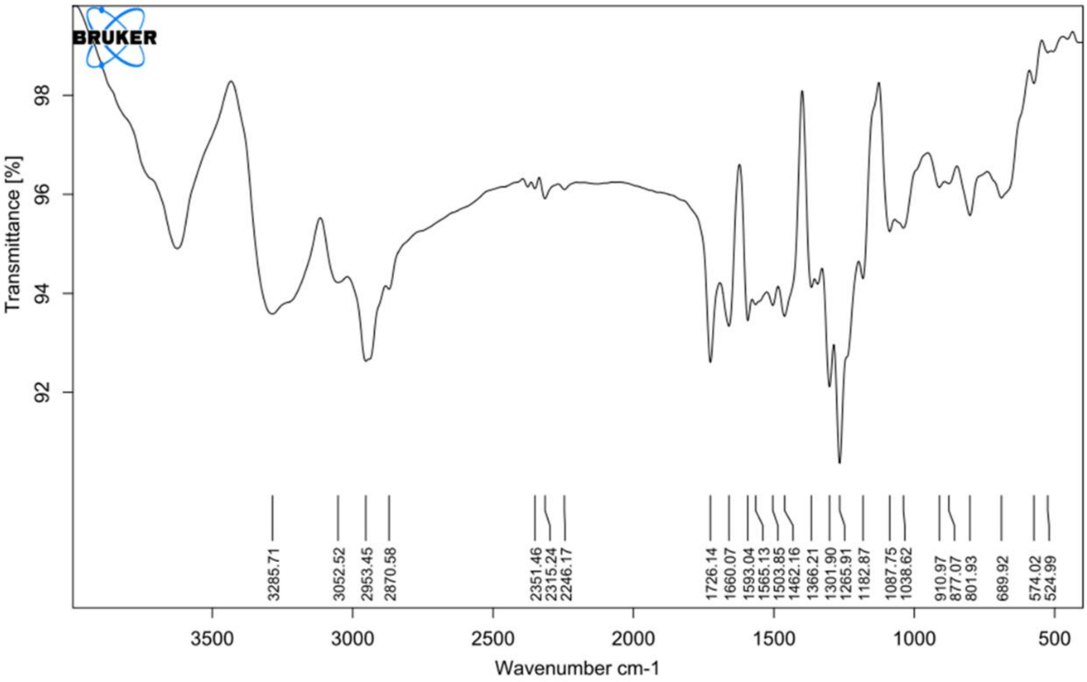

Figure S34. The IR spectrum of **4**.

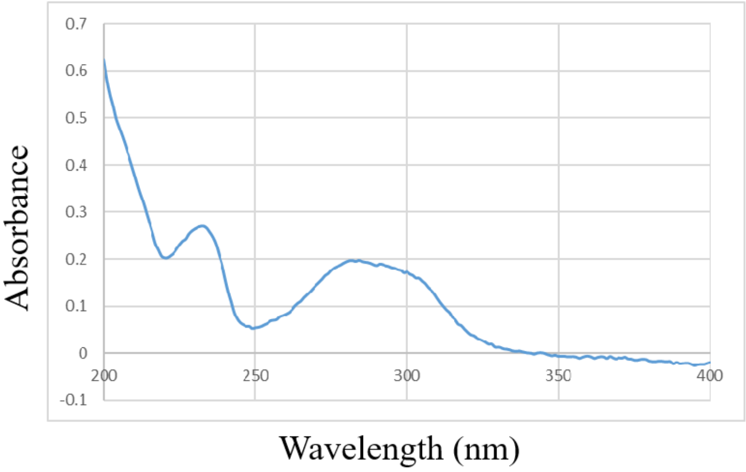

Figure S35. The UV spectrum of **4**.

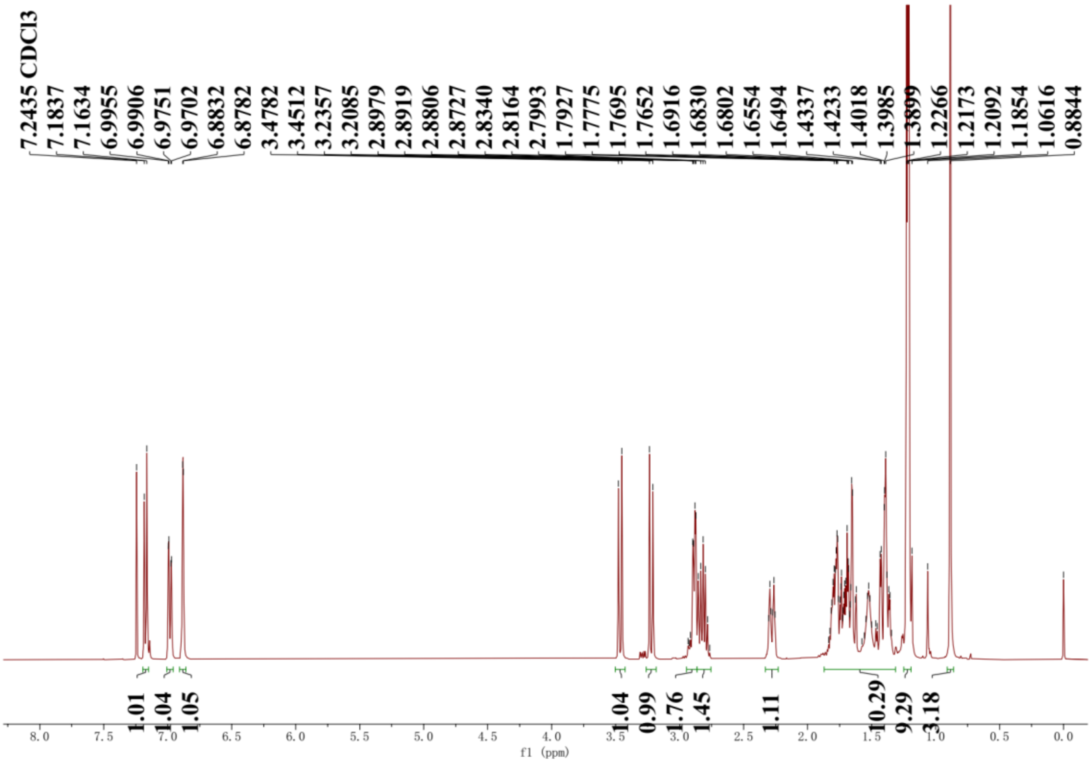

Figure S36. The ^{1}H NMR spectrum of **5** in $CDCl_3$ (400MHz).

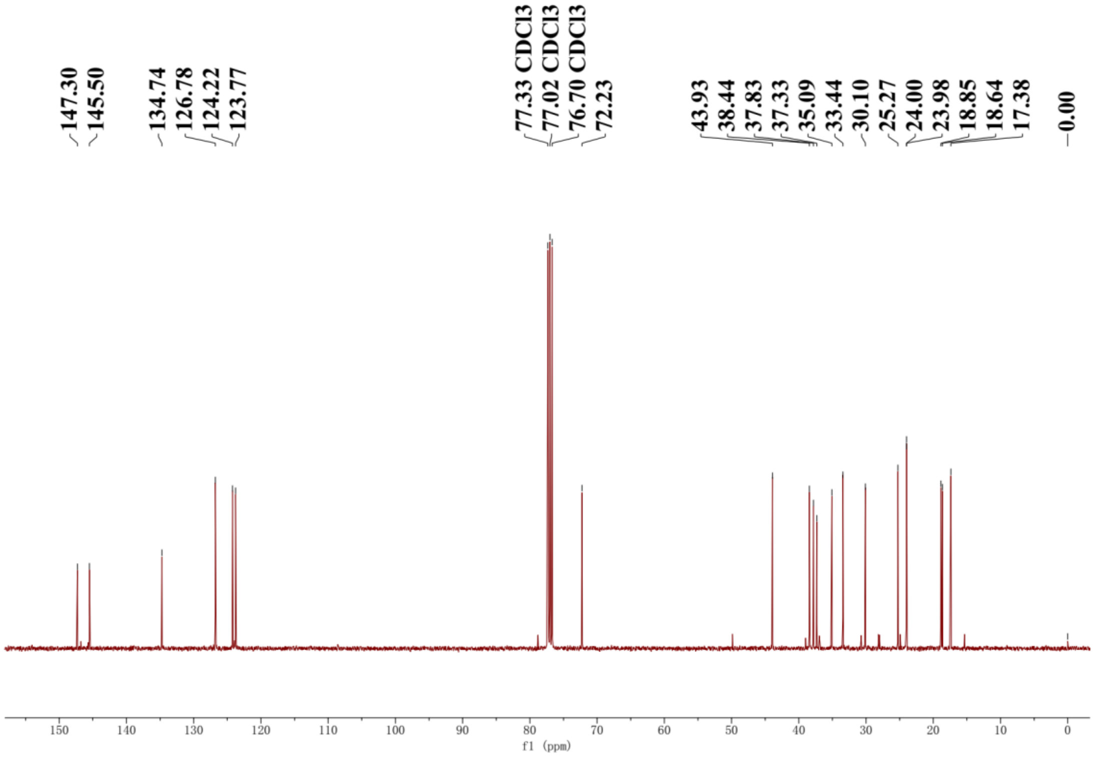

Figure S37. The ^{13}C NMR spectrum of **5** in $CDCl_3$ (100MHz).

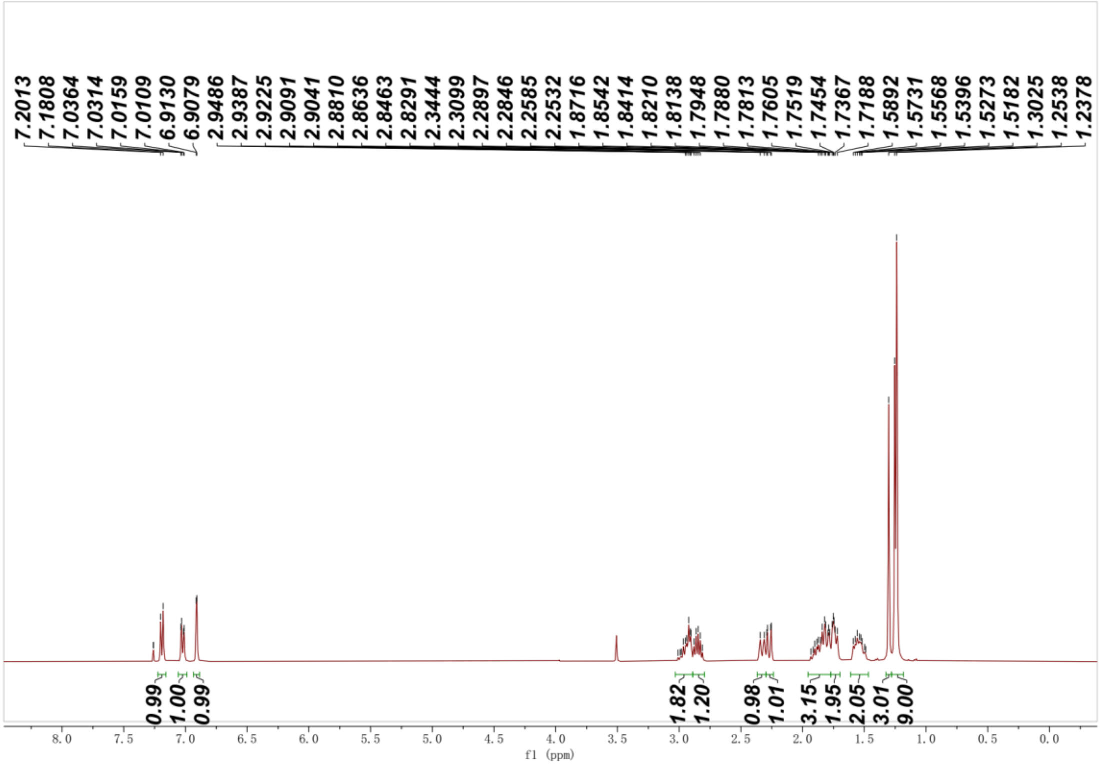

Figure S38. The ^{1}H NMR spectrum of **6** in $CDCl_3$ (400MHz).

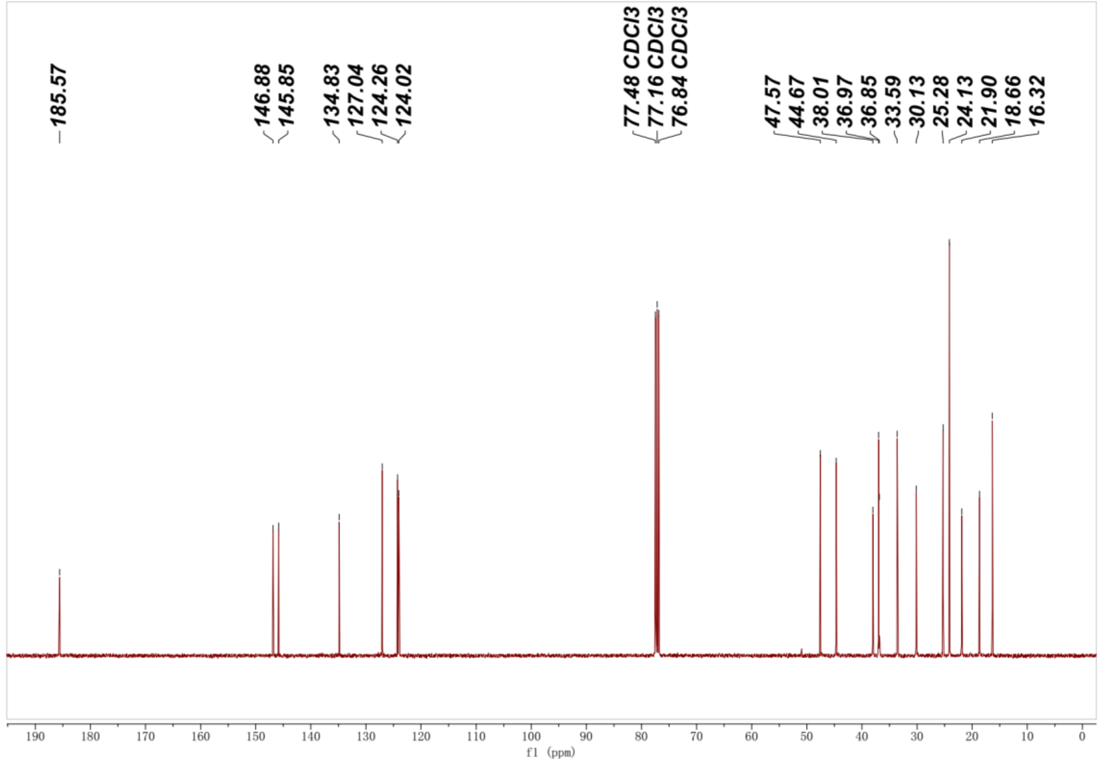

Figure S39. The ^{13}C NMR spectrum of **6** in $CDCl_3$ (100MHz).

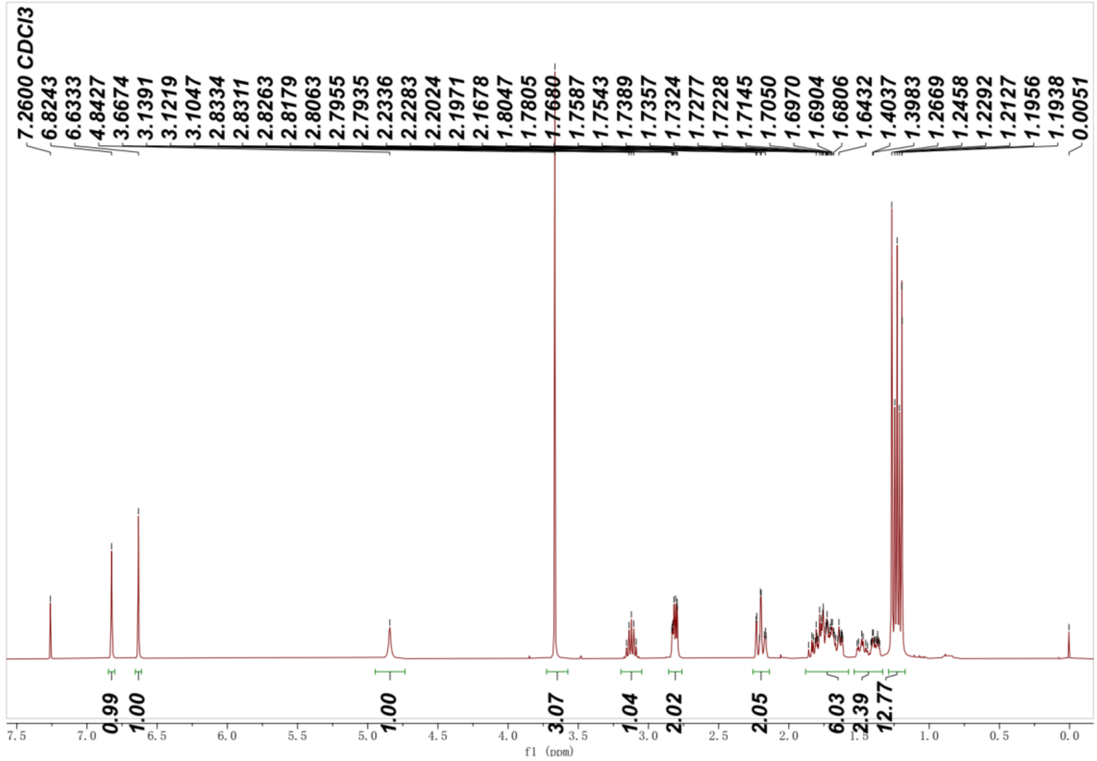

Figure S40. The ^{1}H NMR spectrum of **7** in $CDCl_3$ (400MHz).

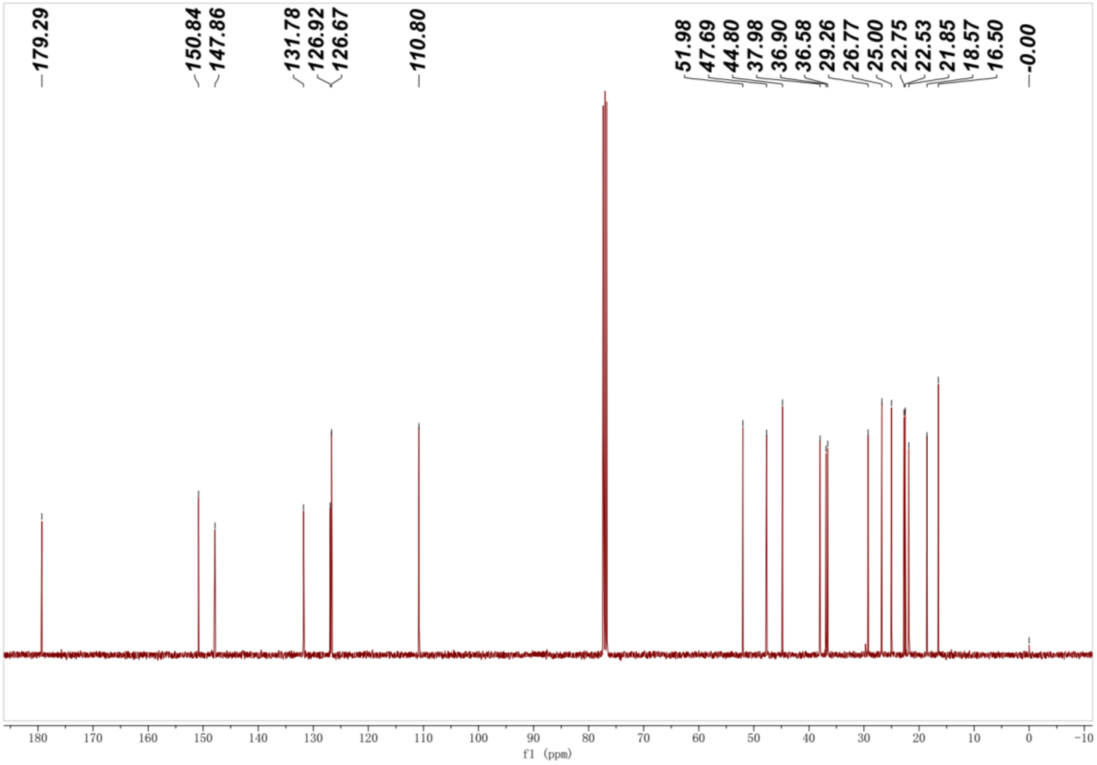

Figure S41. The ^{13}C NMR spectrum of **7** in $CDCl_3$ (100MHz).

Table S1. Conformational analysis of the B3LYP/6-31G(d) optimized conformers of **1** in the gas phase (T=298.15 K)

Conformer	E^a (Hartree)	C^b (Hartree)	G^c (kcal/mol)	ΔG^d (kcal/mol)	Population^e
1-1	-811.411525	0.308672	-508967.040554	0.0	81.38%
1-2	-811.410662	0.3092	-508966.167355	0.873199	18.62%

^{a}Electronic energy obtained at M062X/6-311+G(2d,p) level of theory; ^{b}Thermal correction to Gibbs free energy obtained at B3LYP/6-31G(d) level of theory; ^{c}Gibbs free energy (E + C); ^{d}The relative Gibbs free energy; ^{e}The Boltzmann distribution of each conformer.

Table S2. Atomic coordinates (Å) of 1-1 obtained at the B3LYP/6-31G(d) level of theory in the gas phase.

C	-2.045756	2.338283	-0.526436	H	-3.962049	1.370924	-0.884346
C	-2.892804	1.136400	-0.950051	H	-2.681668	0.920818	-2.008027
C	-2.593794	-0.133364	-0.108404	H	-0.804107	-0.553479	-1.167042
C	-1.057145	-0.385834	-0.105927	H	-0.255632	1.807136	-1.612078
C	-0.154676	0.813311	0.309629	H	0.013866	2.913192	-0.271087
C	-0.546780	2.022782	-0.575009	H	-1.361528	-2.472791	0.444415
C	-0.646296	-1.663492	0.633691	H	-0.651555	-1.498626	1.716034
C	0.745913	-2.089040	0.168790	H	1.156963	-2.858752	0.833606

C	1.724316	-0.935262	0.059314	H	0.657262	-2.563128	-0.820219
C	1.311981	0.408957	0.075634	H	3.387868	-2.290989	-0.106595
C	3.082171	-1.244827	-0.094127	H	4.396582	1.871507	-0.305094
C	4.042983	-0.246562	-0.228447	H	2.017080	2.444400	-0.024614
C	3.647156	1.093281	-0.205645	H	5.502304	-1.475717	-0.376809
C	2.301878	1.397725	-0.051282	H	-1.250636	1.610098	2.063001
O	5.376313	-0.514220	-0.379408	H	-0.056070	0.369789	2.463428
C	-0.268465	1.213046	1.800726	H	0.473119	1.987844	2.020831
C	-3.268307	-0.074629	1.266788	H	-4.343823	-0.232579	1.150569
C	-3.197295	-1.288300	-0.906478	H	-2.896177	-0.859471	1.931069
O	-4.131085	-1.979056	-0.568010	H	-3.115402	0.886835	1.759500
H	-2.262029	3.184362	-1.189910	H	-2.704693	-1.452376	-1.894863
H	-2.331968	2.662381	0.480973	-	-	-	-

Table S3. Atomic coordinates (Å) of 1-2 obtained at the B3LYP/6-31G(d) level of theory in the gas phase.

C	-2.182064	2.239062	-0.622039	H	-4.073641	1.194900	-0.886630
C	-2.997912	0.985189	-0.943966	H	-2.785687	0.694144	-1.981870
C	-2.642721	-0.210639	-0.011302	H	-0.866977	-0.702870	-1.025835
C	-1.109406	-0.422181	0.008786	H	-0.407179	1.655746	-1.702653
C	-0.237389	0.837726	0.296314	H	-0.132787	2.881812	-0.470946
C	-0.677445	1.955159	-0.681102	H	-1.343652	-2.442647	0.781458
C	-0.653687	-1.600437	0.877223	H	-0.636782	-1.319299	1.936064
C	0.738048	-2.036072	0.421290	H	1.186814	-2.726634	1.146322
C	1.682938	-0.876098	0.173296	H	0.630682	-2.604343	-0.513909
C	1.235845	0.453460	0.069654	H	3.377256	-2.200324	0.094943
C	3.044525	-1.165404	0.015350	H	4.271413	1.946720	-0.526461
C	3.975138	-0.161957	-0.239393	H	1.884707	2.486583	-0.240913
C	3.545373	1.163857	-0.333524	H	5.460010	-1.366385	-0.313148
C	2.195959	1.449159	-0.174494	H	-1.328771	1.730979	2.007412
O	5.311533	-0.412001	-0.399308	H	-0.046052	0.609311	2.477498
C	-0.331752	1.370392	1.746943	H	0.364819	2.206887	1.867054
C	-3.316307	-0.045504	1.363779	H	-4.399105	-0.201731	1.277184
C	-3.308152	-1.402418	-0.698648	H	-2.932126	-0.767532	2.090188
O	-2.749383	-2.275043	-1.324994	H	-3.169923	0.954668	1.774329
H	-2.436008	3.025887	-1.342711	H	-4.421862	-1.389963	-0.626023
H	-2.458304	2.630684	0.364058	-	-	-	-

Table S4. Key transitions, oscillator strengths, and rotatory strengths in the ECD spectrum of conformer 1-1 at the CAM-B3LYP/6-311G(d) level of theory in MeOH with IEFPCM solvent model.

Num[a]	*Transition*[b]	*CI-coeff*[c]	*ΔE (eV)*[d]	*λ (nm)*[e]	*f*[f]	R_{vel}[g]	R_{len}[h]
1	68->71	0.65503	4.3842	282.80	0.0003	-4.1872	-4.2252
2	69->73	-0.28984	5.0840	243.87	0.0559	0.099	0.2352
	70->72	0.62248					
3	69->72	0.40537	5.8918	210.43	0.0541	10.0059	9.6164
	70->73	0.55033					
4	69->72	0.23467	6.4969	190.84	0.0989	10.3791	10.8513
	70->71	0.63827					
5	69->72	0.48599	6.6365	186.82	0.8331	6.295	4.5694
	70->73	-0.4296					
6	70->75	0.65194	6.7960	182.44	0.0011	3.7201	5.7803
7	69->73	0.611	6.8225	181.73	0.4681	2.6834	-0.7318
	70->72	0.30334					
8	67->71	0.47594	7.2598	170.78	0.0095	-13.3111	-12.6243
	69->71	-0.44221					
9	66->71	-0.329	7.3669	168.30	0.0328	-8.9471	-9.4221
	67->71	0.35022					
	69->71	0.36131					
10	66->72	0.39831	7.3996	167.56	0.0119	8.0431	8.4974
	68->72	0.3689					
11	70->74	0.47447	7.4367	166.72	0.0050	-3.6747	-4.0942
	70->78	-0.44128					
12	66->71	0.47709	7.5052	165.20	0.0075	11.6336	11.2636
13	67->72	0.58694	7.6583	161.89	0.0060	-5.8344	-6.4642
14	70->74	-0.35747	7.7041	160.93	0.0092	-18.9331	-18.9199
	70->76	0.46342					
	70->77	-0.23468					
15	65->71	0.31811	7.8124	158.70	0.0087	13.1105	13.0738
	66->73	0.351					
	68->73	0.35508					
16	65->71	0.40977	7.8600	157.74	0.0005	3.8304	4.1579
	66->73	-0.28644					
	68->73	-0.27618					
17	69->75	0.61342	7.9101	156.74	0.0005	1.204	1.2109
18	70->77	0.4149	7.9751	155.46	0.0095	-3.7563	-6.1148

	70->78	-0.26723					
	70->80	-0.32326					
19	67->73	0.2793	8.0524	153.97	0.0009	-3.3775	-2.345
	68->72	0.36069					
20	67->73	0.49057	8.0828	153.39	0.0023	-13.2841	-12.2353
21	69->78	0.23635	8.1068	152.94	0.0163	-5.021	-5.4526
	70->76	0.29544					
	70->77	0.26622					
22	69->74	-0.30099	8.1388	152.34	0.0036	6.2761	6.7714
	69->78	0.36109					
	70->77	-0.24084					
23	68->72	-0.2361	8.2230	150.78	0.0240	-6.4401	-4.4774
	68->74	0.46926					
	68->76	-0.23516					
24	64->72	0.49567	8.3042	149.30	0.0310	13.2534	13.4288
	65->72	-0.33917					
25	69->74	-0.27168	8.3407	148.65	0.0052	-11.7695	-10.8461
	69->76	0.43484					
	69->77	-0.31504					
26	60->71	0.25209	8.4520	146.69	0.0163	-11.4189	-9.9446
	63->71	0.51635					
27	61->73	0.26452	8.5412	145.16	0.0030	8.811	9.313
	66->73	-0.24039					
	68->73	0.40694					
28	70->78	-0.23376	8.6024	144.13	0.0015	-3.5952	-1.8736
	70->80	0.34297					
	70->85	-0.25813					
29	62->71	0.34962	8.6133	143.94	0.0134	26.7896	26.9383
	64->72	-0.2286					
	65->72	-0.23233					
30	62->71	0.26697	8.6521	143.30	0.0304	-0.4844	-0.9469
	65->72	0.23962					
31	68->76	0.35314	8.6841	142.77	0.0190	-15.5497	-11.3681
	68->77	0.28347					
32	64->73	0.29419	8.7237	142.12	0.0098	-7.8591	-8.0179
	65->73	-0.27224					
33	69->77	-0.24709	8.7436	141.80	0.0046	-3.2678	-4.4567
	70->79	0.36718					

34	70->79	0.33599	8.7617	141.51	0.0042	-0.9697	-1.1831
	70->81	0.24369					
	70->82	0.24152					
35	61->72	-0.22849	8.8142	140.66	0.0017	4.45	4.0182
	63->72	0.29976					
	65->72	0.32667					
36	70->78	0.26964	8.8426	140.21	0.0043	-2.2412	-2.3328
	70->79	0.3524					
	70->82	-0.24694					

[a]Number of the excited states; [b]Only transitions with contribution over 10.0% were listed; [c]Configuration-interaction coefficient; [d]Excitation energy; [e]Wavelength; [f]Oscillator strength; [g]Rotatory strength in velocity form (10^{-40} cgs); [h]Rotatory strength in length form (10^{-40} cgs).

Table S5. Key transitions, oscillator strengths, and rotatory strengths in the ECD spectrum of conformer 1-2 at the CAM-B3LYP/6-311G(d) level of theory in MeOH with IEFPCM solvent model.

Num[a]	*Transition*[b]	*CI-coeff*[c]	*ΔE (eV)*[d]	*λ (nm)*[e]	*f*[f]	R_{vel}[g]	R_{len}[h]
1	68->71	0.66402	4.3545	284.73	0.0003	9.8489	5.3494
2	69->73	-0.2916	5.0864	243.76	0.0565	-0.1268	0.0263
	70->72	0.63746					
3	69->72	0.42026	5.8998	210.15	0.0505	8.1745	7.7991
	70->73	0.55949					
4	70->71	0.69158	6.4707	191.61	0.0009	-2.3792	-2.4497
5	69->72	0.55603	6.6259	187.12	0.9159	9.3357	7.7723
	70->73	-0.42208					
6	70->75	0.65278	6.7801	182.86	0.0036	14.4716	22.9713
7	69->73	0.63056	6.8302	181.52	0.4835	-21.0151	-31.4396
	70->72	0.29377					
8	67->71	0.54201	7.1922	172.39	0.0240	-12.1681	-11.9241
	69->71	0.37219					
9	66->71	0.23012	7.3111	169.58	0.0025	-3.4209	-3.0246
	67->71	-0.27915					
	69->71	0.55053					
10	66->72	-0.44336	7.3640	168.37	0.0016	-3.9531	-5.2808
	68->72	0.44934					
11	70->74	0.4542	7.4186	167.13	0.0051	-2.5624	-3.0894

	70->77	-0.23958					
	70->78	-0.36563					
	70->79	-0.24196					
12	66->71	0.53932	7.4924	165.48	0.0197	-3.5823	-2.8293
13	67->72	0.58582	7.6267	162.57	0.0053	-0.5739	-0.3514
14	65->71	0.55594	7.7345	160.30	0.0079	6.8981	7.2421
15	66->73	-0.2296	7.7755	159.46	0.0042	-13.5148	-13.6373
	68->73	0.22547					
	70->76	0.50207					
16	66->73	-0.3789	7.7955	159.05	0.0079	4.5465	5.0225
	68->73	0.40686					
	70->76	-0.29728					
17	69->75	0.62881	7.8813	157.32	0.0004	-0.7651	-0.6194
18	70->74	0.29683	7.9875	155.22	0.0133	-3.29	-5.9238
	70->76	-0.24565					
	70->79	0.34803					
19	67->73	0.46626	8.0436	154.14	0.0102	-5.069	-4.1975
	68->72	-0.27238					
20	66->72	0.29667	8.0694	153.65	0.0013	-7.5348	-6.3526
	67->73	0.32884					
	68->72	0.41252					
21	69->74	0.37338	8.1082	152.91	0.0029	5.8901	5.3526
	69->78	-0.33279					
	69->79	-0.23995					
22	70->77	0.49145	8.1782	151.60	0.0031	5.0158	4.9871
	70->78	-0.29976					
23	68->74	0.47729	8.2718	149.89	0.0530	8.634	6.6544
	68->78	0.24326					
	68->79	-0.22695					
24	64->72	0.51831	8.3106	149.19	0.0191	-9.3742	-7.6338
	65->72	-0.32876					
25	69->76	0.59541	8.3747	148.05	0.0054	-17.835	-16.6863
26	60->71	-0.2576	8.4535	146.67	0.0067	1.2448	1.1967
	61->71	0.267					
	63->71	0.50383					
27	66->73	0.31312	8.4732	146.32	0.0314	-9.9284	-10.4282
	68->73	0.38638					
	68->77	-0.29519					

28	62->71	0.30569	8.5794	144.51	0.0010	-2.3434	1.4097
	65->72	-0.22405					
29	62->71	0.30398	8.5838	144.44	0.0479	-6.1284	-6.4059
	65->72	0.24405					
30	70->80	0.3532	8.6082	144.03	0.0054	5.5243	3.3401
	70->84	0.24616					
31	68->77	0.36456	8.6698	143.01	0.0398	-43.8662	-44.7673
32	63->72	-0.27974	8.6875	142.72	0.0108	-2.8048	-2.9787
	65->72	0.33246					
33	64->73	-0.25654	8.7085	142.37	0.0009	-1.3625	-2.0661
	65->72	0.24183					
	65->73	0.26544					
	70->82	0.25279					
34	64->73	0.31443	8.7522	141.66	0.0002	0.8016	0.719
	65->73	-0.28042					
	70->78	-0.24949					
	70->81	0.27916					
	70->82	0.24779					
35	69->74	0.34475	8.8021	140.86	0.0076	-4.6267	-4.4063
	69->79	0.30769					
36	70->77	0.25789	8.8502	140.09	0.0056	-6.9886	-7.9065
	70->81	0.36621					

[a]Number of the excited states; [b]Only transitions with contribution over 10.0% were listed; [c]Configuration-interaction coefficient; [d]Excitation energy; [e]Wavelength; [f]Oscillator strength; [g]Rotatory strength in velocity form (10^{-40} cgs); [h]Rotatory strength in length form (10^{-40} cgs).

Table S6. Conformational analysis of the B3LYP/6-31G(d) optimized conformers of 3 in the gas phase (T=298.15 K)

Conformer	E[a] (Hartree)	C[b] (Hartree)	G[c] (kcal/mol)	ΔG[d] (kcal/mol)	Population[e]
3-1	-891.24159	0.383361	-559013.538916	0.0	34.68%
3-2	-891.241195	0.38317	-559013.410408	0.128508	27.91%
3-3	-891.240499	0.383506	-559012.763177	0.775739	9.35%
3-4	-891.240373	0.383546	-559012.659114	0.879801	7.85%
3-5	-891.240371	0.383545	-559012.658473	0.880443	7.84%
3-6	-891.240101	0.383378	-559012.593715	0.945201	7.03%
3-7	-891.24048	0.384015	-559012.431823	1.107093	5.35%

[a]Electronic energy obtained at M062X/6-311+G(2d,p) level of theory; [b]Thermal correction to Gibbs free energy obtained at B3LYP/6-31G(d) level of theory; [c]Gibbs free energy (E + C); [d]The relative Gibbs free energy; [e]The Boltzmann distribution of each conformer.

Table S7. Atomic coordinates (Å) of 3-1 obtained at the B3LYP/6-31G(d) level of theory in the gas phase.

C	-3.246014	2.030590	-0.494835	H	-1.790623	-0.779758	-1.252750
C	-3.997360	0.783133	-0.954648	H	-1.432398	1.666658	-1.614034
C	-3.595523	-0.481940	-0.184683	H	-1.230757	2.740053	-0.233857
C	-2.049519	-0.642385	-0.191040	H	-2.159423	-2.764486	0.317613
C	-1.249670	0.617357	0.272687	H	-1.597740	-1.739381	1.630214
C	-1.730537	1.824263	-0.567884	H	0.353974	-2.913648	0.809203
C	-1.542658	-1.889586	0.544894	H	-0.120220	-2.670747	-0.860017
C	-0.099914	-2.183782	0.126805	H	2.539290	-2.143723	-0.114656
C	0.774571	-0.949416	0.041449	H	0.774286	2.444696	-0.030364
C	0.247904	0.350882	0.056258	H	4.921558	0.493819	-1.050071
C	2.158794	-1.126005	-0.097123	H	-1.008957	0.152913	2.407560
C	3.059793	-0.072536	-0.217409	H	-0.881004	1.861589	2.016217
C	2.514662	1.222969	-0.190074	H	-2.477998	1.073082	2.044089
C	1.145271	1.423143	-0.053997	H	-3.822934	-1.068966	1.672162
C	4.562914	-0.257733	-0.335459	H	-4.178847	-2.596189	-0.217535
C	-1.428739	0.942964	1.777878	H	-5.357896	-1.490583	-0.928143
O	-4.119411	-0.310523	1.144925	H	-3.879704	-1.909231	-1.823056
C	-4.287082	-1.694150	-0.828767	H	2.879718	3.101889	-0.289688
O	3.389933	2.277640	-0.301248	H	6.336216	-0.044854	0.922271
C	5.246354	0.035497	1.014280	H	5.004313	1.043292	1.362623
C	4.973247	-1.638022	-0.860693	H	4.914559	-0.681222	1.775139
H	-3.530380	2.885131	-1.121167	H	6.054792	-1.667109	-1.032576
H	-3.545354	2.279535	0.528703	H	4.470980	-1.877277	-1.804451
H	-5.079679	0.919170	-0.847349	H	4.737872	-2.431054	-0.140948
H	-3.796785	0.607995	-2.020379	-	-	-	-

Table S8. Atomic coordinates (Å) of 3-2 obtained at the B3LYP/6-31G(d) level of theory in the gas phase.

C	-3.245385	2.031517	-0.478456	H	-1.779840	-0.773025	-1.246369
C	-3.981084	0.787247	-0.968450	H	-1.426151	1.687915	-1.591918
C	-3.585477	-0.485453	-0.196606	H	-1.235188	2.745428	-0.197899
C	-2.045229	-0.643351	-0.185011	H	-2.164044	-2.761251	0.319666

C	-1.248479	0.617282	0.285425	H	-1.634824	-1.737008	1.635147
C	-1.728978	1.831339	-0.545051	H	0.350194	-2.901689	0.849544
C	-1.547777	-1.889405	0.555728	H	-0.106666	-2.684549	-0.827412
C	-0.100673	-2.183334	0.152836	H	2.538267	-2.144286	-0.098792
C	0.774182	-0.949583	0.055463	H	0.774471	2.444694	-0.042723
C	0.248255	0.351223	0.062451	H	4.918325	0.485864	-1.061533
C	2.157951	-1.126399	-0.087374	H	-0.994090	0.139045	2.413337
C	3.058733	-0.073862	-0.218684	H	-0.878826	1.853675	2.032865
C	2.513868	1.221619	-0.199415	H	-2.468988	1.045930	2.069355
C	1.144952	1.422778	-0.060114	H	-4.990868	-0.356149	1.159929
C	4.561563	-0.259931	-0.339909	H	-3.862574	-1.894651	-1.849904
C	-1.420888	0.931653	1.793244	H	-4.155809	-2.594045	-0.242007
O	-4.021099	-0.393394	1.171849	H	-5.343259	-1.499596	-0.966468
C	-4.268863	-1.695095	-0.852364	H	2.877761	3.099908	-0.311740
O	3.388746	2.276166	-0.322055	H	4.919162	-0.666759	1.772851
C	5.249049	0.043836	1.005464	H	6.338716	-0.037415	0.911141
C	4.970451	-1.644401	-0.855143	H	5.007963	1.054372	1.346472
H	-3.531338	2.898367	-1.086720	H	4.736949	-2.431447	-0.128288
H	-3.551001	2.253728	0.549862	H	6.051536	-1.675013	-1.029972
H	-5.069718	0.921948	-0.891520	H	4.465230	-1.891319	-1.795352
H	-3.763574	0.629077	-2.034113	-	-	-	-

Table S9. Atomic coordinates (Å) of 3-3 obtained at the B3LYP/6-31G(d) level of theory in the gas phase.

C	-3.111461	2.158233	-0.411930	H	-1.819816	-0.698243	-1.295304
C	-3.929871	0.992579	-0.962353	H	-1.297276	1.734015	-1.507860
C	-3.634249	-0.339597	-0.260165	H	-1.060846	2.714376	-0.065771
C	-2.102319	-0.602673	-0.234972	H	-2.365582	-2.737753	0.148705
C	-1.235933	0.572982	0.320866	H	-1.775648	-1.826872	1.531553
C	-1.611734	1.854029	-0.461493	H	0.115575	-3.084650	0.691791
C	-1.699816	-1.919954	0.441119	H	-0.291041	-2.712508	-0.971458
C	-0.268454	-2.287047	0.043290	H	2.388864	-2.402947	-0.103760
C	0.687199	-1.111658	0.056084	H	0.910640	2.271809	0.181276
C	0.247169	0.218863	0.129812	H	4.546438	-1.834162	-0.229615
C	2.060914	-1.366037	-0.045460	H	-0.850255	1.699000	2.139797
C	3.036714	-0.372504	-0.075998	H	-2.486147	0.995474	2.084499
C	2.576433	0.953605	0.004506	H	-1.076677	-0.019288	2.430984
C	1.216339	1.231007	0.108780	H	-3.971495	-1.025672	1.545975

C	4.506702	-0.737175	-0.187589	H	-3.965326	-1.638766	-1.993455
C	-1.438207	0.825276	1.837094	H	-4.347540	-2.406284	-0.443104
O	-4.186300	-0.212193	1.062865	H	-5.436555	-1.184074	-1.105129
C	-4.383457	-1.461798	-0.996085	H	3.035333	2.813789	0.044602
O	3.505462	1.968189	-0.019012	H	6.175205	-0.559325	-1.575894
C	5.140219	-0.208160	-1.486612	H	4.584026	-0.559595	-2.362717
C	5.310757	-0.298652	1.049159	H	5.146249	0.884890	-1.501568
H	-3.321431	3.064143	-0.993876	H	4.878621	-0.719036	1.963988
H	-3.421740	2.368893	0.616998	H	5.317899	0.790370	1.143727
H	-5.003423	1.194599	-0.873133	H	6.348394	-0.644712	0.971084
H	-3.711407	0.866647	-2.031474	-	-	-	-

Table S10. Atomic coordinates (Å) of 3-4 obtained at the B3LYP/6-31G(d) level of theory in the gas phase.

C	-3.250001	2.035525	-0.479560	H	-1.800566	-0.773294	-1.258007
C	-4.005342	0.790460	-0.939577	H	-1.444390	1.674180	-1.612068
C	-3.599171	-0.478730	-0.178666	H	-1.230488	2.741455	-0.230377
C	-2.053184	-0.639473	-0.194256	H	-2.161442	-2.763737	0.307847
C	-1.250733	0.618310	0.269482	H	-1.590775	-1.743315	1.620511
C	-1.735239	1.828828	-0.563442	H	0.353349	-2.919807	0.779174
C	-1.543011	-1.889743	0.534325	H	-0.131245	-2.661482	-0.885072
C	-0.103161	-2.183069	0.105795	H	2.535100	-2.141674	-0.131679
C	0.772458	-0.948912	0.025388	H	0.787878	2.452585	-0.045564
C	0.246273	0.354643	0.045785	H	4.945979	0.441374	-1.085218
C	2.153868	-1.124347	-0.113828	H	-0.871358	1.855638	2.014654
C	3.053538	-0.065975	-0.231239	H	-2.468298	1.066625	2.049993
C	2.508159	1.230756	-0.203044	H	-0.997112	0.145541	2.401711
C	1.137847	1.427286	-0.065675	H	-3.810113	-1.073187	1.677528
C	4.559070	-0.264576	-0.329500	H	-4.182740	-2.592963	-0.219405
C	-1.420545	0.937960	1.777128	H	-5.365842	-1.483545	-0.917131
O	-4.115666	-0.315213	1.154862	H	-3.893066	-1.897316	-1.823118
C	-4.294451	-1.687639	-0.825208	H	4.196199	2.110186	-0.476504
O	3.279434	2.364322	-0.296800	H	6.329177	-0.005467	0.934763
C	5.238106	0.065015	1.016210	H	4.982210	1.071495	1.363880
C	4.972903	-1.660466	-0.808404	H	4.904547	-0.641490	1.784237
H	-3.537891	2.892640	-1.100685	H	6.056318	-1.698963	-0.963867
H	-3.542712	2.280889	0.546783	H	4.480182	-1.924660	-1.749678

H	-5.086969	0.926072	-0.824372	H	4.721777	-2.425877	-0.065628
H	-3.812157	0.620118	-2.007494	-	-	-	-

Table S11. Atomic coordinates (Å) of 3-5 obtained at the B3LYP/6-31G(d) level of theory in the gas phase.

C	-3.249910	2.035277	-0.480614	H	-1.800592	-0.773698	-1.257675
C	-4.005289	0.790002	-0.939939	H	-1.444226	1.673187	-1.612833
C	-3.599148	-0.478867	-0.178475	H	-1.230368	2.741272	-0.231780
C	-2.053172	-0.639578	-0.193953	H	-2.161342	-2.763701	0.308969
C	-1.250773	0.618402	0.269330	H	-1.590155	-1.742883	1.621066
C	-1.735160	1.828473	-0.564317	H	0.353584	-2.919733	0.779305
C	-1.542824	-1.889630	0.534895	H	-0.131472	-2.661564	-0.884829
C	-0.103115	-2.183054	0.105985	H	2.535125	-2.141570	-0.131933
C	0.772455	-0.948885	0.025388	H	0.787805	2.452622	-0.044830
C	0.246271	0.354667	0.045951	H	4.946013	0.441891	-1.084814
C	2.153859	-1.124255	-0.113890	H	-0.998008	0.146578	2.401876
C	3.053522	-0.065855	-0.231156	H	-0.871436	1.856430	2.014036
C	2.508148	1.230872	-0.202615	H	-2.468715	1.068153	2.049211
C	1.137835	1.427350	-0.065215	H	-3.808557	-1.071588	1.678469
C	4.559029	-0.264530	-0.329568	H	-3.893030	-1.898098	-1.822402
C	-1.420925	0.938859	1.776765	H	-4.182576	-2.593141	-0.218376
O	-4.115735	-0.314857	1.154943	H	-5.365779	-1.484081	-0.916465
C	-4.294363	-1.688069	-0.824539	H	4.196295	2.110414	-0.475152
O	3.279402	2.364486	-0.295988	H	4.904713	-0.642771	1.783979
C	5.238181	0.064157	1.016287	H	6.329242	-0.006310	0.934669
C	4.972684	-1.660128	-0.809413	H	4.982339	1.070445	1.364575
H	-3.537751	2.892054	-1.102232	H	4.479939	-1.923636	-1.750873
H	-3.542641	2.281296	0.545575	H	4.721436	-2.426016	-0.067159
H	-5.086916	0.925691	-0.824776	H	6.056099	-1.698694	-0.964882
H	-3.812211	0.619167	-2.007808	-	-	-	-

Table S12. Atomic coordinates (Å) of 3-6 obtained at the B3LYP/6-31G(d) level of theory in the gas phase.

C	-3.112074	2.158169	-0.392903	H	-1.805573	-0.692781	-1.288845
C	-3.912130	0.996855	-0.976005	H	-1.289670	1.757224	-1.480764
C	-3.623015	-0.343857	-0.275347	H	-1.067593	2.718694	-0.023497

C	-2.097152	-0.604448	-0.230126	H	-2.369370	-2.734408	0.148936
C	-1.235423	0.571961	0.334804	H	-1.818303	-1.821863	1.535787
C	-1.610895	1.860839	-0.434685	H	0.110821	-3.072058	0.743151
C	-1.706018	-1.919947	0.452389	H	-0.274717	-2.732085	-0.931253
C	-0.268941	-2.287724	0.075965	H	2.388477	-2.402998	-0.079868
C	0.686830	-1.111742	0.075181	H	0.910245	2.273715	0.164676
C	0.247306	0.219631	0.137776	H	4.545534	-1.834745	-0.218364
C	2.060370	-1.365670	-0.030017	H	-2.481606	0.960188	2.109519
C	3.035802	-0.372094	-0.074144	H	-1.061920	-0.036740	2.437003
C	2.575467	0.954374	-0.005376	H	-0.853455	1.688376	2.159000
C	1.215662	1.232184	0.101530	H	-5.059502	-0.204129	1.047168
C	4.505525	-0.737415	-0.187480	H	-3.944637	-1.619379	-2.026371
C	-1.433109	0.809700	1.853539	H	-4.318868	-2.405674	-0.476390
O	-4.095212	-0.307907	1.083711	H	-5.419470	-1.193298	-1.147932
C	-4.361961	-1.462379	-1.025724	H	3.032795	2.815111	0.016618
O	3.503950	1.969860	-0.043311	H	4.575110	-0.582101	-2.364469
C	5.134351	-0.221492	-1.494029	H	5.139671	0.871383	-1.520118
C	5.313894	-0.285947	1.041784	H	6.169248	-0.573025	-1.583579
H	-3.323314	3.076222	-0.954832	H	6.351581	-0.632004	0.963613
H	-3.431896	2.340449	0.639029	H	4.885179	-0.697309	1.962286
H	-4.991463	1.198620	-0.918159	H	5.320383	0.804004	1.125235
H	-3.674535	0.888997	-2.043580	-	-	-	-

Table S13. Atomic coordinates (Å) of 3-7 obtained at the B3LYP/6-31G(d) level of theory in the gas phase.

C	-3.209807	2.063680	-0.448830	H	-1.710891	-0.694888	-1.300030
C	-3.935024	0.850122	-1.026535	H	-1.336901	1.760163	-1.484217
C	-3.575381	-0.464456	-0.320956	H	-1.208952	2.746591	-0.034107
C	-2.031089	-0.627312	-0.248233	H	-2.167467	-2.778386	0.115911
C	-1.262694	0.597730	0.342520	H	-1.672208	-1.841795	1.519395
C	-1.692710	1.856139	-0.448690	H	0.314693	-2.973426	0.715865
C	-1.563828	-1.921539	0.430708	H	-0.069672	-2.598699	-0.952652
C	-0.101343	-2.190463	0.069067	H	2.547942	-2.139568	0.020939
C	0.775688	-0.956018	0.128967	H	0.788192	2.441020	0.315130
C	0.246544	0.344416	0.203468	H	5.055650	0.438434	0.699129
C	2.163871	-1.124672	0.076508	H	-1.152933	0.000458	2.455629
C	3.067135	-0.062838	0.095177	H	-1.003087	1.729707	2.178127

C	2.518185	1.230133	0.176666	H	-2.597909	0.943879	2.058724
C	1.141318	1.419584	0.236131	H	-3.904291	-1.172857	1.477865
C	4.572732	-0.254136	-0.012335	H	-4.148171	-2.571520	-0.533014
C	-1.533969	0.829060	1.851136	H	-5.290806	-1.418482	-1.227578
O	-4.176040	-0.383044	0.984403	H	-3.765407	-1.773956	-2.068389
C	-4.226212	-1.628817	-1.084682	H	4.224161	2.114945	0.249833
O	3.290609	2.366560	0.199428	H	4.689531	-1.943172	1.370602
C	5.051356	-1.656985	0.377747	H	4.706108	-2.408793	-0.340784
C	5.064160	0.106178	-1.429656	H	6.145940	-1.691922	0.386437
H	-3.457388	2.955751	-1.037302	H	4.760727	1.117882	-1.719348
H	-3.566912	2.252589	0.569120	H	4.632844	-0.587213	-2.160016
H	-5.021740	0.981802	-0.968974	H	6.156486	0.040787	-1.496793
H	-3.677694	0.743704	-2.089221	-	-	-	-

Table S14. Key transitions, oscillator strengths, and rotatory strengths in the ECD spectrum of conformer 3-1 at the CAM-B3LYP/6-311G(d) level of theory in MeOH with IEFPCM solvent model.

Num[a]	*Transition*[b]	*CI-coeff*[c]	*ΔE (eV)*[d]	*λ (nm)*[e]	*f*[f]	R_{vel}[g]	R_{len}[h]
1	78->81	-0.2881	5.0385	246.07	0.1033	5.2629	5.1388
	79->80	0.63968					
2	78->80	0.37773	5.7817	214.44	0.0512	15.2642	14.7191
	79->81	0.58967					
3	78->80	0.23421	6.5305	189.85	0.0509	-23.2602	-39.3424
	79->82	-0.23036					
	79->83	0.54703					
4	78->80	0.5362	6.5768	188.52	0.5350	-2.1111	12.9737
	79->81	-0.34235					
5	78->81	0.62452	6.6415	186.68	0.9308	11.9218	16.2841
	79->80	0.28565					
6	79->82	0.49001	7.2361	171.34	0.0066	-7.5151	-8.8875
	79->84	0.37708					
7	75->80	-0.35497	7.3583	168.50	0.0065	-1.2108	-1.2478
	77->80	0.50428					
8	79->82	-0.24066	7.5390	164.46	0.0157	25.1424	27.1588
	79->84	0.49372					
9	76->80	0.34773	7.6032	163.07	0.0052	-7.2957	-1.3247
	77->82	0.35368					

10	76->80	0.35573	7.6095	162.93	0.0072	-16.9312	-16.7894
	78->83	0.32973					
11	77->82	0.26663	7.6377	162.33	0.0033	-3.9665	-4.6392
	78->83	0.34773					
12	75->81	-0.33845	7.7317	160.36	0.0030	-0.9411	-0.6644
	77->81	0.50115					
13	79->86	0.44236	7.7999	158.96	0.0001	2.5207	2.6806
	79->88	0.24245					
	79->90	-0.28639					
14	79->85	0.47355	7.9129	156.69	0.0046	-5.3644	-4.3868
	79->87	0.30082					
	79->89	0.2401					
15	76->81	0.42194	7.9628	155.70	0.0086	-10.3765	-10.5344
16	76->81	0.34835	7.9748	155.47	0.0081	16.3954	17.0019
	79->85	0.26813					
	79->86	0.23299					
17	78->82	0.38154	8.0719	153.60	0.0011	1.191	0.0096
	78->84	0.27204					
	79->87	0.33049					
18	73->80	0.25908	8.2251	150.74	0.0045	-6.3749	-6.6355
	74->80	0.43704					
	78->84	-0.22509					
19	71->80	-0.22955	8.2548	150.20	0.0205	1.3285	0.7989
	78->82	-0.22729					
	78->84	0.36686					
20	71->80	0.31786	8.2643	150.02	0.0072	16.0687	14.8162
	73->80	0.26212					
	74->80	0.30348					
	77->80	0.24596					
21	79->89	0.32613	8.3953	147.68	0.0057	5.5497	3.2375
	79->90	-0.3124					
22	72->80	0.32989	8.4260	147.14	0.0014	3.7434	1.8322
	76->80	0.26412					
23	79->85	0.22601	8.4506	146.72	0.0037	-0.0035	0.0214
	79->86	-0.26076					
	79->88	0.36245					
24	78->86	0.35306	8.4949	145.95	0.0062	8.5636	8.6905
	78->88	0.23687					

	78->90	-0.24906					
25	73->81	0.23854	8.5573	144.89	0.0056	9.7443	9.2966
	74->81	0.34705					
	79->91	0.45303					
26	75->81	0.25476	8.5766	144.56	0.0012	-12.4691	-9.5485
	77->81	0.25367					
	77->85	0.29317					
27	76->82	0.54126	8.6210	143.82	0.0017	-10.2353	-8.5423
28	73->81	-0.22676	8.6731	142.95	0.0016	-9.5198	-8.3907
	74->81	-0.33041					
	79->91	0.42289					
29	77->81	-0.2358	8.7280	142.05	0.0045	-5.1422	-6.2796
	77->82	-0.242					
	77->85	0.3301					
30	73->80	-0.34309	8.7780	141.24	0.0073	-18.9327	-18.0927
	74->80	0.25068					
	78->85	0.36417					
31	73->80	0.312	8.7924	141.01	0.0096	3.3341	2.9554
	78->85	0.36082					
32	72->81	0.31739	8.8311	140.40	0.0063	17.1481	15.131
	76->81	0.24915					
33	71->80	0.32769	8.8566	139.99	0.0001	-0.3928	-0.5449
	75->80	-0.32225					
34	78->86	-0.34537	8.8727	139.74	0.0014	1.8019	2.1134
	78->87	0.38826					
35	79->88	0.3974	8.9073	139.19	0.0048	7.0375	7.172
	79->89	0.28982					
	79->90	0.33394					
36	77->83	0.25705	8.9998	137.76	0.0067	4.9856	3.4418
	77->86	-0.31471					
	79->92	0.34034					

[a]Number of the excited states; [b]Only transitions with contribution over 10.0% were listed; [c]Configuration-interaction coefficient; [d]Excitation energy; [e]Wavelength; [f]Oscillator strength; [g]Rotatory strength in velocity form (10^{-40} cgs); [h]Rotatory strength in length form (10^{-40} cgs).

Table S15. Key transitions, oscillator strengths, and rotatory strengths in the ECD spectrum of conformer 3-2 at the CAM-B3LYP/6-311G(d) level of theory in MeOH with IEFPCM solvent model.

Num[a]	*Transition*[b]	*CI-coeff*[c]	*ΔE (eV)*[d]	*λ (nm)*[e]	*f*[f]	R_{vel}[g]	R_{len}[h]
1	78->81	-0.28741	5.0384	246.08	0.1043	5.649	5.4971
	79->80	0.63991					
2	78->80	0.37667	5.7822	214.42	0.0516	14.7656	14.1898
	79->81	0.59021					
3	79->83	0.59264	6.5179	190.22	0.0282	-22.6557	-34.4852
4	78->80	0.55653	6.5790	188.46	0.5370	2.7276	14.4272
	79->81	-0.35447					
5	78->81	0.62751	6.6425	186.65	0.9565	7.3541	10.6742
	79->80	0.28671					
6	79->82	0.46072	7.2236	171.64	0.0050	-7.2242	-8.1113
	79->84	0.43476					
7	76->80	0.58526	7.4310	166.85	0.0057	-0.1269	-0.1005
8	77->82	0.55091	7.4743	165.88	0.0119	10.7222	6.3053
9	78->83	-0.24496	7.5327	164.59	0.0100	7.255	10.8263
	79->82	-0.28589					
	79->84	0.45688					
10	75->80	0.42593	7.5737	163.70	0.0038	-7.3662	-9.0608
	77->80	-0.42245					
11	78->82	-0.24051	7.6107	162.91	0.0023	-4.436	-4.043
	78->83	0.52093					
12	79->85	0.23149	7.7902	159.15	0.0028	13.1097	12.0901
	79->86	0.30069					
	79->87	0.25726					
	79->88	-0.29842					
13	76->81	0.55021	7.8074	158.80	0.0011	-1.8263	-1.1151
14	78->84	0.27463	7.8975	156.99	0.0036	-4.8827	-4.0531
	79->85	0.45018					
	79->87	-0.23506					
	79->89	-0.2328					
15	75->81	-0.42939	7.9216	156.51	0.0011	-4.8949	-4.3868
	77->81	0.45496					
16	78->82	0.35812	8.0290	154.42	0.0053	-6.1796	-6.7129
	78->84	0.41136					
	78->86	0.22771					

17	79->87	0.28508	8.1667	151.82	0.0074	10.9312	9.8652
	79->88	0.47706					
18	73->80	0.30208	8.2213	150.81	0.0051	-5.9749	-6.0768
	74->80	0.31832					
	78->82	0.22953					
	78->84	-0.29155					
19	73->80	0.28902	8.2574	150.15	0.0263	1.6264	1.567
	74->80	0.33291					
	78->84	0.29225					
20	70->80	0.23595	8.2892	149.57	0.0030	9.3252	9.6272
	71->80	-0.26775					
	77->80	0.42286					
21	79->82	0.23984	8.3728	148.08	0.0013	-0.635	-2.8979
	79->86	-0.26404					
	79->89	0.35684					
22	79->86	0.34681	8.4178	147.29	0.0065	-5.6971	-5.4953
	79->87	-0.33515					
	79->89	0.2723					
23	70->80	-0.23402	8.4664	146.44	0.0023	-10.3676	-8.688
	72->80	0.27499					
	76->80	0.23226					
24	78->86	-0.24671	8.4928	145.99	0.0020	11.8839	10.9308
	78->88	0.25452					
25	73->81	0.27623	8.5440	145.11	0.0071	3.0427	3.2029
	74->81	0.279					
	79->91	0.44705					
26	77->81	0.26931	8.5754	144.58	0.0044	6.2802	0.7524
	77->82	-0.22513					
	77->87	0.29121					
27	75->80	0.30799	8.6315	143.64	0.0025	10.6634	10.7796
	79->91	0.22456					
28	76->82	0.50407	8.6505	143.33	0.0306	11.2718	13.0638
29	73->81	0.30029	8.6745	142.93	0.0024	-1.4281	-1.5421
	74->81	0.28433					
	79->91	-0.27987					
30	75->82	-0.24406	8.7079	142.38	0.0039	-3.5762	-3.2737
	77->81	0.29497					
31	78->85	0.48192	8.7864	141.11	0.0085	-10.9556	-10.9286

	78->87	-0.2813					
32	70->80	0.28676	8.7993	140.90	0.0011	-3.2871	-3.3221
	73->80	0.35647					
	74->80	-0.2609					
33	70->81	-0.2605	8.8775	139.66	0.0296	-25.6458	-25.461
	73->81	0.26478					
	74->81	-0.25068					
	76->81	0.22729					
34	79->92	0.45375	8.9333	138.79	0.0035	7.3266	8.4288
	79->94	-0.22499					
35	78->87	0.30354	8.9633	138.32	0.0003	0.3668	0.3459
	78->88	0.34929					
36	77->85	0.31986	8.9895	137.92	0.0069	18.28	15.0036

[a]Number of the excited states; [b]Only transitions with contribution over 10.0% were listed; [c]Configuration-interaction coefficient; [d]Excitation energy; [e]Wavelength; [f]Oscillator strength; [g]Rotatory strength in velocity form (10^{-40} cgs); [h]Rotatory strength in length form (10^{-40} cgs).

Table S16. Key transitions, oscillator strengths, and rotatory strengths in the ECD spectrum of conformer 3-3 at the CAM-B3LYP/6-311G(d) level of theory in MeOH with IEFPCM solvent model.

Num[a]	*Transition*[b]	*CI-coeff*[c]	*ΔE (eV)*[d]	*λ (nm)*[e]	*f*[f]	R_{vel}[g]	R_{len}[h]
1	78->81	-0.29434	5.0433	245.84	0.0977	2.8421	2.5157
	79->80	0.63745					
2	78->80	0.37083	5.7697	214.89	0.0474	6.767	6.6106
	79->81	0.59436					
3	78->80	-0.30959	6.5573	189.08	0.1278	5.7957	29.4623
	79->83	0.50612					
4	78->80	0.50623	6.5676	188.78	0.4109	11.1208	-12.8075
	79->81	-0.31763					
	79->83	0.31627					
5	78->81	0.63092	6.6260	187.12	1.0047	-15.3179	-17.2012
	79->80	0.29606					
6	79->82	0.45317	7.2540	170.92	0.0072	-5.278	-6.0097
	79->84	0.42689					
	79->87	0.23194					
7	75->80	-0.36348	7.3446	168.81	0.0066	-1.4903	-1.5619

	77->80	0.50482					
8	79->82	-0.32257	7.4702	165.97	0.0062	-0.7673	-1.266
	79->84	0.49742					
9	76->80	-0.23552	7.6032	163.07	0.0092	-0.6551	4.8998
	77->82	0.46085					
10	76->80	0.42458	7.6249	162.60	0.0053	-9.1709	-10.0201
	78->83	-0.29234					
11	76->80	0.26001	7.6475	162.12	0.0023	-4.4808	-5.1061
	78->82	-0.25729					
	78->83	0.43616					
12	75->81	-0.34491	7.7029	160.96	0.0046	-3.8504	-3.5305
	77->81	0.49502					
13	79->86	0.41072	7.7839	159.28	0.0002	0.8164	1.0295
	79->88	0.25927					
	79->89	0.31922					
14	78->84	0.46922	7.9117	156.71	0.0048	-4.2505	-3.0328
	79->87	-0.27597					
15	76->81	0.47557	7.9646	155.67	0.0037	-9.8047	-9.9266
	79->85	0.28832					
16	76->81	-0.27223	7.9804	155.36	0.0115	14.38	15.0932
	79->85	0.48336					
17	78->84	0.25463	8.0831	153.39	0.0036	6.9768	7.3893
	79->86	-0.24929					
	79->87	0.45539					
18	78->82	0.41478	8.1723	151.71	0.0188	-1.5386	-2.7414
	78->83	0.30906					
	78->84	-0.2749					
19	74->80	0.48442	8.2220	150.80	0.0155	3.2851	3.7337
20	72->80	0.26595	8.2624	150.06	0.0026	4.5634	3.4675
	73->80	0.32132					
	74->80	0.24451					
	75->80	0.24861					
	77->80	0.28274					
21	71->80	0.27376	8.3785	147.98	0.0029	7.417	6.7225
	72->80	0.38899					
22	79->86	0.23299	8.4072	147.47	0.0008	7.1905	5.1249
	79->88	-0.23829					
	79->90	0.38835					

23	74->81	0.29294	8.4552	146.64	0.0142	6.5324	5.0403
24	78->86	0.29169	8.4816	146.18	0.0056	-2.82	-2.3593
	78->88	0.23006					
	78->89	0.23696					
25	72->81	0.26544	8.5463	145.07	0.0013	-7.9355	-5.5935
	75->81	0.28027					
	77->81	0.2967					
	77->85	0.23325					
26	76->82	0.47902	8.6068	144.05	0.0011	-1.7363	0.301
27	76->82	-0.29299	8.6180	143.87	0.0051	-21.5984	-18.7978
	79->90	0.35922					
28	77->82	-0.25183	8.7081	142.38	0.0061	1.8387	0.7366
	77->85	0.35276					
29	73->80	0.38648	8.7430	141.81	0.0012	-3.9584	-5.5337
	74->80	-0.24354					
	79->91	-0.33864					
30	73->80	0.23898	8.7822	141.18	0.0082	-5.5221	-5.0454
	73->81	0.23764					
	79->91	0.43274					
31	72->81	0.29842	8.7834	141.16	0.0035	9.6881	7.9849
	75->81	-0.22375					
32	78->85	0.51066	8.8054	140.80	0.0121	0.861	1.287
	78->87	0.26798					
33	70->80	0.30157	8.8580	139.97	0.0032	-6.1621	-5.9568
	75->80	-0.26899					
34	78->86	-0.25445	8.8682	139.81	0.0004	1.2201	1.2494
	78->87	0.33748					
	79->89	-0.24043					
35	78->86	0.29277	8.8836	139.57	0.0038	15.1382	15.8822
	78->87	-0.26899					
	79->89	-0.22371					
36	67->80	0.23973	8.9500	138.53	0.0029	0.016	0.734
	71->80	0.39713					

[a]Number of the excited states; [b]Only transitions with contribution over 10.0% were listed; [c]Configuration-interaction coefficient; [d]Excitation energy; [e]Wavelength; [f]Oscillator strength; [g]Rotatory strength in velocity form (10^{-40} cgs); [h]Rotatory strength in length form (10^{-40} cgs).

Table S17. Key transitions, oscillator strengths, and rotatory strengths in the ECD spectrum of conformer 3-4 at the CAM-B3LYP/6-311G(d) level of theory in MeOH with IEFPCM solvent model.

Num[a]	*Transition*[b]	*CI-coeff*[c]	*ΔE (eV)*[d]	*λ (nm)*[e]	*f*[f]	R_{vel}[g]	R_{len}[h]
1	78->81	-0.29111	5.0374	246.13	0.0947	6.1523	6.0515
	79->80	0.63671					
2	78->80	0.37552	5.7718	214.81	0.0438	10.0239	9.6797
	79->81	0.58985					
3	78->80	0.50932	6.5317	189.82	0.2652	-49.3687	-63.9984
	79->81	-0.33076					
	79->83	-0.24879					
4	78->80	0.25052	6.6061	187.68	0.9713	-68.536	-68.1676
	78->81	0.49642					
	79->80	0.24206					
	79->83	0.23878					
5	78->81	-0.37341	6.6823	185.54	0.3045	122.0551	144.083
	79->82	0.30407					
	79->83	0.39416					
6	79->82	0.46732	7.2688	170.57	0.0075	-2.589	-3.9791
	79->84	0.38881					
7	75->80	-0.3117	7.3629	168.39	0.0085	-4.5756	-4.8532
	77->80	0.50292					
8	76->80	0.36638	7.5641	163.91	0.0100	11.5249	16.0733
	77->82	0.23022					
	79->84	-0.28903					
9	76->80	0.37839	7.5874	163.41	0.0031	-6.582	-6.777
	79->83	0.214					
	79->84	0.3484					
10	77->82	0.37542	7.6271	162.56	0.0057	-11.1524	-10.7089
	77->83	-0.26771					
	79->84	0.24354					
11	75->81	-0.22284	7.7269	160.46	0.0009	2.0012	1.5643
	77->81	0.37005					
	79->85	-0.30895					
12	77->81	0.30343	7.7459	160.07	0.0055	-6.8457	-6.6847
	78->83	0.26594					
	79->85	0.29334					
13	78->82	0.41057	7.7653	159.66	0.0005	0.0014	-0.0311

	78->83	0.41599					
14	76->81	0.5136	7.9309	156.33	0.0013	-2.2518	-2.1584
	77->81	-0.24677					
15	79->85	-0.26028	7.9849	155.27	0.0188	12.2492	13.1375
	79->86	0.58944					
16	78->82	0.35333	8.0199	154.59	0.0044	-6.5251	-6.2877
	78->83	-0.28622					
	78->84	0.29752					
	78->85	0.3165					
17	78->84	0.53546	8.1793	151.58	0.0140	-7.1093	-6.614
18	73->80	0.34709	8.2420	150.43	0.0077	-1.3103	-4.055
	74->80	0.37176					
	79->87	0.32082					
19	73->80	-0.23171	8.2612	150.08	0.0067	12.0655	12.3197
	74->80	-0.29495					
	79->87	0.44382					
20	71->80	0.38557	8.3117	149.17	0.0025	-6.4874	-6.3808
	75->80	0.30616					
	77->80	0.36645					
21	69->80	0.28118	8.3910	147.76	0.0059	4.1679	3.7634
	72->80	0.26696					
	76->80	0.28197					
22	79->90	0.48512	8.4050	147.51	0.0037	7.0965	6.6749
23	78->83	0.22095	8.4124	147.38	0.0084	14.447	14.6532
	78->85	0.31837					
	79->90	0.27141					
24	73->81	0.29724	8.4837	146.14	0.0081	9.2933	4.2095
	74->81	0.27534					
	79->89	0.27428					
25	79->88	0.35934	8.5891	144.35	0.0036	-4.4273	-5.4266
	79->90	0.23383					
26	77->82	-0.24033	8.5961	144.23	0.0012	-12.707	-9.9294
	77->86	0.35036					
27	76->82	0.43189	8.6168	143.89	0.0008	-4.1515	-2.576
	76->83	-0.26049					
28	73->81	0.24621	8.6761	142.90	0.0006	0.1758	3.3433
	74->81	0.28047					
	79->89	-0.27654					

	79->91	0.35463					
29	77->86	0.21332	8.7346	141.95	0.0066	-12.1962	-13.3717
30	73->80	0.39894	8.7567	141.59	0.0015	-6.9114	-6.6564
	74->80	-0.30132					
31	69->81	0.21578	8.7894	141.06	0.0071	0.4768	-0.3969
	78->86	0.2376					
32	78->85	-0.25221	8.8178	140.61	0.0112	5.3499	6.0252
	78->86	0.50033					
33	71->80	0.39453	8.8698	139.78	0.0006	1.4774	1.433
	75->80	-0.36674					
34	77->88	-0.23666	8.9868	137.96	0.0123	-6.3849	-8.6163
	79->88	-0.30898					
	79->89	0.34765					
	79->91	0.25947					
35	78->87	0.25847	9.0187	137.48	0.0024	-7.0299	-6.6967
	79->92	0.47913					
36	77->84	0.32248	9.0211	137.44	0.0036	3.9796	1.9725
	77->85	-0.24892					
	78->87	-0.24861					

[a]Number of the excited states; [b]Only transitions with contribution over 10.0% were listed; [c]Configuration-interaction coefficient; [d]Excitation energy; [e]Wavelength; [f]Oscillator strength; [g]Rotatory strength in velocity form (10^{-40} cgs); [h]Rotatory strength in length form (10^{-40} cgs).

Table S18. Key transitions, oscillator strengths, and rotatory strengths in the ECD spectrum of conformer 3-5 at the CAM-B3LYP/6-311G(d) level of theory in MeOH with IEFPCM solvent model.

Num[a]	***Transition***[b]	***CI-coeff***[c]	***ΔE (eV)***[d]	***λ (nm)***[e]	***f***[f]	***R_{vel}***[g]	***R_{len}***[h]
1	78->81	-0.29108	5.0374	246.13	0.0947	6.1339	6.0333
	79->80	0.63672					
2	78->80	0.37571	5.7720	214.80	0.0438	10.0085	9.664
	79->81	0.58973					
3	78->80	0.5094	6.5315	189.82	0.2655	-49.238	-63.868
	79->81	-0.33103					
	79->83	-0.24852					
4	78->80	0.25006	6.6061	187.68	0.9709	-68.5958	-68.2364
	78->81	0.49652					

	79->80	0.24209					
	79->83	0.23887					
5	78->81	-0.37345	6.6824	185.54	0.3046	122.0636	144.1006
	79->82	0.30437					
	79->83	0.39386					
6	79->82	0.46723	7.2691	170.56	0.0075	-2.6005	-3.9945
	79->84	0.38875					
7	75->80	-0.31152	7.3630	168.39	0.0085	-4.5636	-4.8413
	77->80	0.50257					
8	76->80	0.36768	7.5641	163.91	0.0100	11.5468	16.0717
	77->82	0.22907					
	79->84	-0.28822					
9	76->80	0.37659	7.5873	163.41	0.0031	-6.5755	-6.7595
	79->83	0.21513					
	79->84	0.35011					
10	77->82	0.3759	7.6272	162.55	0.0057	-11.0934	-10.6385
	77->83	-0.26843					
	79->84	0.24216					
11	75->81	-0.21975	7.7272	160.45	0.0009	1.9572	1.5034
	77->81	0.36471					
	79->85	-0.31378					
12	77->81	0.30824	7.7460	160.06	0.0055	-6.708	-6.5388
	78->83	0.26631					
	79->85	0.28739					
13	78->82	0.41018	7.7653	159.66	0.0005	-0.0625	-0.088
	78->83	0.41458					
14	76->81	0.51308	7.9312	156.33	0.0013	-2.252	-2.1597
	77->81	-0.24757					
15	79->85	-0.26115	7.9847	155.28	0.0188	12.247	13.1338
	79->86	0.58915					
16	78->82	0.35321	8.0199	154.60	0.0044	-6.5102	-6.2748
	78->83	-0.28662					
	78->84	0.2972					
	78->85	0.31631					
17	78->84	0.53572	8.1791	151.59	0.0140	-7.094	-6.5955
18	73->80	0.34856	8.2420	150.43	0.0078	-1.3257	-4.0781
	74->80	0.3752					
	79->87	-0.31697					

19	73->80	0.22803	8.2613	150.08	0.0066	11.9712	12.245
	74->80	0.2921					
	79->87	0.44661					
20	71->80	0.38649	8.3118	149.17	0.0025	-6.3934	-6.298
	75->80	0.30655					
	77->80	0.3666					
21	69->80	0.28176	8.3908	147.76	0.0060	4.2454	3.8248
	72->80	0.26807					
	76->80	0.2834					
22	79->90	0.48154	8.4050	147.51	0.0036	6.9928	6.6271
23	78->83	0.21962	8.4123	147.38	0.0083	14.3632	14.5404
	78->85	0.31447					
	79->90	0.28207					
24	73->81	0.29681	8.4841	146.14	0.0081	9.3295	4.249
	74->81	0.2758					
	79->89	0.27437					
25	79->88	0.35976	8.5896	144.34	0.0036	-4.449	-5.5093
	79->90	0.23365					
26	77->82	-0.24103	8.5961	144.23	0.0011	-12.7294	-9.8656
	77->86	0.35153					
27	76->82	0.42997	8.6171	143.88	0.0008	-4.0287	-2.483
	76->83	-0.25954					
28	73->81	0.24556	8.6764	142.90	0.0006	0.1024	3.285
	74->81	0.2801					
	79->89	-0.27669					
	79->91	0.35486					
29	77->86	0.21242	8.7350	141.94	0.0066	-12.219	-13.3982
30	73->80	0.39862	8.7567	141.59	0.0015	-6.9366	-6.6788
	74->80	-0.30041					
31	69->81	0.21525	8.7893	141.06	0.0072	0.4253	-0.4481
	78->86	0.23902					
32	78->85	-0.25244	8.8173	140.61	0.0111	5.2978	5.973
	78->86	0.49922					
33	71->80	0.39467	8.8693	139.79	0.0006	1.5599	1.5147
	75->80	-0.36672					
34	77->88	-0.23746	8.9869	137.96	0.0123	-6.3248	-8.5678
	79->88	-0.30918					
	79->89	0.34738					

	79->91	0.26028					
35	78->87	-0.26315	9.0186	137.48	0.0025	-7.1541	-6.8236
	79->92	0.47693					
36	77->84	0.3237	9.0212	137.44	0.0036	3.9716	1.9509
	77->85	-0.25021					
	78->87	0.24403					

[a]Number of the excited states; [b]Only transitions with contribution over 10.0% were listed; [c]Configuration-interaction coefficient; [d]Excitation energy; [e]Wavelength; [f]Oscillator strength; [g]Rotatory strength in velocity form (10^{-40} cgs); [h]Rotatory strength in length form (10^{-40} cgs).

Table S19. Key transitions, oscillator strengths, and rotatory strengths in the ECD spectrum of conformer 3-6 at the CAM-B3LYP/6-311G(d) level of theory in MeOH with IEFPCM solvent model.

Num[a]	*Transition*[b]	*CI-coeff*[c]	*ΔE (eV)*[d]	*λ (nm)*[e]	*f*[f]	R_{vel}[g]	R_{len}[h]
1	78->81	-0.29379	5.0434	245.83	0.0987	3.261	2.9046
	79->80	0.63763					
2	78->80	0.36919	5.7699	214.88	0.0480	6.3216	6.1566
	79->81	0.59525					
3	79->83	0.6119	6.5452	189.43	0.0129	11.5409	21.8291
4	78->80	0.58435	6.5697	188.72	0.5063	4.8266	-5.8639
	79->81	-0.36376					
5	78->81	0.63131	6.6276	187.07	1.0293	-13.9117	-15.8024
	79->80	0.29575					
6	79->82	0.41781	7.2402	171.24	0.0062	-6.0541	-6.3892
	79->84	0.47288					
7	76->80	0.57612	7.4177	167.15	0.0055	1.2967	1.3139
8	79->82	-0.3289	7.4587	166.23	0.0082	-9.5941	-12.7426
	79->84	0.42571					
	79->85	-0.23964					
9	77->82	0.52834	7.4776	165.81	0.0066	13.9507	12.8982
10	75->80	-0.41961	7.5874	163.41	0.0040	-5.9662	-7.4484
	77->80	0.43637					
11	78->82	-0.244	7.6283	162.53	0.0014	-2.7491	-2.688
	78->83	0.55635					
12	76->81	0.47313	7.7691	159.59	0.0036	9.2087	8.5262
	79->86	-0.23629					

	79->88	0.23736					
13	76->81	0.36122	7.7838	159.29	0.0010	-1.6247	-1.3511
	79->86	0.31566					
	79->88	-0.2995					
14	78->84	0.4461	7.8896	157.15	0.0050	-5.4679	-4.3762
	79->85	-0.30189					
	79->87	-0.23275					
15	75->81	-0.41838	7.9183	156.58	0.0011	-4.3427	-3.6446
	77->81	0.45929					
16	78->84	0.36849	8.0168	154.66	0.0042	-5.891	-6.5835
	79->85	0.33387					
	79->87	0.31309					
17	78->82	0.45277	8.1622	151.90	0.0178	-14.5926	-14.0512
	78->83	0.25697					
	78->85	0.25407					
18	79->86	0.23769	8.1848	151.48	0.0085	9.2928	9.7257
	79->87	0.24268					
	79->88	0.43383					
19	73->80	0.38657	8.2386	150.49	0.0187	12.4816	12.5098
	74->80	0.46937					
20	72->80	-0.34343	8.2978	149.42	0.0012	3.2107	4.1394
	77->80	0.3762					
21	79->82	-0.23831	8.3831	147.90	0.0026	5.2901	1.739
	79->85	0.23177					
	79->86	0.26474					
	79->87	-0.23615					
	79->89	0.23133					
22	70->80	-0.24717	8.4367	146.96	0.0058	-18.8323	-16.8904
	72->80	0.23518					
	73->80	0.25292					
23	78->86	-0.2301	8.4607	146.54	0.0048	11.5957	10.7578
	78->88	0.26818					
	79->86	-0.22496					
24	78->86	-0.22616	8.4875	146.08	0.0080	15.5465	13.453
	78->88	0.25119					
25	77->81	0.25737	8.5637	144.78	0.0046	6.0732	0.2684
	77->87	0.2369					
26	70->80	0.27172	8.5994	144.18	0.0045	-13.6041	-9.3529

	71->80	-0.2401					
	75->80	0.27779					
	77->80	0.2291					
27	73->81	-0.24061	8.6101	144.00	0.0014	-6.5002	-4.4291
	74->81	-0.26231					
	79->89	0.29865					
28	76->82	0.51556	8.6537	143.27	0.0318	16.8098	15.8079
29	72->81	0.2586	8.6827	142.79	0.0041	-1.9933	-1.0873
	75->82	0.25706					
30	72->80	-0.32311	8.7279	142.05	0.0005	-1.1534	-1.0391
	73->80	0.33521					
	74->80	-0.23752					
	79->91	-0.26965					
31	79->91	0.52146	8.7654	141.45	0.0078	4.4441	5.1745
32	78->85	0.45668	8.7977	140.93	0.0100	-6.3225	-6.5337
	78->87	0.33199					
33	72->81	0.30711	8.8109	140.72	0.0111	-11.2528	-11.6701
	73->81	0.25115					
	75->81	0.26019					
	77->81	0.24692					
34	79->89	0.30773	8.8753	139.70	0.0084	5.1521	5.5986
	79->92	0.35592					
	79->96	0.24057					
35	77->86	0.27056	8.9648	138.30	0.0007	3.5301	2.7605
	78->88	-0.26078					
36	79->90	0.31976	8.9670	138.27	0.0036	6.8609	6.4594
	79->92	0.364					
	79->94	-0.25787					

[a]Number of the excited states; [b]Only transitions with contribution over 10.0% were listed; [c]Configuration-interaction coefficient; [d]Excitation energy; [e]Wavelength; [f]Oscillator strength; [g]Rotatory strength in velocity form (10^{-40} cgs); [h]Rotatory strength in length form (10^{-40} cgs).

Table S20. Key transitions, oscillator strengths, and rotatory strengths in the ECD spectrum of conformer 3-7 at the CAM-B3LYP/6-311G(d) level of theory in MeOH with IEFPCM solvent model.

Num[a]	*Transition*[b]	*CI-coeff*[c]	*ΔE (eV)*[d]	*λ (nm)*[e]	*f*[f]	R_{vel}[g]	R_{len}[h]
1	78->81	-0.29026	5.0363	246.18	0.0946	-1.0403	-1.3881
	79->80	0.63702					
2	78->80	0.37708	5.7721	214.80	0.0440	2.1541	2.4187
	79->81	0.58887					
3	78->80	0.50839	6.5216	190.11	0.2630	45.6525	59.1186
	79->81	-0.33191					
	79->83	0.29189					
4	78->80	0.23894	6.6060	187.68	1.0001	78.9432	77.9387
	78->81	0.5138					
	79->80	0.25014					
	79->83	-0.26655					
5	78->81	0.35096	6.6909	185.30	0.2740	-115.424	-141.4632
	79->83	0.49135					
6	79->82	0.49139	7.2883	170.11	0.0118	-11.3497	-11.8221
	79->84	0.36576					
7	75->80	-0.31367	7.3579	168.50	0.0054	1.4706	1.427
	77->80	0.50163					
8	76->80	-0.32884	7.5544	164.12	0.0061	2.2333	7.5831
	77->82	-0.26071					
	79->82	-0.22735					
	79->84	0.34309					
9	76->80	0.40818	7.5789	163.59	0.0044	-7.4632	-8.0829
	79->82	-0.23853					
	79->84	0.33603					
10	77->82	0.47104	7.6245	162.61	0.0079	-8.0604	-7.7998
11	77->81	-0.24	7.7181	160.64	0.0020	7.4555	7.5278
	79->85	0.39327					
	79->86	0.25092					
12	75->81	-0.24419	7.7433	160.12	0.0013	-6.1585	-4.5852
	77->81	0.40796					
13	78->83	0.61248	7.7787	159.39	0.0076	-7.454	-7.4936
14	76->81	0.50635	7.9299	156.35	0.0010	-1.2255	-0.8252
	77->81	-0.25637					
15	79->85	-0.30716	7.9820	155.33	0.0169	12.5981	13.8375

	79->86	0.55863					
16	78->82	0.4591	8.0170	154.65	0.0087	-8.3061	-8.6613
	78->84	0.25315					
	78->85	0.3039					
17	78->84	0.55784	8.1766	151.63	0.0157	-0.3626	-0.9832
18	73->80	0.28507	8.2158	150.91	0.0152	7.1516	7.691
	74->80	0.49859					
19	79->87	0.53372	8.2473	150.33	0.0008	4.9081	5.0586
	79->88	0.22834					
20	71->80	0.42813	8.3301	148.84	0.0045	-1.0413	-0.9444
	75->80	0.30776					
	77->80	0.33962					
21	78->82	-0.29225	8.3967	147.66	0.0047	6.8959	6.788
	78->85	0.34062					
	78->86	0.23765					
22	69->80	0.27117	8.4122	147.39	0.0045	10.8283	12.022
	72->80	0.37152					
	76->80	0.26753					
23	79->90	0.6338	8.4261	147.14	0.0022	0.1042	1.271
24	73->81	0.23017	8.5081	145.73	0.0049	1.0441	0.1591
	74->81	0.35254					
	79->89	0.27615					
25	74->81	-0.27318	8.5576	144.88	0.0026	-2.8871	-4.4174
	79->88	0.35563					
26	76->82	0.42342	8.6030	144.12	0.0003	-4.8887	-1.0481
	77->82	-0.2287					
	77->86	0.29912					
27	72->81	-0.22607	8.6111	143.98	0.0018	-7.5201	-6.8513
	76->82	0.37081					
28	79->89	-0.30327	8.7252	142.10	0.0031	-9.3994	-6.2745
	79->91	0.35848					
29	73->80	0.42771	8.7337	141.96	0.0045	-11.8101	-11.0753
	74->80	-0.28183					
30	73->80	0.2818	8.7600	141.53	0.0010	-5.9951	-7.6567
31	69->81	0.23775	8.7955	140.96	0.0020	5.703	5.3967
	72->81	0.30661					
	76->81	0.22934					
32	78->85	-0.27139	8.8067	140.78	0.0150	9.8974	9.3666

	78->86	0.49733					
33	71->80	0.38843	8.8643	139.87	0.0054	-5.7318	-4.7713
	75->80	-0.36581					
34	79->91	-0.34923	8.9237	138.94	0.0073	5.1782	5.5792
	79->92	0.36888					
	79->95	-0.22848					
35	77->88	0.25855	9.0040	137.70	0.0067	-11.3345	-12.3977
	78->87	0.32958					
36	77->84	0.28134	9.0246	137.39	0.0139	1.0542	0.8147
	77->85	-0.22755					
	79->92	0.24999					

[a]Number of the excited states; [b]Only transitions with contribution over 10.0% were listed; [c]Configuration-interaction coefficient; [d]Excitation energy; [e]Wavelength; [f]Oscillator strength; [g]Rotatory strength in velocity form (10^{-40} cgs); [h]Rotatory strength in length form (10^{-40} cgs).
